# Click chemistry enables quantitative chiroptical sensing of chiral compounds in protic media and complex mixtures

**DOI:** 10.1038/s41467-018-07695-9

**Published:** 2018-12-14

**Authors:** F. Yushra Thanzeel, Kaluvu Balaraman, Christian Wolf

**Affiliations:** Department of Chemistry, Georgetown University, 37th and O Streets, Washington, DC 20057 USA

## Abstract

Click reactions have become powerful synthetic tools with unique applications in the health and materials sciences. Despite the progress with optical sensors that exploit the principles of dynamic covalent chemistry, metal coordination or supramolecular assemblies, quantitative analysis of complex mixtures remains challenging. Herein, we report the use of a readily available coumarin conjugate acceptor for chiroptical click chirality sensing of the absolute configuration, concentration and enantiomeric excess of several compound classes. This method has several attractive features, including wide scope, fast substrate fixation without by-product formation or complicate equilibria often encountered in reversible substrate binding, excellent solvent compatibility, and tolerance of air and water. The ruggedness and practicality of this approach are demonstrated by comprehensive analysis of nonracemic monoamine samples and crude asymmetric imine hydrogenation mixtures without work-up. Click chemosensing addresses increasingly important time efficiency, cost, labor and chemical sustainability aspects and streamlines asymmetric reaction development at the mg scale.

## Abstract (web summary)

Chiroptical sensing in complex mixtures remains a challenging task. Here, the authors report an efficient coumarin probe for chiroptical click chirality sensing of absolute configuration, concentration and enantiomeric excess of several compound classes. The method can be directly applied to crude asymmetric reaction mixtures.

## Introduction

Chirality plays an essential role across the chemical and pharmaceutical sciences, and the development of new methods for the synthesis and analysis of chiral compounds are frequently required tasks in academic and industrial laboratories. To accelerate the discovery process, it has become routine to perform hundreds of small-scale reactions in parallel using widely available high-throughput experimentation equipment (HTE)^1,2^. With regard to asymmetric reaction development, many combinations of different chiral catalysts, solvents, additives, and other parameters typically need to be evaluated. In the search for an optimized procedure, a chemist can easily alter a large set of reaction parameters and produce hundreds of chiral samples in a very short time using multiwell plate technology. In stark contrast with automated synthesis capabilities, the determination of the absolute configuration, yield and enantiomeric excess of asymmetric reactions with traditional chromatographic methods that are serial in nature and incompatible with HTE remains slow, and this has shifted increasing attention to contemporary screening techniques^3^. Optical methods are compatible with parallel data acquisition, miniaturization, and multiwell plate formats and offer a new path to real high-throughput analysis of chiral samples^4–7^. Few examples of asymmetric reaction analysis with sensors operating on the principles of dynamic covalent chemistry^8–11^, metal complex coordination^12^, and supramolecular chemistry^13–15^ to recognize a chiral target compound and to generate quantifiable ultraviolet (UV), fluorescence, and circular dichroism (CD) signals have been reported^16–18^. Comprehensive chirality sensing (CCS), i.e., determination of the absolute configuration, yield, and enantiomeric excess (*ee*), of crude asymmetric reaction mixtures via irreversible covalent product fixation has been largely neglected to date^19^.

More than 10 years ago, Sharpless coined the term “click chemistry” for reactions that are high yielding, practical and operationally safe, avoid by-product formation, proceed in environmentally benign solvents at room temperature and generally under mild conditions, and eliminate chromatographic work-up^20^. Since then, a variety of reaction strategies and applications that exploit this concept, in particular in the biomedical domain, have been introduced^21–27^. The inherent robustness and practicality of click chemistry encouraged us to explore probe designs that overcome drawbacks of currently used chiroptical sensing methods such as limited substrate scope (amine sensors often utilize reversible Schiff base formation and are restricted to primary substrates), competing chiral recognition processes and equilibria that complicate the analysis and diminish CD readouts, and sensitivity to moisture, air, and other chemical interferences, which limits the usefulness when complex mixtures need to be examined. We now introduce a rugged, readily available, easy to use, inexpensive click sensor that is expected to increase the speed of scientific discoveries in countless laboratories through streamlined enantioselective analysis of scalemic samples and accelerated asymmetric reaction development.

## Results and discussion

### Sensor development studies

**Fig. 1:**
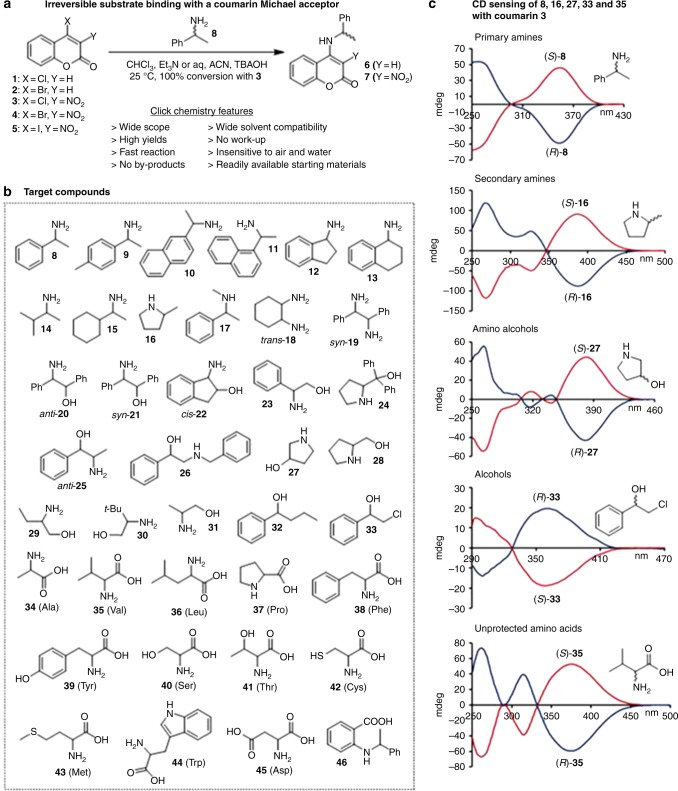
Chiroptical click chemistry sensing. **a** Concept and features; **b** target scope; and **c** selected examples of the distinct CD signals obtained at high wavelengths using **3** as probe. All CD measurements were taken in CHCl_3_ (**8**, **16**, **27**, and **33**) or aqueous acetonitrile (**35**) at 0.24 mM

Our search for a suitable chromophoric probe that can quickly and quantitatively capture a variety of chiral target compounds through irreversible covalent bond formation at room temperature led us to prepare and investigate the coumarin-derived Michael acceptors **1**–**5** (Fig. 1, Supplementary Methods). Initial screening with chiral amines revealed that these 4-halocoumarins undergo smooth Michael addition and subsequent halide elimination toward **6** and **7**, respectively. Further analysis of the chiroptical properties showed that the covalent attachment of 1-phenylethylamine, **8**, which was one of the initially tested amines, to the coumarin scaffold results in a strong CD signal and a distinct UV change at high wavelengths at micromolar concentrations. Importantly, the Michael addition/elimination substrate binding strategy does not generate a new chirality center. In contrast to other sensor designs, this feature avoids complications arising from the formation of diastereomeric mixtures which simplifies the chirality sensing protocol described below. Comparison of the reactivity and chiroptical responses of the five probes revealed superior properties of 4-chloro-3-nitrocoumarin, **3** (see Supplementary Figures 8–13). When this sensor is employed, the reaction proceeds quantitatively at room temperature without by-product formation in various solvents ranging from chloroform to aqueous acetonitrile. The presence of the auxochromic nitro group is important for two reasons: it significantly accelerates the covalent substrate fixation and it affords a stronger Cotton effect at higher wavelengths (Supplementary Figures 8 and 9).

The scope of this sensor turned out to be very large. Encouraged by the practicality of the sensing assay and the large Cotton effects observed upon binding of **8**, we decided to apply coumarin **3** to a variety of amines, amino alcohols, alcohols and amino acids, including aspartic acid, threonine, cysteine, and others with functionalized sidechains. We obtained distinct chiroptical responses in all cases (Supplementary Figures 29–69). Representative CD spectra are shown in Fig. 1. The binding of the *S*-enantiomers of the amines, amino alcohols and amino acids **8**, **16**, **27**, and **35** yields a positive Cotton effect above 300 nm whereas the *R*-substrates induce the opposite CD response. The reverse relationship between the absolute configuration and the sign of the induced Cotton effects was observed using the alcohol **33** as the sensing target. It is noteworthy that among the 39 sensing targets are several with a secondary amino group, i.e., **16**, **17**, **24**, **27**, **28**, **37,** and **46**, that cannot be sensed via the commonly used Schiff base formation approach^9,28–34^. The successful sensing of **32**, **33**, and **46** exhibiting low nucleophilicity further underscores the wide utility of our 4-halocoumarin probes.

**Fig. 2:**
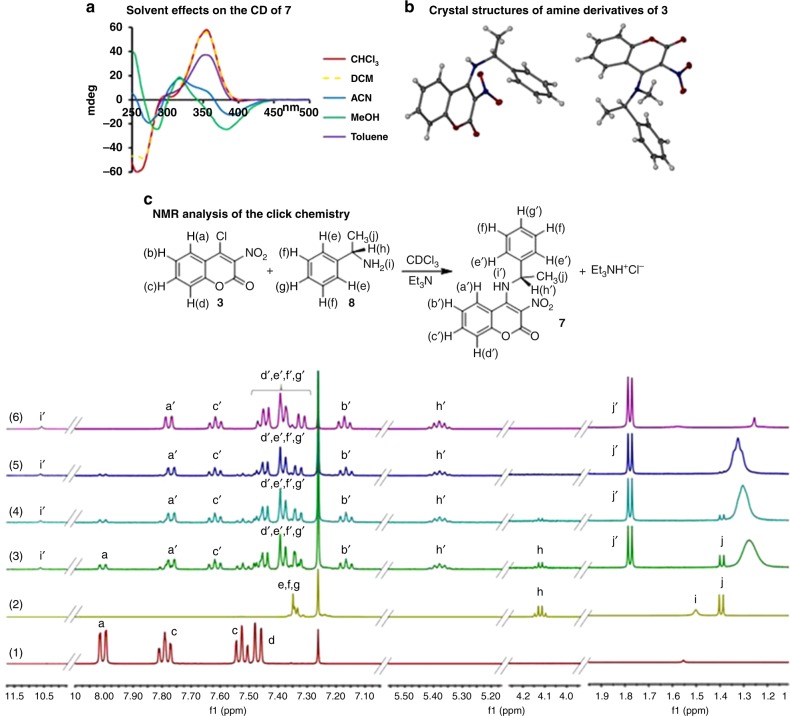
Analysis of the sensing chemistry. **a** The CD spectra of **7** were obtained at 0.19 or 0.24 mM when MeOH was used as solvent. **b** X-ray structures of chiral amine derivatives of **3**. **c** ^1^H NMR analysis of the reaction between probe **3** and (*S*)-**8** in the presence of Et_3_N (all 5.0 mM) in 0.8 mL of CDCl_3_. (1) Probe **3**; (2) (*S*)-1-phenylethylamine; (3) reaction mixture of (*S*)-1-phenylethylamine, Et_3_N and probe **3** after 5 min; (4) reaction after 10 min; (5) after 15 min; and (6) isolated 3-nitro-4-((1-phenylethyl)amino)coumarin, **7**, for comparison

Comparison of the chiroptical signals observed with the 4-halocoumarins **1** and **2** versus the nitro analogues **3**–**5** upon binding of **8** shows a strong contribution of the nitro dipole in the sensor scaffold to the CD intensity of the Michael addition/elimination product (see Supplementary Figures 8–13). The nitro group contribution results in a stronger and remarkably red-shifted CD signal which is advantageous for direct asymmetric reaction analysis because it eliminates interference from CD-active catalysts with the chiroptical measurements, vide infra. Although intramolecular hydrogen bonding (–NH···O_2_N–) is likely to occur with **7** in aprotic solvents it is not a prerequisite for this sensor to function. We obtained strong albeit quite different CD signatures using chloroform, dichloromethane, toluene, acetonitrile, and methanol, which is expected as the solvent choice can disturb the hydrogen bonding motif and alter conformational equilibria (Fig. 2, Supplementary Figures 14–16 and 28)^35^. In fact, intramolecular hydrogen bonding interactions are absent in the crystal structure of the primary amine addition product **7** and we observed very strong CD effects upon binding of substrates with secondary amino groups, for example **16**, which affords a product devoid of an NH donor site (Figs. 1 and SI). The sensing does also not require CD coupling events and can therefore be applied to monofunctional aliphatic substrates such as **14**–**16**. Altogether, these features result in a large scope which is highlighted by chirality sensing of 39 compounds. The inherently wide solvent compatibility is very attractive from an operational perspective because it simplifies adaption to asymmetric reaction conditions, for example when alcoholic co-solvents are used in catalytic enantioselective imine hydrogenations as described below. The sensing reaction and the corresponding CD effects were further investigated by UV, CD, and nuclear magnetic resonance (NMR) spectroscopy. We separately prepared the products of the reactions of **3** with **8**, **17** and *cis*-**22**, respectively. The CDs of these isolated compounds match those generated in the sensing assays (Supplementary Figures 7–27). The reaction between 1-phenylethylamine and probe **3** in the presence of Et_3_N was closely monitored by ^1^H NMR spectroscopy (Fig. 2). The spectra collected after 5, 10, and 15 min show the clean transformation of **3** and **8** into **7** which is complete after approximately 15 min at room temperature. For example, the signals at 1.39 ppm (H_j_) and 4.12 ppm (H_h_) of **8** undergo a downfield shift to 1.78 and 5.38 ppm, respectively, as **7** is formed. Accordingly, the doublet at 8.00 ppm (H_a_) of probe **3** shows an upfield shift to 7.78 ppm in the reaction mixture.

The sensing with **3** exhibits the main elements of click chemistry^19^: it is fast, wide in scope, displays smooth substrate transformation with very high yield at room temperature, is compatible with a wide range of environmentally benign solvents such as methanol and acetonitrile, avoids formation of by-products, eliminates chromatographic or any type of work-up, is insensitive to air and moisture, and utilizes readily available starting materials, i.e., the coumarin probe **3**. We anticipated that these preferable reaction characteristics in combination with the strong, red-shifted chiroptical readouts will generate unique sensing opportunities.

### Comprehensive chirality and concentration sensing

**Fig. 3:**
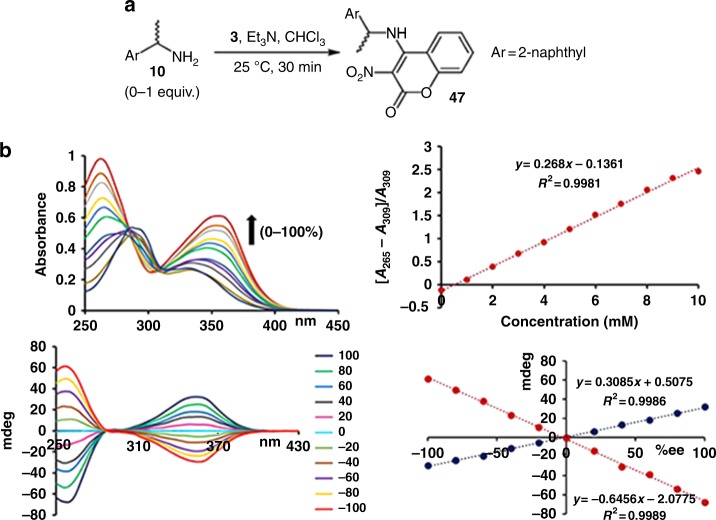
Chiroptical sensing of **10**. **a** UV response of **3** to varying amounts of **10**. **b** CD response of **3** to nonracemic samples of **10** and linear correlation between the induced CD signals at 257 (red) and 355 (blue) nm and the sample *ee*

A closer look at the sensing of 1-(2-naphthyl)ethylamine, **10**, revealed that the irreversible substrate binding concurs with a drastic UV increase at 265 and 355 nm, while the absorption at 309 nm remains unchanged (Fig. 3). This unique feature allows ratiometric sensing of the amine concentration, for example by using the ratio of the relative absorption increase at 265 nm compared to the UV signal at 309 nm. We then conducted CD experiments and discovered that the induced CD maxima at 257 and 355 nm increase linearly with the substrate *ee*. The absolute configuration of **10** or another target compound can be assigned based on comparison of the sign of the induced CD signals with a reference and quantitative information about the substrate amount (concentration) and its enantiomeric composition is directly accessible from the UV changes and the CD amplitudes, respectively. This is particularly attractive because modern CD instruments generate UV and CD spectra simultaneously.

The robustness of the fast and quantitative Michael addition/elimination chemistry with a wide variety of chiral compounds in combination with the distinct chiroptical readouts of the coumarin sensor **3** suggested to us that sensing of complex mixtures, for example crude asymmetric reaction mixtures containing a variety of typically interfering compounds such as a chiral catalyst, additives, by-products and protic solvents, was within reach. We first decided to verify that the UV/CD responses of the coumarin probe allow reliable assignment of the absolute configuration together with accurate determination of the *ee* and concentration of micromolar samples of **10**. Nine samples containing the amine in varying concentration and *ee* were prepared and subjected to our CCS assay (Table 1 and Supplementary Figures 70–74). In all cases, the absolute configuration of the major enantiomer was correctly identified by comparison of the CD signal with a reference and the concentrations and *ee*’s were determined with good accuracy. For example, the sensing analysis of the sample containing (*S*)-**10** in 33.3% *ee* at 2.50 μM determined that the *S*-enantiomer was present in 36.0% *ee* at 2.70 μM (entry 5, Table 1). Generally, the click chemistry with coumarin **3** is complete within 1 h when the derivatization is conducted at 5 mM concentration which is convenient for adaption to small-scale asymmetric reaction development. The chiroptical analysis can then be performed without delay after dilution to micromolar concentrations. Simultaneous determination of the enantiomeric composition of two analytes present in the same sample is also possible. We applied the click sensor **3** to scalemic mixtures of 1-phenylethylamine, **8**, and *N*-methyl-1-phenylethylamine, **17**, following the general protocol described above. The chiroptical analysis of 10 samples containing these representative amines in drastically varying *ee*’s proved sufficiently accurate for HTS purposes with errors ranging from 0.6 to 7.6% *ee* (Supplementary Table 3 and Supplementary Figures 81–84).

**Table 1:** UV/CD sensing of samples of **10** with varying concentrations and *ee*’s using **3**

Entry	Sample composition			Ratiometric sensing				
	Abs. config.	Conc. (μM)	% *ee*	Abs. config.^a^	Conc. (μM)^b^	% *ee* (257 nm)^c^	% *ee* (355 nm)^c^	% *ee* (averaged)
1	*R*	4.00	25.0	*R*	4.34	23.4	24.7	24.0
2	*R*	2.25	55.5	*R*	2.01	56.0	56.9	56.5
3	*S*	5.00	50.0	*S*	4.59	54.7	54.1	54.4
4	*R*	9.20	8.0	*R*	9.29	11.9	11.1	11.5
5	*S*	2.50	33.3	*S*	2.70	36.6	35.5	36.0
6	*R*	7.00	42.8	*R*	6.55	47.1	46.6	46.8
7	*S*	8.00	37.5	*S*	7.78	43.5	41.7	42.6
8	*S*	9.75	79.0	*S*	9.54	81.3	84.7	83.0
9	*S*	1.25	60.0	*S*	1.14	56.5	52.5	54.5

^a^Based on the sign of the CD response and comparison to a reference sample.

^b^Based on the UV response of the sensor.

^c^Based on the amplitude of the CD response

### Asymmetric reaction analysis

Finally, the chirality sensing method with chlorocoumarin **3** was applied to asymmetric reaction analysis (Table 2 and Supplementary Figures 75–80). We chose the iridium catalyzed hydrogenation of the *N*-methyl imine **48** to the secondary amine **17** for this purpose^36–40^. Several ligands **49**–**53** and catalyst loadings were varied to determine the value of chiroptical sensing and to compare it with traditional NMR/chiral high-performance liquid chromatography (HPLC) analysis. The inherent ruggedness of our click chemistry sensing approach together with the wide solvent compatibility allowed us to simply take 200 μL aliquots from the methanolic reaction mixtures for direct UV/CD analysis. Based on a conservative estimate, the analysis time per sample was 60 min and 6 mL of solvent waste for diluting the samples were generated. The vast majority of the analysis time is required for the reaction of the amine product with the probe. If necessary this can be accelerated at higher temperatures, however, one can easily conduct hundreds of these experiments in parallel using multi-well plate technology. In such a high-throughput screening (HTS) scenario, the analysis of hundreds of reaction mixtures would still take approximately 1 hour.

**Table 2:** Analysis of the asymmetric hydrogenation of *N*-methyl-1-phenylethan-1-imine


Entry	Reaction conditions			Traditional analysis^a^			Chiroptical sensing^b^		
	Ligand	Cat. load. (mol%)	Time (h)	Abs. config.	% *ee*	Conv.	Abs. Config.	% *ee*	Conv.
1	**49**	5.00	18	*S*	55.8	99.9	*S*	59.8	96.0
2	**50**	5.00	18	*R*	16.3	99.9	*R*	14.3	99.9
3	**51**	5.00	18	*R*	31.0	99.9	*R*	32.2	99.1
4	**52**	5.00	18	*R*	31.4	99.9	*R*	25.8	98.0
5	**53**	5.00	18	*S*	16.3	92.0	*S*	14.2	96.3
6	**49**	2.50	1	*S*	46.2	51.1	*S*	47.8	53.9
7	**49**	3.25	1	*S*	57.3	63.3	*S*	54.5	68.4

^a^The enantiomeric excess and conversion were determined by chiral HPLC and ^1^H NMR.

^b^The enantiomeric excess and conversion were determined by CD and UV sensing at 376 and 392 nm, respectively. *Cat. load.* catalyst loading, *Conv.* conversion

We developed a chiral HPLC method with Boc-protected **17** to verify the results from our sensing assay. The traditional NMR and chiral HPLC analysis of the enantioselective imine hydrogenation required more than 7 h and 540 mL of solvent waste were accumulated, which can be mostly attributed to the formation and purification of the Boc-protected derivative **54**. Overall, the results obtained by both methods are in good agreement. For example, the reaction with 5 mol% of the Phox ligand derived Ir catalyst gave (*S*)-**17** in 55.8% *ee* and quantitative yield according to NMR and HPLC analysis which compares well to the 59.8% *ee* and 96.0% yield determined by sensing (entry 1). The error margins of the chiroptical sensing are fairly small and acceptable, in particular if one would apply the sensing assay to HTS of hundreds of samples. The minimization of time and chemical waste compared to traditional methods underscores the efficiency, practicality, cost and environmental sustainability advantages of chiroptical sensing with the coumarin **3**.

In summary, we have developed a rugged click chemistry probe that allows comprehensive chirality sensing of a large variety of aliphatic and aromatic primary and secondary amines, amino alcohols, alcohols, and amino acids without the common shortcomings encountered with assays based on dynamic covalent chemistry, metal coordination, and supramolecular assemblies. The fast and irreversible substrate binding with readily available 4-halocoumarin sensors has several attractive features, including wide application spectrum, distinct chiroptical signaling at high wavelengths, operational simplicity, elimination of by-product formation or complex dynamic equilibria, excellent solvent compatibility, and tolerance of air and water. The usefulness and practicality of this approach were demonstrated with the chiroptical sensing of the absolute configuration, concentration, and *ee* of several samples of 2-(2-naphthyl)ethylamine and by the direct analysis of crude reaction mixtures generated by iridium catalyzed asymmetric hydrogenation of a prochiral *N*-methyl imine to *N*-methyl 1-phenylethylamine, a challenging task for currently available sensing methods. Compared to existing methodology, the practicality, robustness and scope of comprehensive click chirality sensing with 4-halocoumarin conjugate acceptors are remarkable. This strategy is uniquely effective and addresses increasingly important time efficiency and sustainability aspects by enabling reaction scale miniaturization and adaption to HTE, i.e., multiwell plate CD/UV readers, which altogether streamline asymmetric reaction development and reduce labor, cost, energy consumption and waste production.

## Methods

### General CD sensing procedure

To test the general utility of probe **3** as chirality probe, CD spectra of the sensing experiments with chiral amines **8**–**19**, amino alcohols **20**–**31**, alcohols **32**–**33**, amino acids **34**–**46** and were obtained. A solution of probe **3** (5.0 mM), a chiral amine or amino alcohol (5.0 mM) and Et_3_N (5.0 mM) in 2.0 mL of chloroform was stirred for 1 h and subjected to CD analysis after dilution to 0.24 mM unless otherwise noted. For the amino acid sensing, a solution of **3** (5.0 mM), amino acid (5.0 mM) and K_2_CO_3_ (10.0 mM) in 2.0 mL of acetonitrile–water (4:1) was stirred for 1 h and treated as described above. For alcohol sensing, a solution of **3** (10.0 mM), an alcohol (10.0 mM) and LiO*t-*Bu (20 mM) in 2.0 mL of tetrahydrofuran was stirred for 2 h prior to CD analysis. The CD spectra were collected with a standard sensitivity of 100 mdeg, a data pitch of 0.5 nm, a bandwidth of 1 nm, in a continuous scanning mode with a scanning speed of 500 nm/min and a response of 1 s, using a quartz cuvette (1 cm path length). The data were baseline corrected and smoothed using a binomial equation.

### Comprehensive chirality sensing

Probe **3** (10.0 mM) and (*S*)-1-phenylethylamine, **10**, in varying concentrations (0, 1, 2, 3, 4, 5, 6, 7, 8, 9, and 10.0 mM) were dissolved in the presence of Et_3_N (10.0 mM) in 2.0 mL of chloroform. To 10 μL of this solution, chloroform (2.0 mL) was added and the mixture was subjected to UV analysis. The UV absorbance at 355 and 265 nm increased as the concentration of **10** changed from 0 to 10 μM. Plotting and curve fitting of the UV absorbance change at 265 nm relative to 309 nm against the concentration (mM) of **10** showed a linear correlation (Fig. 3). An *ee* calibration curve was constructed using samples containing 1-(2-naphthyl)ethylamine, **10**, with varying enantiomeric composition. Probe **3** (10.0 mM) and **10** (5.0 mM) with varying *ee*’s (+100, +80, +60, +40, +20, 0, −20, −40, −60, −80, and −100%) were dissolved in the presence of Et_3_N (10.0 mM) in 2.0 mL of chloroform. After 1 h, CD analysis was carried out by diluting 25 μL of the reaction mixture with chloroform (2.0 mL). The CD amplitudes at 355 and 257 nm were plotted against the sample enantiomeric excess showing a linear correrlation (Fig. 3). Nine scalemic samples of **10** at varying concentrations in chloroform were prepared and subjected to simultaneous analysis of the concentration, enantiomeric excess and absolute configuration using probe **3**. First, a UV spectrum was obtained as described above and the concentration was calculated using regression analysis. Then, a CD spectrum was obtained as described above. The relevant intensities were used with linear regression equations to determine the enantiomeric excess. The absolute configuration was determined using the sign of the CD effects (Table 1).

### Asymmetric reaction analysis

Bis(1,5-cyclooctadiene)diiridium(I) dichloride ([Ir(cod)Cl]_2_) (12.4 mg, 0.02 mmol) was added to the ligand, **49**–**53**, (0.04 mmol) in dichloromethane and stirred for 30 min. *N*-Methyl-1-phenylethan-1-imine, **48**, (100 mg, 0.75 mmol) and the preformed metal-ligand complex (0.04 mmol) were then combined in dichloromethane:methanol (8:1) (9 mL) and the mixture was stirred under 15 bar H_2_ pressure overnight. To 200 μL of the crude reaction mixture, 4-chloro-3-nitrocoumarin, **3**, (10.0 mM), and Et_3_N (10.0 mM) were added in 2.0 mL of chloroform and stirred for 1 h. Then, 40 μL of this solution were diluted with chloroform (2.0 mL) and subjected to CD analysis to determine the absolute configuration based on the sign of the Cotton effect and the enantiomeric excess based on the CD amplitude. Another aliquot of 10 μL of the 200 μL solution was diluted with chloroform (2.0 mL) and subjected to UV analysis to determine the conversion of **48** to **17**. The chiroptical analysis was performed in analogy to the CCS experiments (see SI for details). For NMR/HPLC analysis, a 200 μL portion of the crude reaction mixture was dissolved in CDCl_3_ and analyzed by ^1^H NMR spectroscopy do determine the conversion. Another 200 μL portion of the crude reaction mixture was filtered through a cotton plug and di-*tert*-butyl dicarbonate was added to the filtrate. Due to the presence of methanol in the reaction mixture, di-*tert*-butyl dicarbonate was used in excess (3 equivalents) and the reaction was allowed to run for 5 h. Then the reaction mixture was concentrated and purified via flash column chromatography on silica using 10–40% dichloromethane in hexanes to afford *N*-Boc-*N*-methyl-1-phenylethylamine. The enantiomeric excess of *N*-Boc-*N*-methyl-1-phenylethylamine was determined by chiral HPLC using *S*,*S*-Whelk-O 1 as chiral stationary phase unless otherwise noted. Mobile phase: hexanes IPA = 99:1, flow rate = 1.0 mL/min, UV = 214 nm, *t*_R_ = 8.6 min (major) and *t*_R_ = 9.6 min (minor).

## Supplementary information

Supplementary Information

## Data Availability

The data that support the findings of this study are available from the corresponding author upon request. The X-ray crystallographic coordinates for 4-chlorocoumarin, **1**, 4-bromocoumarin, **2**, 4-iodo-nitrocoumarin, **5**, (*S*)-3-nitro-4-((1-phenylethyl)amino)coumarin, **7**, and (*R*)-3-nitro-4-(*N*,α-dimethylbenzyl)amino)coumarin, **55**, reported in this study have been deposited at the Cambridge Crystallographic Data Centre (CCDC), under deposition numbers 1853449, 1853447, 1853450, and 1853451. These data can be obtained free of charge from the Cambridge Crystallographic Data Centre via www.ccdc.cam.ac.uk/data_request/cif.
