# Supplementary material for: Click chemistry enables quantitative chiroptical sensing of chiral compounds in protic media and complex mixtures
Source: Nat Commun. 2018 Dec 14;9:5323. doi: 10.1038/s41467-018-07695-9 (PMC6294054; doi:10.1038/s41467-018-07695-9)
Supplement: Supplementary file 1 — Supplementary Information [file 41467_2018_7695_MOESM1_ESM.pdf]

Click chemistry enables quantitative chiroptical sensing of chiral compounds in protic media and complex mixtures

Thanzeel *et al.*

Supplementary Information

Supplementary Methods

All reagents and solvents were commercially available and used without further purification. Reactions were carried out under inert and anhydrous conditions. Flash chromatography was performed on silica gel, particle size 40-63 μm. ^{1}H NMR and ^{13}C NMR spectra were obtained at 400 MHz and 100 MHz, respectively, using deuterated acetonitrile and chloroform as solvents. Chemical shifts were reported in ppm relative to TMS or to the solvent peak.

Synthesis and characterization of probes and selected sensing products

4-Chlorocoumarin (1)

To a mixture of 4-hydroxycoumarin (250.0 mg, 1.54 mmol) and $POCl_3$ (5.0 mL), Et_3N (322.4 μL, 2.31 mmol) was added slowly over a period of 5-10 minutes and then the mixture was heated under reflux for 12 hours. After the reaction was completed, the mixture was quenched by pouring it slowly onto ice-cold water. The crude product was extracted with dichloromethane. The combined organic layers were washed with water, brine and dried over $MgSO_4$ and concentrated *in vacuo*. Purification by flash column chromatography on silica gel (4% ethyl acetate in hexanes) afforded 201.7 mg (1.12 mmol, 73%) of a white solid. ^{1}H NMR (400 MHz, $CDCl_3$): δ = 7.87 (m, 1H), 7.62 (ddd, *J* = 8.7, 7.3, 1.5 Hz, 1H), 7.42 – 7.34 (m, 2H), 6.61 (s, 1H). ^{13}C NMR (100 MHz, $CDCl_3$): δ = 160.0, 153.0, 149.6, 133.3, 125.5, 124.8, 118.0, 117.0, 115.5. Anal. Calcd. for $C_9H_5ClO_2$: C, 59.86; H, 2.79. Found: C, 59.62; H, 2.91.

4-Bromocoumarin (2)

A mixture of 4-hydroxycoumarin (250.0 mg, 1.54 mmol), TBAB (575.9 mg, 1.78 mmol) and P_4O_{10} (524.6 mg, 3.69 mmol) in toluene was stirred at 90-95 °C and the reaction was monitored by GC-MS. After 2 hours, the mixture was allowed to cool to room temperature and washed with water, sat. $NaHCO_3$ and extracted with dichloromethane. The combined organic layers were dried over $MgSO_4$ and concentrated *in vacuo*. Purification by flash column chromatography on silica gel (4% ethyl acetate in hexanes) afforded 204.2 mg (0.91 mmol, 59%) of a white solid. ^{1}H NMR (400 MHz, $CDCl_3$): δ = 7.84 (dd, *J* = 8.0, 1.5 Hz, 1H), 7.60 (ddd, *J* = 8.7, 7.4, 1.5 Hz, 1H), 7.41 – 7.29 (m, 2H), 6.86 (s, 1H). ^{13}C NMR (100 MHz, $CDCl_3$): δ = 158.6, 152.5, 141.4, 133.2, 128.0, 124.9, 119.6, 118.9, 117.0. Anal. Calcd. for $C_9H_5BrO_2$: C, 48.04; H, 2.24. Found: C, 48.00; H, 2.25.

4-Bromo-3-nitrocoumarin (4)

A mixture of 4-hydroxy-3-nitrocoumarin (250.0 mg, 1.21 mmol), tetra-*n*-butylammonium bromide (451.3 mg, 1.40 mmol) and P_4O_{10} (340.7 mg, 2.89 mmol) in toluene was stirred at 90-95 °C. The solution was allowed to cool to room temperature, washed with water and sat. $NaHCO_3$ and extracted with dichloromethane. The combined organic layers were dried over $MgSO_4$ and concentrated *in vacuo*. Purification by flash column chromatography on silica gel (10% ethyl acetate in hexanes) afforded 194.4 mg (0.72 mmol, 60%) of a brown solid. ^{1}H NMR (400 MHz, $CDCl_3$): δ = 7.98 (dd, *J* = 8.1, 1.6 Hz, 1H), 7.76 (ddd, *J* = 8.6, 7.3, 1.5 Hz, 1H), 7.51 (ddd, *J* = 8.3, 7.4, 1.2 Hz, 1H), 7.44 (dd, *J* = 8.4, 1.1 Hz, 1H). ^{13}C NMR (100 MHz, $CDCl_3$): δ = 151.6, 151.3, 135.3, 133.7, 129.8, 126.4, 117.4, 117.2. Anal. Calcd. for $C_9H_4BrNO_4$: C, 40.03; H, 1.49; N, 5.19. Found: C, 40.02; H, 1.71; N, 5.22.

4-Iodo-3-nitrocoumarin (5)

A mixture of 4-chloro-3-nitrocoumarin (**3**) (100.0 mg, 0.44 mmol) and NaI (263.7 mg, 1.76 mmol) was heated under reflux in acetonitrile. The reaction was monitored by GC-MS and full conversion

was observed after 18 hours. After cooling to room temperature, the reaction mixture was washed with water, $NaHCO_3$ and extracted with dichloromethane. The combined organic layers were dried over $MgSO_4$ and concentrated *in vacuo* to give 140.8 mg (0.44 mmol, 99%) of a yellow solid. ^{1}H NMR (400 MHz, $CDCl_3$): δ = 7.84 (dd, *J* = 8.2, 1.4 Hz, 1H), 7.72 (ddd, *J* = 8.0, 7.6, 1.4 Hz, 1H), 7.47 (ddd, *J* = 7.8, 7.6, 1.0 Hz, 1H), 7.38 (dd, *J* = 8.4, 0.8 Hz, 1H). ^{13}C NMR (100 MHz, $CDCl_3$): δ = 150.7, 150.6, 135.0, 134.6, 126.6, 119.4, 117.4, 113.8. Anal. Calcd. for $C_9H_4INO_4$: C, 34.10; H, 1.27; N, 4.42. Found: C, 34.11; H, 1.39; N, 4.33.

(*S*)-3-Nitro-4-((1-phenylethyl)amino)coumarin (7)

A mixture of 4-chloro-3-nitrocoumarin (**3**) (100.0 mg, 0.44 mmol), (*S*)-1-phenylethylamine (**8**) (57.2 µL, 53.7 mg) and Et_3N (61.7 µL, 0.44 mmol) was stirred in chloroform (3.0 mL). After the reaction was completed, the reaction mixture was concentrated *in vacuo*. Purification by flash chromatography (14% ethyl acetate in hexanes) afforded 122.7 mg (90%, 0.40 mmol) of a yellow solid. ^{1}H NMR (400 MHz, $CDCl_3$): δ = 10.58 (s, 1H), 7.78 (dd, *J* = 8.3, 1.4 Hz, 1H), 7.62 (ddd, *J* = 8.6, 7.3, 1.4 Hz, 1H), 7.50 – 7.42 (m, 2H), 7.41 – 7.35 (m, 3H), 7.32 (m, 1H), 7.17 (ddd, *J* = 8.4, 7.2, 1.3 Hz, 1H), 5.38 (m, 1H), 1.78 (d, *J* = 6.6 Hz, 3H). ^{13}C NMR (100 MHz, $CDCl_3$): δ = 154.4, 153.6, 152.7, 141.5, 135.2, 129.6, 128.5, 127.2, 125.3, 124.2, 118.3, 115.9, 112.8, 57.9, 26.3. Anal. Calcd. for $C_{17}H_{14}N_2O_4$: C, 65.80; H, 4.55; N, 9.03. Found: C, 65.87; H, 4.78; N, 8.81.

4-(((1*S*,2*R*)-2-Hydroxy-2,3-dihydro-1H-inden-1-yl)amino)-3-nitrocoumarin

A mixture of 4-chloro-3-nitrocoumarin (**3**) (100.0 mg, 0.44 mmol), (1*S*,2*R*)-*cis*-1-amino-2-indanol (**22**) (66.1 mg, 0.44 mmol) and Et_3N (61.7 µL, 0.44 mmol) was stirred in chloroform (3.0 mL). After the reaction was completed, the mixture was concentrated *in vacuo*. Purification by flash chromatography (50% ethyl acetate in hexanes) afforded 133.1 mg (89%, 0.39 mmol) of a brown solid. ^{1}H NMR (400 MHz, $(CD_3)_2SO$): δ = 8.28 (d, *J* = 8.3 Hz, 1H), 7.78 (m, 1H), 7.46 (m, 1H), 7.44 – 7.38 (m, 2H), 7.37 – 7.24 (m, 3H), 5.83 (s, 1H), 5.44 (bs, 1H), 4.58 (q, *J* = 4.4 Hz, 1H), 3.18 (dd, *J* = 16.4, 4.7 Hz, 1H), 2.92 (d, *J* = 16.4 Hz, 1H). ^{13}C NMR (100 MHz, $(CD_3)_2SO$): δ = 154.8, 152.1, 141.8, 139.9, 135.2, 128.8, 127.2, 126.5, 125.8, 125.2, 124.9, 118.1, 116.0, 113.9, 73.4, 63.8, 55.3. Anal. Calcd. for $C_{18}H_{14}N_2O_5$: C, 63.90; H, 4.17; N, 8.28. Found: C, 63.62; H, 4.25; N, 8.11.

(*R*)-3-Nitro-4-(*N*,α-dimethylbenzyl)amino)coumarin

A mixture of 4-chloro-3-nitrocoumarin (**3**) (45.0 mg, 0.20 mmol), (*R*)-*N*-methyl-1-phenylethylamine (**17**) (29.2 µL, 0.24 mmol) and Et_3N (33.5 µL, 0.24 mmol) was stirred in chloroform (1.0 mL). After the reaction was completed, the reaction mixture was concentrated *in vacuo*. Purification by flash chromatography (30% ethyl acetate in hexanes) afforded 62 mg (96%, 0.19 mmol) of a yellow solid. ^{1}H NMR (400 MHz, $CDCl_3$): δ = 7.83 (dd, *J* = 8.2, 1.5 Hz, 1H), 7.60 (ddd, *J* = 8.6, 7.2, 1.5 Hz, 1H), 7.49 – 7.42 (m, 2H), 7.42 – 7.35 (m, 4H), 7.25 – 7.19 (m, 1H), 5.32 (q, *J* = 6.9 Hz, 1H), 2.82 (s, 3H), 1.77 (d, J = 6.8 Hz, 3H). ^{13}C NMR (100 MHz, $CDCl_3$): δ = 155.7, 152.9, 152.5, 138.5, 133.4, 129.0, 128.4, 128.2, 126.9, 126.2, 124.6, 118.3, 116.6, 62.3, 33.2, 17.9. Anal. Calcd. for $C_{18}H_{16}N_2O_4$: C, 66.66; H, 4.97; N, 8.64. Found: C, 66.65; H, 5.17; N, 8.60.

Probe development and optimization studies

Initially, reactions were performed with 5.0 mM (*S*)-1-phenylethylamine (**8**) concentrations as described below to identify a probe with superior chiroptical properties. The CD spectra of the

diluted solutions (0.24 mM) were collected with a standard sensitivity of 100 mdeg, a data pitch of 0.5 nm, a bandwidth of 1 nm, in a continuous scanning mode with a scanning speed of 500 nm/min and a response of 1 s, using a quartz cuvette (1 cm path length). The data were baseline corrected and smoothed using a binomial equation. UV spectra were collected with an average scanning time of 0.0125 s, a data interval of 5.00 nm and a scan rate of 400 nm/s.

CD analysis with different derivatives of coumarin

A solution of 4-chloro-3-nitrocoumarin (**3**) (5.0 mM), (*S*)-1-phenylethylamine (**8**) (5.0 mM) and Et_3N (5.0 mM) in 2.0 mL of chloroform was stirred for 1 hour. To 100 µL of this solution chloroform (2.0 mL) was added and the mixture was subjected to CD analysis (0.24 mM). Control experiments with (*S*)-1-phenylethylamine (**8**) in the absence of the probe did not show any CD signal at the wavelengths of interest. The analysis was repeated with 4-chlorocoumarin (**1**) and 4-bromocoumarin (**2**). No reaction occurred under the conditions described above (Supplementary Figure 8). A mixture of 4-chlorocoumarin (**1**) (9.2 mg, 0.05 mmol), (*S*)-1-phenylethylamine (**8**) (6.5 µL, 0.05 mmol) and Et_3N (7.0 µL, 0.05 mmol) was heated to 60-70 °C in $CHCl_3$ in a closed vessel for 3 hours. No reaction occurred based on ^{1}H NMR and TLC analysis.

A mixture of 4-chlorocoumarin (**1**) (9.8 mg, 0.05 mmol), (*S*)-1-phenylethylamine (**8**) (6.9 µL, 0.05 mmol) and Et_3N (7.5 µL, 0.05 mmol) was heated in acetonitrile to 120 °C in a microwave reactor (150 W). After 1 hour, the reaction mixture was concentrated in vacuo. Purification by flash chromatography (0%-5% MeOH in dichloromethane) afforded 7 mg (49%, 0.03 mmol) of a white solid. ^{1}H NMR (400 MHz, $CDCl_3$): δ = 7.59 – 7.50 (m, 2H), 7.40 – 7.27 (m, 7H), 5.37 (d, *J* = 5.6 Hz, 1H), 5.21 (s, 1H), 4.67 (m, 1H), 1.66 (d, *J* = 6.8 Hz, 3H). The CD spectrum is shown in Supplementary Figure 9.

Amine sensing using 4-chloro-3-nitrocoumarin (3)

A solution of 4-chloro-3-nitrocoumarin (**3**) (5.0 mM), (*S*)-1-phenylethylamine (**8**) (5.0 mM) and Et_3N (5.0 mM) in 2.0 mL of chloroform was stirred for 1 hour. To 100 µL of this solution, chloroform (2.0 mL) was added and the mixture was subjected to CD analysis at 0.24 mM (Supplementary Figure 10). Control experiments with (*S*)-1-phenylethylamine (**8**) in the absence of the probe did not show any CD signal at the wavelengths of interest.

Amine sensing using 4-bromo-3-nitrocoumarin (4)

A solution of 4-bromo-3-nitrocoumarin (**2**) (5.0 mM), (*S*)-1-phenylethylamine (**8**) (5.0 mM) and Et_3N (5.0 mM) in 2.0 mL of chloroform was stirred for 1 hour. To 100 µL of this solution, chloroform (2.0 mL) was added and the mixture was subjected to CD analysis at 0.24 mM (Supplementary Figure 11). Control experiments with (*S*)-1-phenylethylamine (**8**) in the absence of the probe did not show any CD signal at the wavelengths of interest.

Amine sensing using 4-iodo-3-nitrocoumarin (5)

A solution of 4-iodo-3-nitrocoumarin (**5**) (5.0 mM), (*S*)-1-phenylethylamine (**8**) (5.0 mM) and Et_3N (5.0 mM) in 2.0 mL of chloroform was stirred for 1 hour. To 100 µL of this solution, chloroform (2.0 mL) was added and the mixture was subjected to CD analysis at 0.24 mM (Supplementary Figure 12). Control experiments with (*S*)-1-phenylethylamine (**8**) in the absence of the probe did not show any CD signal at the wavelengths of interest.

Solvent and base optimization using 4-chloro-3-nitrocoumarin (3)

A solution of probe **3** (5.0 mM), (*S*)-phenylethylamine (**8**) (5.0 mM) and Et_3N (5.0 mM) in 2.0 mL of chloroform was stirred for 1 hour. To 80 μL of this solution, chloroform (2.0 mL) was added and the mixture was subjected to CD analysis (0.19 mM). The above experiment was repeated with dichloromethane, acetonitrile and toluene as solvents with TBAOH and in the absence of base (Supplementary Figures 14-16).

Supplementary mechanistic studies

The CD spectra were collected with a standard sensitivity of 100 mdeg, a data pitch of 0.5 nm, a bandwidth of 1 nm, in a continuous scanning mode with a scanning speed of 500 nm/min and a response of 1 s, using a quartz cuvette (1 cm path length). The data were baseline corrected and smoothed using a binomial equation. UV spectra were collected with an average scanning time of 0.1 s, a data interval of 1.00 nm and a scan rate of 600 nm/min.

Identification of the sensing product

(*S*)-3-Nitro-4-((1-phenylethyl)amino)coumarin (**7**) was synthesized from probe **3** and (*S*)-phenylethylamine (**8**) as described above. Comparison of the CD spectrum of the isolated product with the CD spectrum obtained from the reaction mixture showed that they were identical. CD measurements were taken at 0.24 mM concentrations (Supplementary Figure 17).

4-(((1*S*,2*R*)-2-Hydroxy-2,3-dihydro-1H-inden-1-yl)amino)-3-nitrocoumarin was synthesized from probe **3** and (1*S*,2*R*)-*cis*-1-amino-2-indanol (**22**) as described above. Comparison of the CD spectrum of the isolated product with the CD spectrum obtained from the reaction mixture, showed that they were identical. CD measurements were taken at 0.24 mM concentrations (Supplementary Figure 18).

(*R*)-3-Nitro-4- (*N*,α-dimethylbenzyl) amino)coumarin was synthesized from probe **3** and (*R*)-*N*-methyl-1-phenylethylamine (**17**) as described above. Comparison of the CD spectrum of the isolated product with the CD spectrum obtained from the reaction mixture showed that they were identical. CD measurements were taken at 0.10 mM concentrations (Supplementary Figure 19).

Reaction analysis

The reaction between (*S*)-phenylethylamine (**8**) (5.0 mM) and probe **3** (5.0 mM) in the presence of Et_3N (5.0 mM) in 0.80 mL of $CDCl_3$ was monitored by ^1H NMR. The reaction was complete within 15 minutes under these conditions (Supplementary Figures 20 and 21).

Reaction time

The capture of (*S*)-1-phenylethylamine (**8**) (1.25 mM) by probe **3** (1.25 mM) in the presence of Et_3N (1.25 mM) in 6.0 mL of chloroform was monitored using UV-Vis spectroscopy. Measurements were taken at 18 μM concentration, after dilution of 30 μL reaction mixture aliquots with 2.0 mL of chloroform. The reaction was complete in 40 minutes under these conditions (Supplementary Figures 22 and 23).

The capture of (*S*)-1-phenylethylamine (**8**) (1.25 mM) by probe **4** (1.25 mM) in the presence of Et_3N (1.25 mM) in 6.0 mL of chloroform was monitored using UV-Vis spectroscopy. Measurements were taken at 18 μM concentration, after dilution of 30 μL reaction mixture aliquots with 2.0 mL of chloroform. The reaction was complete after 40 minutes under these conditions (Supplementary Figures 24 and 25).

The capture of (*S*)-1-phenylethylamine (**8**) (1.25 mM) by probe **5** (1.25 mM) in the presence of Et_3N (1.25 mM) in 6.0 mL of chloroform was monitored using UV-Vis spectroscopy. Measurements were taken at 18 µM concentration, after dilution of 30 µL reaction mixture aliquots with 2.0 mL of chloroform. The reaction was complete in less than 100 minutes under these conditions (Supplementary Figures 26 and 27).

Sensing in protic solvents

A solution of probe **3** (5.0 mM), (*S*)-phenylethylamine (**8**) (5.0 mM) and Et_3N (5.0 mM) in 2.0 mL of chloroform was stirred for 1 hour. To 100 µL of this solution, solvent (2.0 mL) was added and the mixture was subjected to CD analysis (0.24 mM). CD spectra were collected in chloroform, methanol and chloroform-methanol (1:1) mixture (Supplementary Figure 28).

Sensing scope

To test the utility of probe **3** as chirality chemosensor, CD spectra of the sensing experiments with chiral amines **8-19**, chiral amino alcohols **20-31**, chiral alcohols **32-33**, chiral amino acids **34-46** and were obtained. The CD spectra were collected with a standard sensitivity of 100 mdeg, a data pitch of 0.5 nm, a bandwidth of 1 nm, in a continuous scanning mode with a scanning speed of 500 nm/min and a response of 1 s, using a quartz cuvette (1 cm path length). The data were baseline corrected and smoothed using a binomial equation.

Amines

A solution of probe **3** (5.0 mM), chiral amines (**8-19**) (5.0 mM) and Et_3N (5.0 mM) in 2.0 mL of chloroform was stirred for 1 hour and subjected to CD and UV analysis (Supplementary Figures 29-41).

Amino alcohols

A solution of probe **3** (5.0 mM), chiral amino alcohols (5.0 mM) and Et_3N (5.0 mM) in 2.0 mL of chloroform was stirred for 1 hour and subjected to CD and UV analysis (Supplementary Figures 42-53).

Alcohols

A solution of probe **3** (10.0 mM), chiral alcohols (10.0 mM) and LiO^tBu (20 mM) in 2.0 mL of tetrahydrofuran was stirred for 2 hours and subjected to CD and UV analysis (Supplementary Figures 54 and 55).

Amino acids

A solution of probe **3** (5.0 mM), chiral amino acids (5.0 mM) and K_2CO_3 (10.0 mM) in 2.0 mL of acetonitrile-water (4:1) mixture was stirred for 1 hour and subjected to CD and UV analysis (Supplementary Figures 56-69).

Quantitative sensing: absolute configuration, enantiomeric excess and total concentration

The CD spectra were collected with a standard sensitivity of 100 mdeg, a data pitch of 0.5 nm, a bandwidth of 1 nm, in a continuous scanning mode with a scanning speed of 500 nm/min and a response of 1 s, using a quartz cuvette (1 cm path length). The data were baseline corrected and smoothed using a binomial equation. UV spectra were collected with an average scanning time of 0.0125 s, a data interval of 5.00 nm and a scan rate of 400 nm/s.

Determination of the concentration of (*S*)-1-(2-naphthyl)ethylamine using probe 1

The change in the UV absorbance of probe **3** upon (*S*)-1-(2-naphthyl)ethylamine (**10**) sensing was analyzed. Probe **3** (10.0 mM) and **10** in varying concentrations (0, 1, 2, 3, 4, 5, 6, 7, 8, 9 and 10.0

mM) were dissolved in the presence of Et_3N (10.0 mM) in 2.0 mL of chloroform. To 10 μL of this solution, chloroform (2.0 mL) was added and the mixture was subjected to UV analysis. The UV absorbance at 355 nm and 265 nm increased as the concentration of (*S*)-1-(2-naphthyl)ethylamine (**10**) changed from 0 to 10 μM. Plotting and curve fitting of the UV absorbance change at 265 nm relative to 309 nm against the concentration (mM) of (*S*)-1-(2-naphthyl)ethylamine (**10**) showed a linear relationship (Supplementary Figures 70 and 71).

Determination of the enantiomeric excess of 1-(2-naphthyl)ethylamine (10) using probe 3
A calibration curve was constructed using samples containing 1-(2-naphthyl)ethylamine (**10**) with varying enantiomeric composition. Probe **3** (10.0 mM) and 1-(2-naphthyl)ethylamine (**10**) (5.0 mM) with varying ee's (+100, +80, +60, +40, +20, 0, -20, -40, -60, -80, -100%) were dissolved in the presence of Et_3N (10.0 mM) in 2.0 mL of chloroform. After 1 hour, CD analysis was carried out by diluting 25 μL of the reaction mixture with chloroform (2.0 mL). The CD amplitudes at 355 and 257 nm were plotted against the enantiomeric excess of 1-(2-naphthyl)ethylamine (**10**) (Supplementary Figures 72 and 73).

Simultaneous ee and concentration determination

Nine scalemic samples of 1-(2-naphthyl)ethylamine (**10**) at varying concentrations in chloroform were prepared and subjected to simultaneous analysis of the concentration, enantiomeric excess and absolute configuration using probe **3**. First, a UV spectrum was obtained as described above and the concentration was calculated using regression equation (Supplementary Equation 1) below. Then, a CD spectrum was obtained as described above. The relevant intensities were used with linear regression equations (Supplementary Equations 2 and 3) to determine the enantiomeric excess. The absolute configuration was determined by comparing the sign of the Cotton effect to a reference. The results are shown in Supplementary Table 1 and Supplementary Figure 74.

Chiroptical sensing of crude reaction mixtures of the asymmetric reduction of *N*-methyl-1-phenylethan-1-imine
All commercially available reagents and solvents were used without further purification. ^1H NMR spectra were obtained at 400 MHz and ^{13}C NMR were obtained at 100 MHz. *N*-Boc protected asymmetric reduction products were purified by flash column chromatography on silica gel (particle size = 40-60 μm). The enantiomeric ratio was determined by chiral HPLC.
The CD spectra were collected with a standard sensitivity of 100 mdeg, a data pitch of 0.5 nm, a bandwidth of 1 nm, in a continuous scanning mode with a scanning speed of 500 nm/min and a response of 1 s, using a quartz cuvette (1 cm path length). The data were baseline corrected and smoothed using a binomial equation. UV spectra were collected with an average scanning time of 0.1 s, a data interval of 1.00 nm and a scan rate of 600 nm/min. Because of the UV and CD absorption of the iridium catalyst and other starting materials, ratiometric reaction analysis with the absorption at 265 nm was not possible. We therefore used calibration curves of signals above 300 nm.

UV Calibration curve of the sensing of *N*-methyl-1-phenylethylamine (17) using probe 3
The change in the UV absorbance of probe **3** upon (*S*)-*N*-methyl-1-phenylethylamine (**17**) sensing was analyzed. Probe **3** (10.0 mM) and (*S*)-*N*-methyl-1-phenylethylamine (**17**) in varying concentrations (0, 1, 2, 3, 4, 5, 6, 7, 8, 9 and 10.0 mM) were dissolved in the presence of Et_3N

(10.0 mM) in 2.0 mL of chloroform. To 10 μL of this solution, chloroform (2.0 mL) was added and the mixture was subjected to UV analysis. The UV absorbance at 392 nm increased as the concentration of (*S*)-*N*-methyl-1-phenylethylamine (**17**) changed from 0 to 10 μM. Plotting and curve fitting of the UV absorbance at 392 nm against the concentration (mM) of (*S*)-*N*-methyl-1-phenylethylamine (**17**) showed a linear relationship (Supplementary Figures 75 and 76).

Determination of the enantiomeric excess of *N*-methyl-1-phenylethylamine (17) using probe 3

A calibration curve was constructed using samples containing *N*-methyl-1-phenylethylamine (**17**) with varying enantiomeric composition. Probe **3** (10.0 mM) and *N*-Methyl-1-phenylethylamine (**17**) (5.0 mM) with varying ee's (+100, +80, +60, +40, +20, 0, -20, -40, -60, -80, -100%) were dissolved in the presence of Et_3N (10.0 mM) in 2.0 mL of chloroform. After 1 hour, CD analysis was carried out by diluting 40 μL of the reaction mixture with chloroform (2.0 mL). The CD amplitudes at 376 nm were plotted against the enantiomeric excess of (*S*)-*N*-methyl-1-phenylethylamine (**17**) (Supplementary Figures 77 and 78).

Asymmetric reduction of *N*-methyl-1-phenylethan-1-imine (48) and subsequent analysis

N-Methyl-1-phenylethan-1-imine (**48**) was synthesized via a modified literature procedure.[1] Acetophenone (1.0 g, 8.32 mmol) was added to a solution of CH_3NH_2 (33% in EtOH, 5 ml) with activated 4 Å molecular sieves (250 mg /1 mmol) and the reaction was allowed to complete without stirring. Concentration of the reaction mixture *in vacuo* afforded 1.1 g (97%, 8.2 mmol) of a colorless oil which was used without further purification in the Ir catalyzed enantioselective hydrogenation. Bis(1,5-cyclooctadiene)diiridium(I) dichloride ($[Ir(cod)Cl]_2$) (12.4 mg, 0.02 mmol) was added to the ligand (0.04 mmol) (**49-53**) in dichloromethane and stirred for 30 minutes. *N*-Methyl-1-phenylethan-1-imine (**48**) (100 mg, 0.75 mmol) and the preformed metal-ligand complex (0.04 mmol) were mixed in dichloromethane:methanol (8:1) (9 mL) and stirred overnight under 15 bar H_2 pressure.

Simultaneous ee and concentration determination of the crude reaction mixture

To 200 μL of the crude reaction mixture, 4-chloro-3-nitrocoumarin (**3**) (10.0 mM), and Et_3N (10.0 mM) were added in 2.0 mL of chloroform and stirred for 1 hour. Then, 40 μL of this solution were diluted with chloroform (2.0 mL) and subjected to CD analysis to determine the absolute configuration based on the sign of the Cotton effect and the enantiomeric excess based on the CD amplitude. Another aliquot of 10 μL of the sensing solution was diluted with chloroform (2.0 mL) and subjected to UV analysis to determine the conversion. The results were determined using Supplementary Equations 4 and 5 (Supplementary Table 2).

HPLC analysis

A portion of the crude reaction mixture was filtered through a cotton plug and di-*tert*-butyl dicarbonate was added to the filtrate. Due to the presence of methanol in the reaction mixture, di-*tert*-butyl dicarbonate was used in excess (3 equivalents) and the reaction was allowed to run for 5 hours. Then the reaction mixture was concentrated and purified via flash column chromatography on silica using 10%-40% dichloromethane in hexanes to afford a colorless oil of *N*-Boc-*N*-methyl-1-phenylethylamine. The enantiomeric excess of *N*-Boc-*N*-methyl-1-phenylethylamine was determined by chiral HPLC on an *S*,*S*-Whelk-O 1 column unless otherwise noted. Mobile phase: hexanes:IPA = 99:1, flow rate = 1.0 mL/min, UV= 214 nm, t_{R} = 8.6 min (major) and t_{R} = 9.6 min (minor) (Supplementary Figures 79 and 80). Synthesis of racemic *N*-Boc-*N*-methyl-1-phenylethylamine for HPLC development: (±)-*N*-methyl-1-phenylethylamine (0.74

mmol, 100 mg) and di-*tert*-butyl dicarbonate (0.74 mmol, 161.4 mg) were stirred in dichloromethane for 3 hours. Purification by flash column chromatography (20%-60% dichloromethane in hexanes) afforded 170 mg (98%, 0.72 mmol) of a colorless oil. ^{1}H NMR of the *N*-Boc-*N*-methyl-1-phenylethylamine (400 MHz, CD_3CN): δ = 7.36 (dd, *J* = 8.0, 6.7 Hz, 2H), 7.32-7.21 (m, 3H), 5.44-5.28 (m, 1H), 2.58 (s, 3H), 1.48 (d, *J* = 7.1 Hz, 3H), 1.45 (s, 9H).

Simultaneous determination of the enantiomeric excess of 1-phenylethylamine (8) and *N*-methyl-1-phenylethylamine (17) using probe 3

A calibration curve was constructed using samples containing 1-phenylethylamine (**8**) with varying enantiomeric composition. Probe **3** (11.3 mM) and 1-phenylethylamine (**8**) (5.0 mM) with varying ee's (+100, +80, +60, +40, +20, 0, -20, -40, -60, -80, -100%) were dissolved in the presence of Et_3N (11.3 mM) in 2.0 mL of chloroform. After 1 hour, CD analysis was carried out by diluting 30 μL of the reaction mixture with chloroform (2.0 mL). The CD amplitudes at 340 nm were plotted against the enantiomeric excess of 1-phenylethylamine (**8**) (Supplementary Figures 81 and 82).

A calibration curve was constructed using samples containing *N*-methyl-1-phenylethylamine (**17**) with varying enantiomeric composition. Probe **3** (11.3 mM) and *N*-methyl-1-phenylethylamine (**17**) (5.0 mM) with varying ee's (+100, +80, +60, +40, +20, 0, -20, -40, -60, -80, -100%) were dissolved in the presence of Et_3N (11.3 mM) in 2.0 mL of chloroform. After 1 hour, CD analysis was carried out by diluting 30 μL of the reaction mixture with chloroform (2.0 mL). The CD amplitudes at 410 nm were plotted against the enantiomeric excess of *N*-methyl-1-phenylethylamine (**17**) (Supplementary Figures 83 and 84).

Simultaneous ee determination of two chiral amines

Ten scalemic samples containing 1-phenylethylamine (**8**) (5 mM) and *N*-methyl-1-phenylethylamine (**17**) (5 mM) with varying %ee were prepared in chloroform and subjected to simultaneous analysis of the enantiomeric excess and absolute configuration using probe **3** (11.3 mM) and Et_3N (11.3 mM). CD spectra were obtained as described above and the enantiomeric excess was calculated using regression equations (Supplementary Equations 6 and 7) shown below. The absolute configuration was determined from the sign of the Cotton effect at 340 and 410 nm. The results are shown in Supplementary Table 3.

Supplementary Crystallographic Analysis

A single crystal was obtained by slow evaporation of a solution of **1** in chloroform. Single crystal X-ray analysis was performed at 100 K using a Siemens platform diffractometer with graphite monochromated Mo- Kα radiation (λ = 0.71073 Å). Data were integrated and corrected using the APEX 3 program. The structures were solved by direct methods and refined with full-matrix least square analysis using SHELX-97-2 software. Non-hydrogen atoms were refined with anisotropic displacement parameter. Crystal data: $C_9H_5ClO_2$, *M* = 180.58, prism, 0.32 x 0.27 x 0.07 mm^3, monoclinic space group, $P2_1/n$, *a* = 7.0745(13), *b* = 12.671(2), *c* = 8.9875(17) Å, *V* = 751.2(2) Å^3, *Z* = 4 (Supplementary Figure 85).

A single crystal was obtained by slow evaporation of a solution of **2** in chloroform. Single crystal X-ray analysis was performed at 100 K using a Siemens platform diffractometer with graphite monochromated Mo- Kα radiation (λ = 0.71073 Å). Data were integrated and corrected using the APEX 3 program. The structures were solved by direct methods and refined with full-matrix least

square analysis using SHELX-97-2 software. Non-hydrogen atoms were refined with anisotropic displacement parameter. Crystal data: $C_9H_5BrO_2$, *M* = 225.03, prism, 0.19 x 0.10 x 0.08 mm^3, monoclinic space group, *P2₁/n*, *a* = 7.1649(9), *b* = 12.9828(16), *c* = 9.0176(11) Å, *V* = 783.24(17) Å^3, *Z* = 4 (Supplementary Figure 86).

A single crystal was obtained by slow evaporation of a solution of **5** in chloroform. Single crystal X-ray analysis was performed at 100 K using a Siemens platform diffractometer with graphite monochromated Mo- Kα radiation (λ = 0.71073 Å). Data were integrated and corrected using the APEX 3 program. The structures were solved by direct methods and refined with full-matrix least square analysis using SHELX-97-2 software. Non-hydrogen atoms were refined with anisotropic displacement parameter. Crystal data: $C_9H_4INO_4$, *M* = 317.03, prism, 0.24 x 0.17 x 0.09 mm^3, monoclinic space group, *Cc*, *a* = 14.714(1), *b* = 8.0151(5), *c* = 9.2491(6) Å, *V* = 955.80(11) Å^3, *Z* = 4 (Supplementary Figure 87).

A single crystal was obtained by slow evaporation of a solution of **7** in 50% chloroform in hexanes. Single crystal X-ray analysis was performed at 100 K using a Siemens platform diffractometer with graphite monochromated Mo- Kα radiation (λ = 0.71073 Å). Data were integrated and corrected using the APEX 3 program. The structures were solved by direct methods and refined with full-matrix least square analysis using SHELX-97-2 software. Non-hydrogen atoms were refined with anisotropic displacement parameter. Crystal data: $C_{17}H_{14}N_2O_4$, *M* = 310.30, prism, 0.46 x 0.41 x 0.34 mm^3, triclinic space group, *P1*, *a* = 7.4195(3), *b* = 7.5034(3), *c* = 15.0658(7) Å, *V* = 716.64(5) Å^3, *Z* = 2 (Supplementary Figure 88).

A single crystal was obtained by slow evaporation of a solution of (*R*)-3-nitro-4-(*N*,α-dimethylbenzyl)amino)coumarin in 50% hexanes in ethyl acetate. Single crystal X-ray analysis was performed at 100 K using a Siemens platform diffractometer with graphite monochromated Mo- Kα radiation (λ = 0.71073 Å). Data were integrated and corrected using the APEX 3 program. The structures were solved by direct methods and refined with full-matrix least square analysis using SHELX-97-2 software. Non-hydrogen atoms were refined with anisotropic displacement parameter. Crystal data: $C_{18}H_{16}N_2O_4$, *M* = 324.33, prism, 0.22 x 0.19 x 0.09 mm^3, monoclinicspace group, *P2₁*, *a* = 6.4366(9), *b* = 6.6366(10), *c* = 18.111(3) Å, *V* = 765.5(2) Å^3, *Z* = 2 (Supplementary Figure 89).

Supplementary Figures

Supplementary Figure 1: ^{1}H NMR and ^{13}C NMR spectra of 4-chlorocoumarin (**1**) in $CDCl_3$

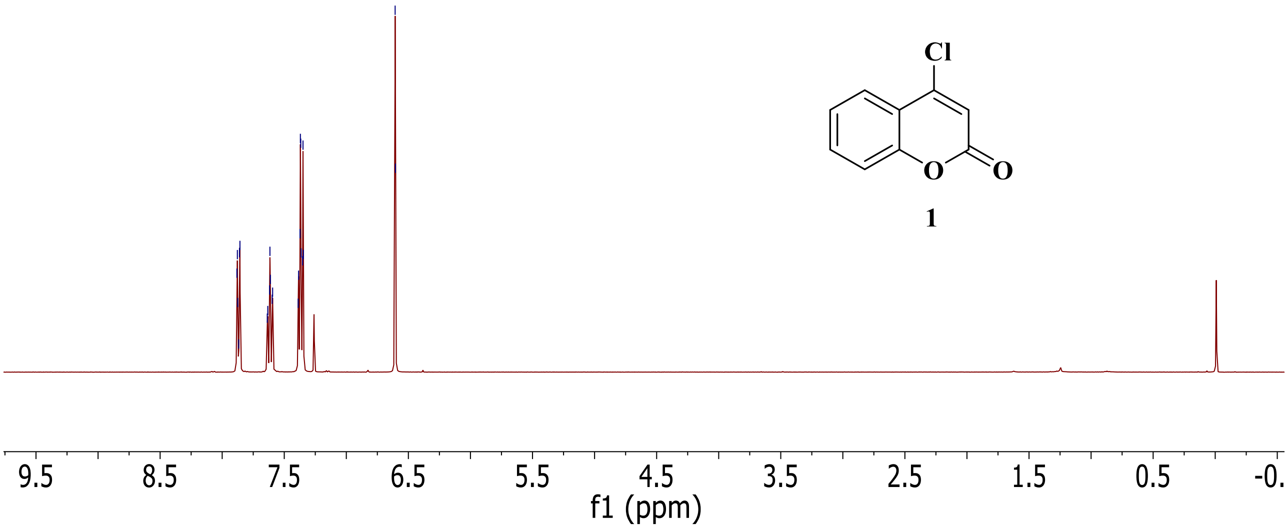

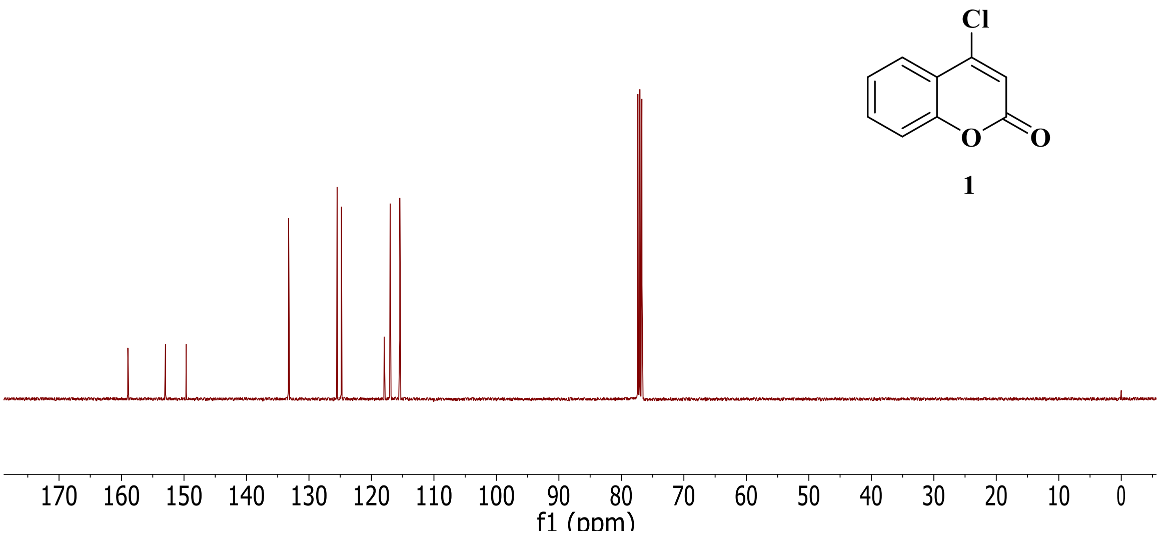

Supplementary Figure 2: ^{1}H NMR and ^{13}C NMR spectra of 4-bromocoumarin (**2**) in $CDCl_3$

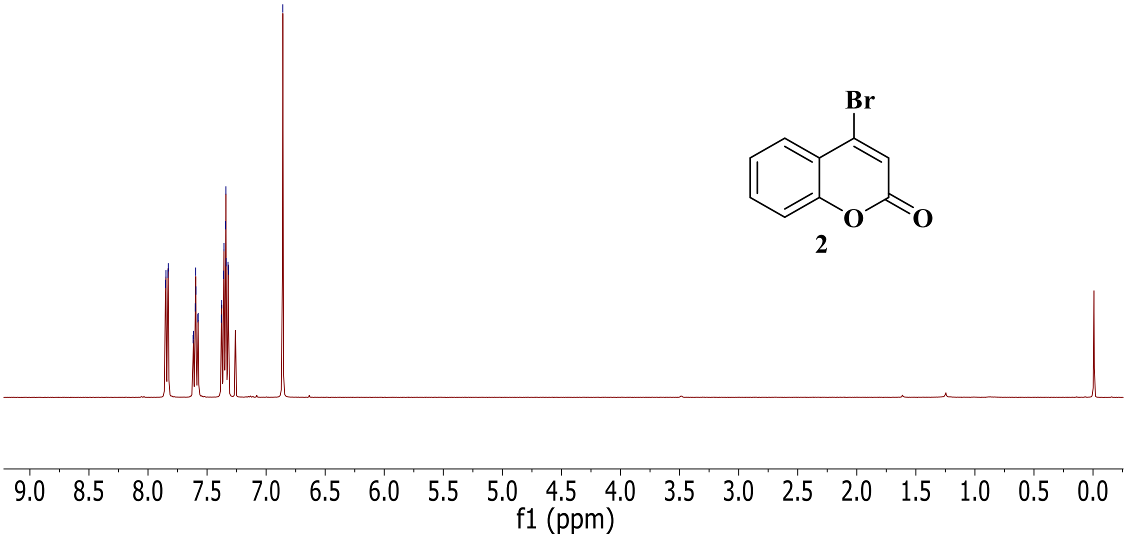

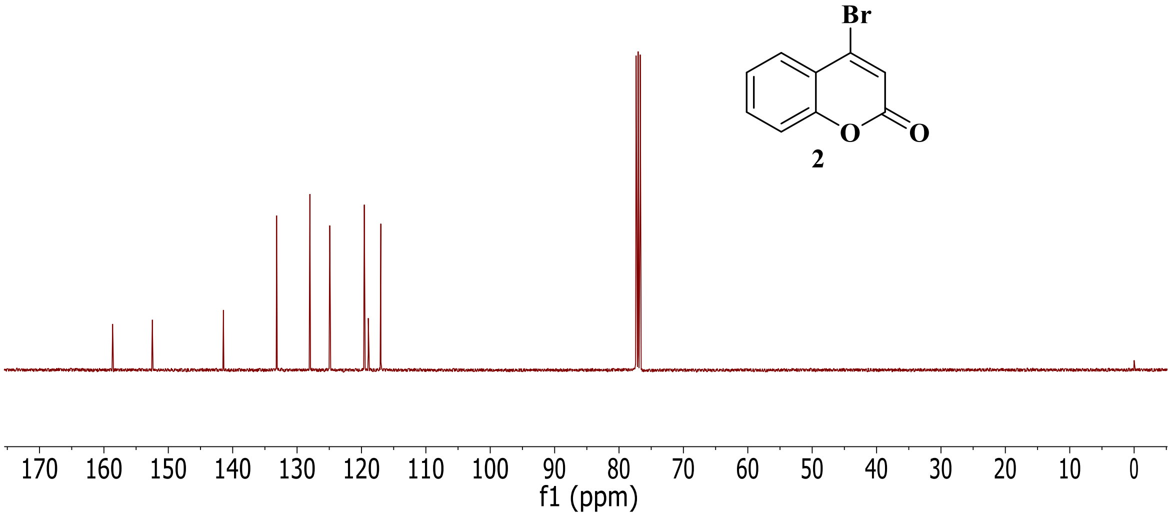

Supplementary Figure 3: ^{1}H NMR and ^{13}C NMR spectra of 4-bromo-3-nitrocoumarin (**4**) in $CDCl_3$

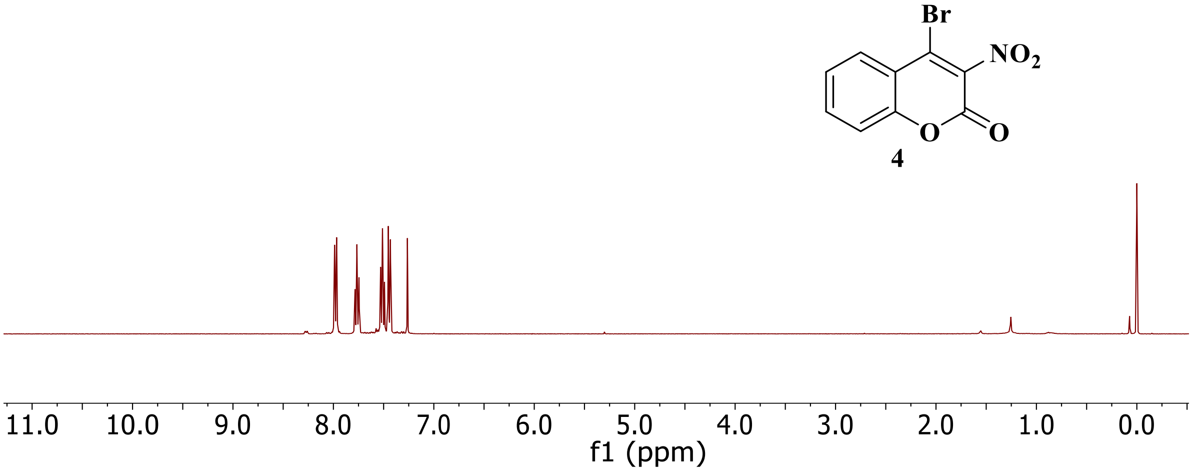

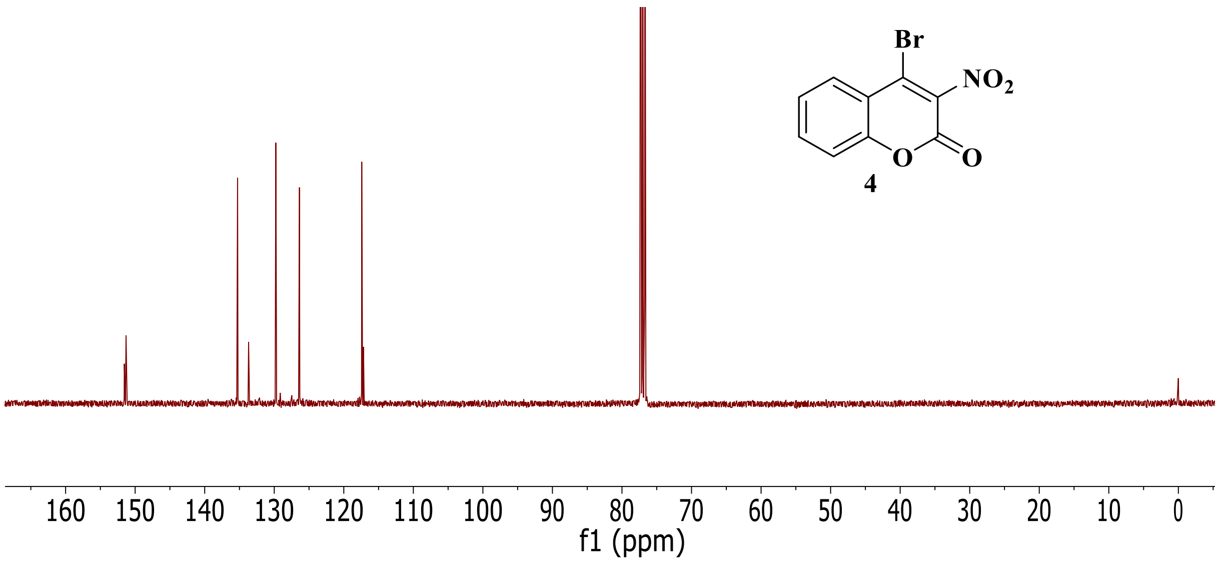

Supplementary Figure 4: ^{1}H NMR and ^{13}C NMR spectra of 4-iodo-3-nitrocoumarin (**5**) in $CDCl_3$

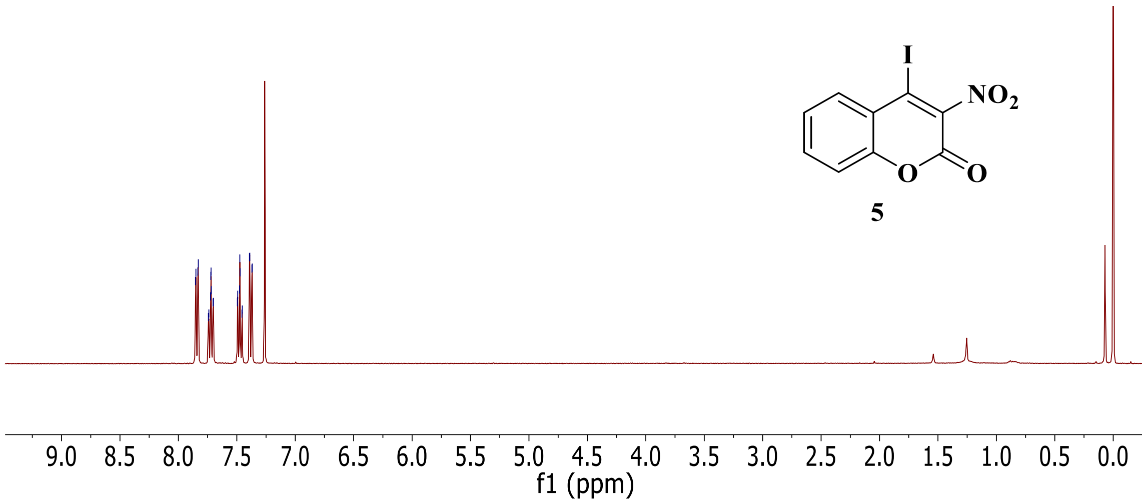

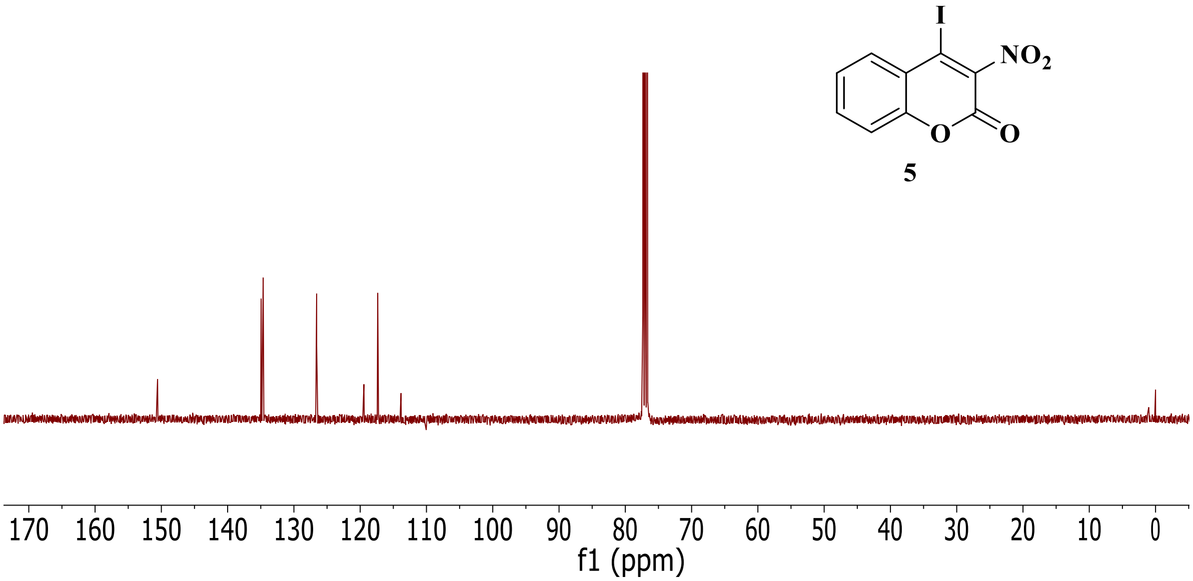

Supplementary Figure 5: ^{1}H NMR and ^{13}C NMR spectra of (*S*)-3-nitro-4-((1-phenylethyl)amino)coumarin (**7**) in $CDCl_3$

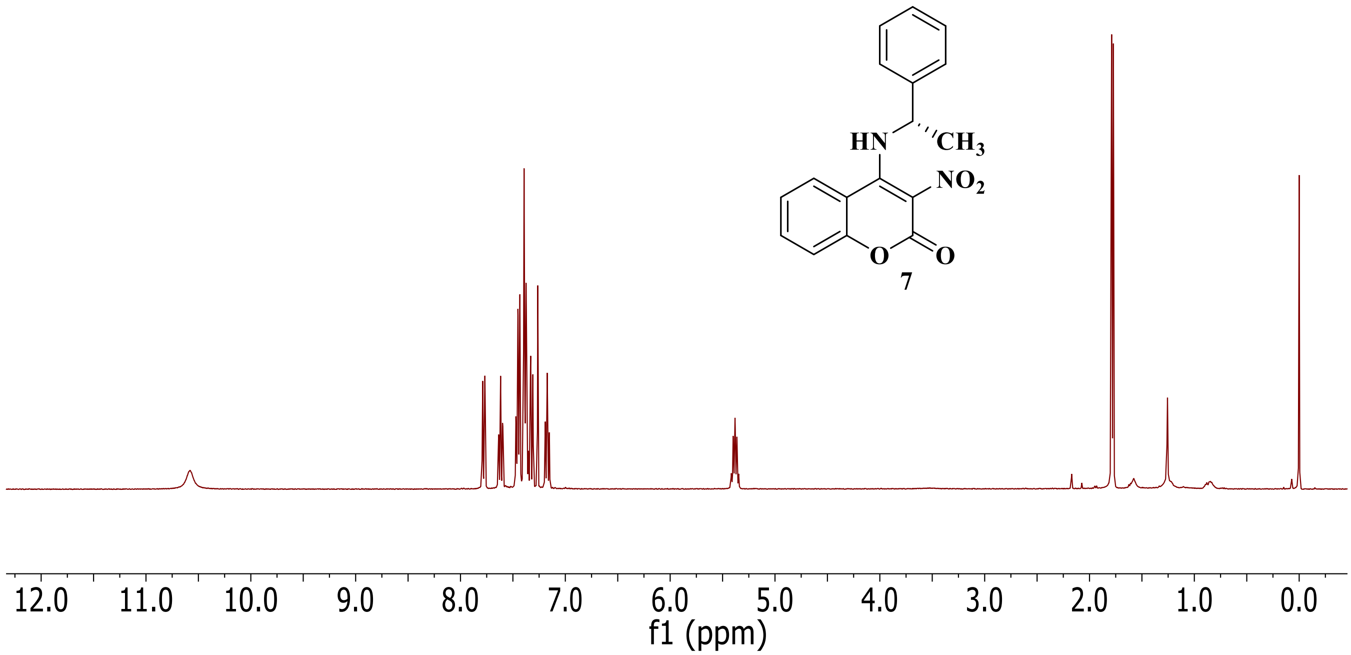

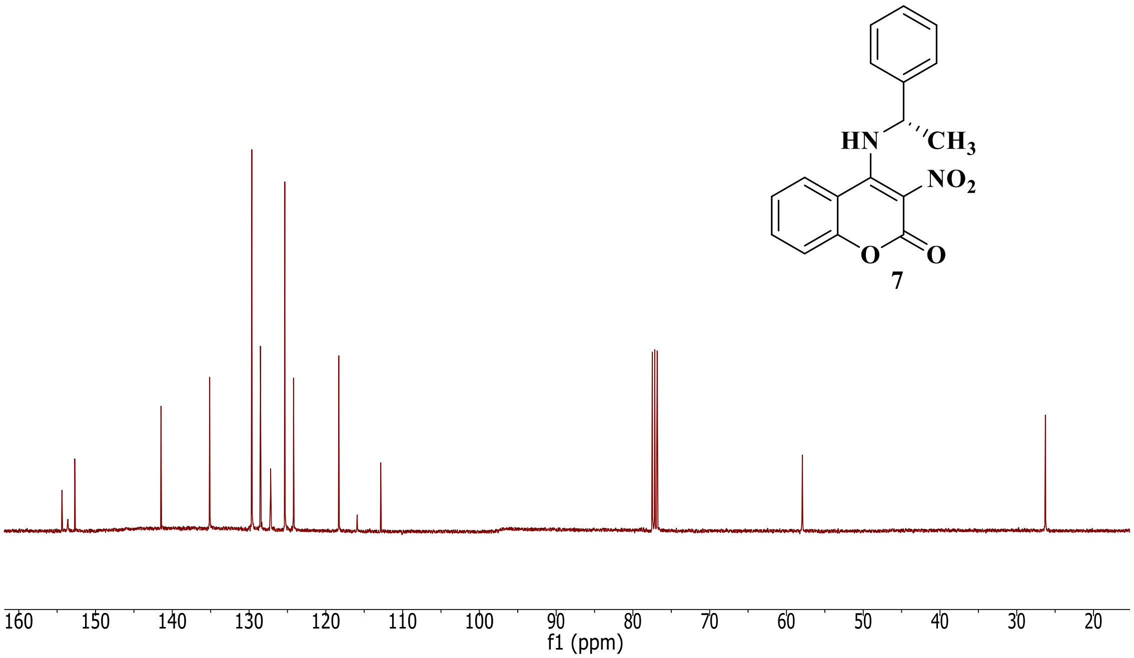

Supplementary Figure 6: ^{1}H NMR and ^{13}C NMR spectra of 4-(((1*S*,2*R*)-2-hydroxy-2,3-dihydro-1H-inden-1-yl)amino)-3-nitrocoumarin in $(CD_3)_2SO$

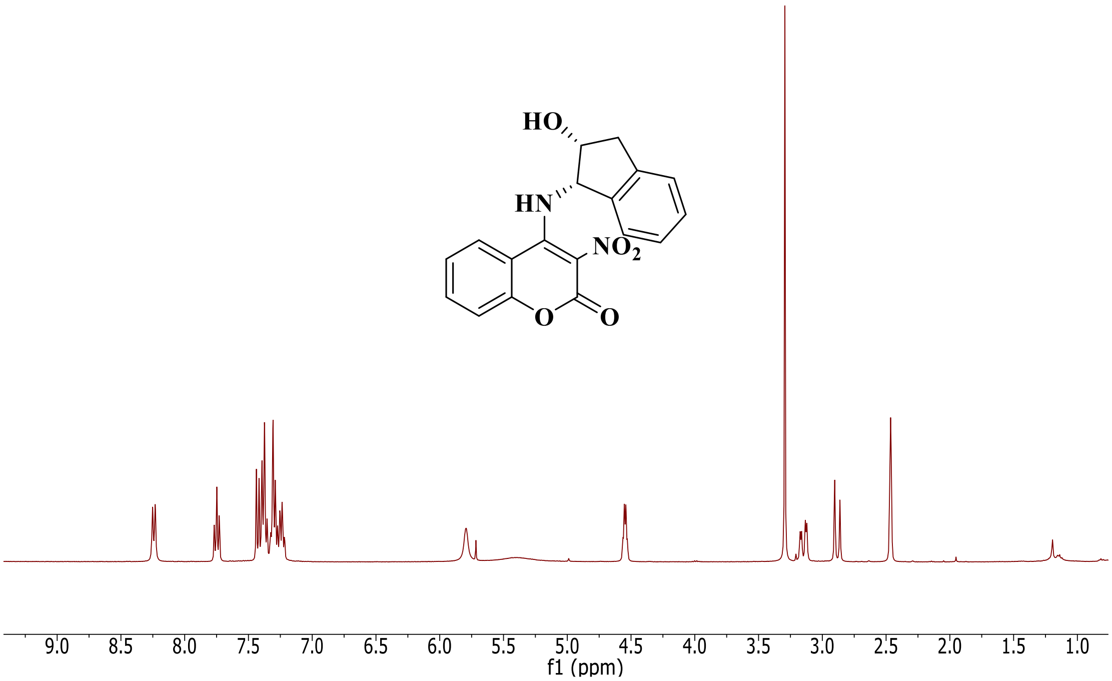

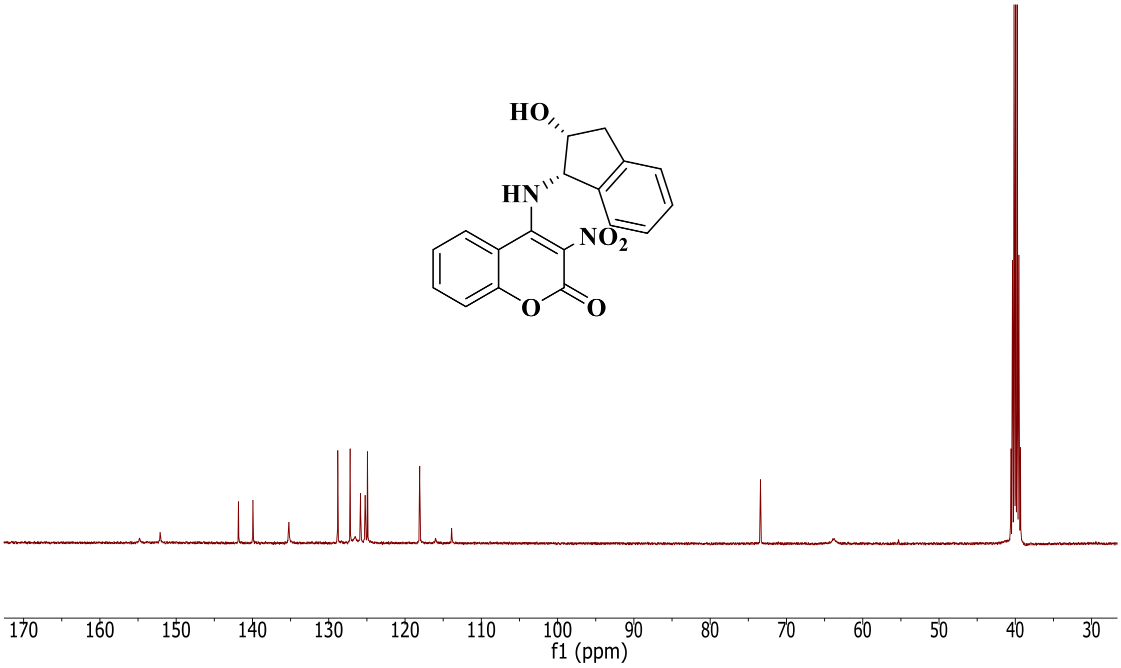

Supplementary Figure 7: ^{1}H NMR and ^{13}C NMR spectra of (*R*)-3-nitro-4-(*N*,α-dimethylbenzyl)amino)coumarin in $CDCl_3$

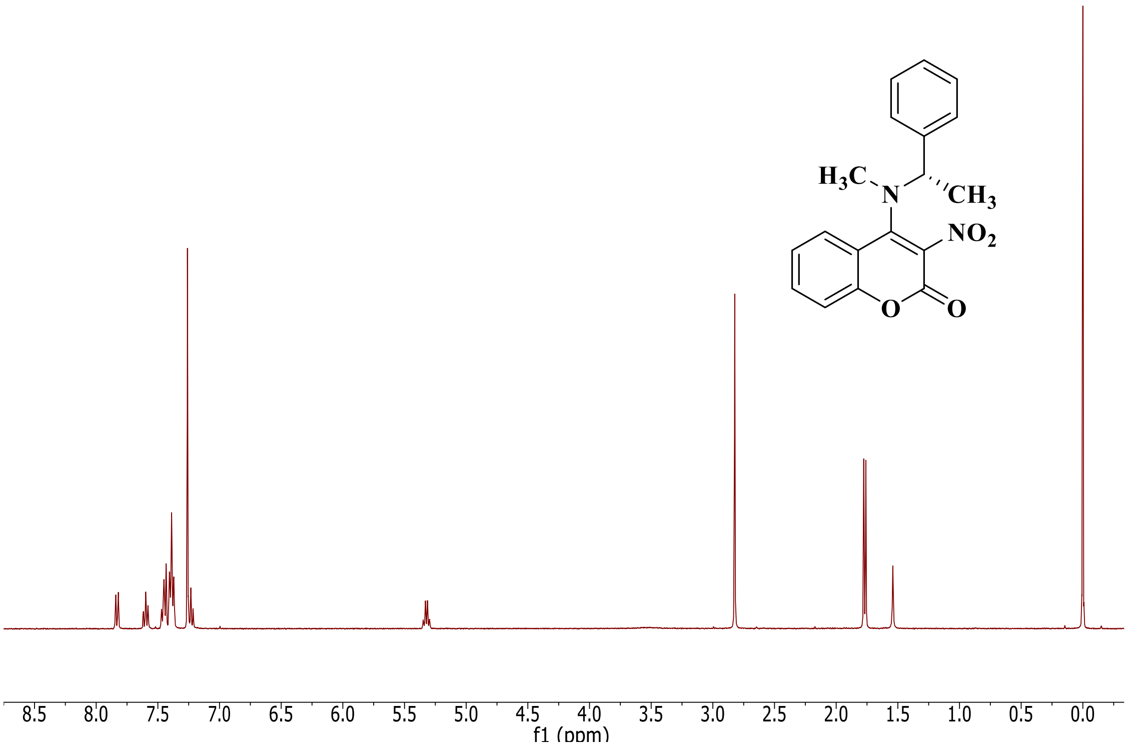

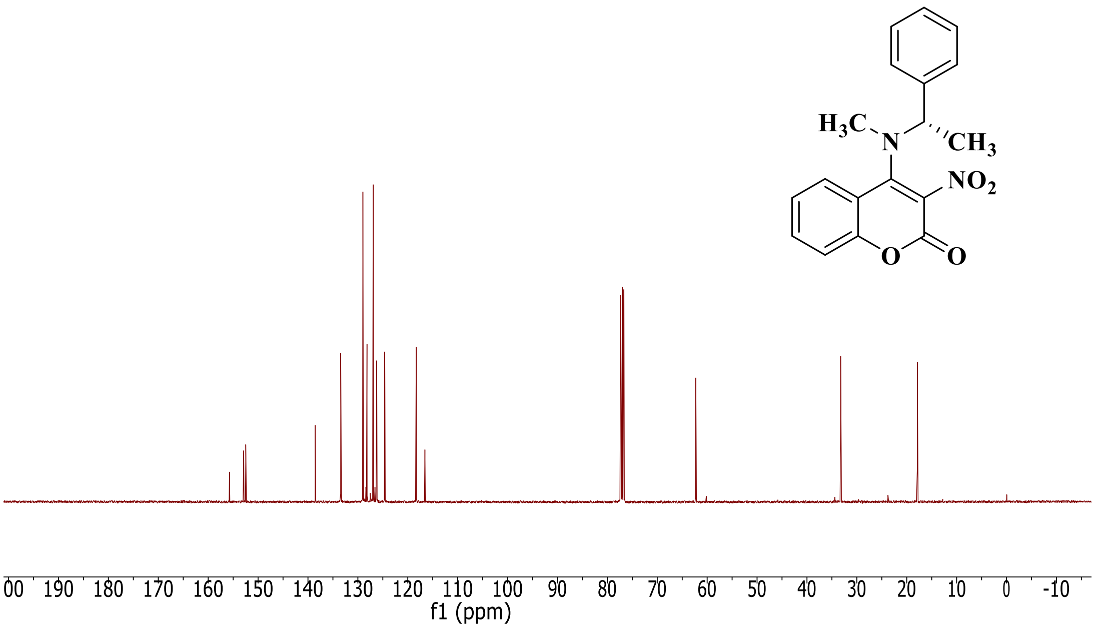

Supplementary Figure 8: CD spectra obtained using 4-chloro-3-nitrocoumarin (**3**, red), 4-chlorocoumarin (**1**, blue) and 4-bromocoumarin (**2**, yellow) with (*S*)-1-phenylethylamine (**8**) at room temperature

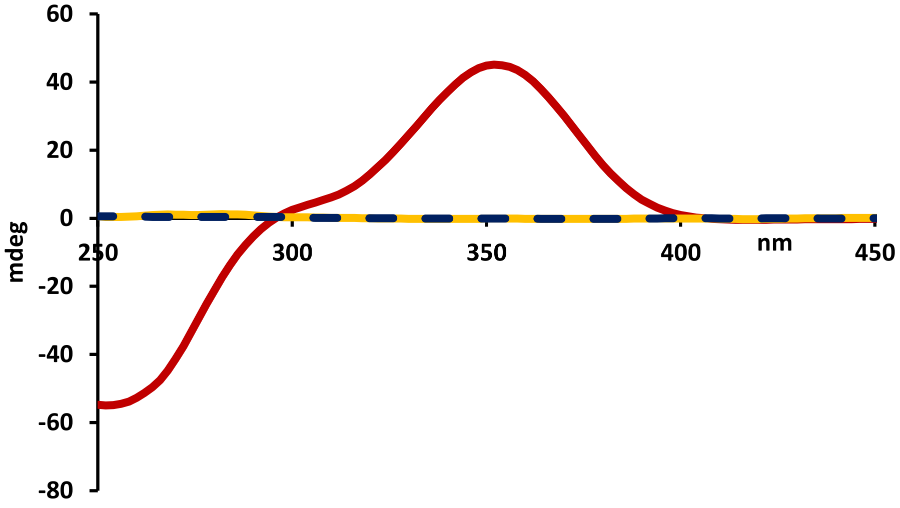

Supplementary Figure 9: CD of (*S*)-4-((1-phenylethyl)amino)coumarin (**6**) in chloroform taken at 0.24 mM

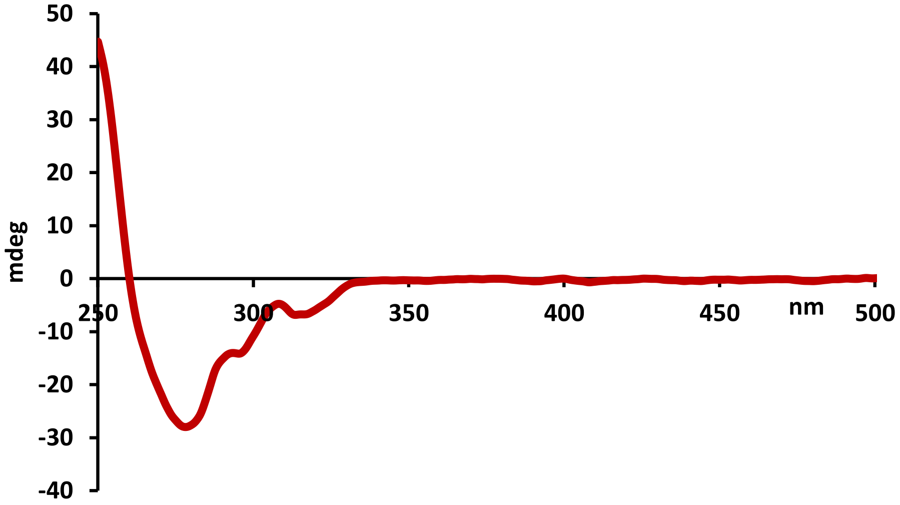

Supplementary Figure 10: CD spectra obtained using 4-chloro-3-nitrocoumarin (**3**) with (*S*)-1-phenylethylamine (**8**) (red) and (*R*)-1-phenylethylamine (**8**) (blue)

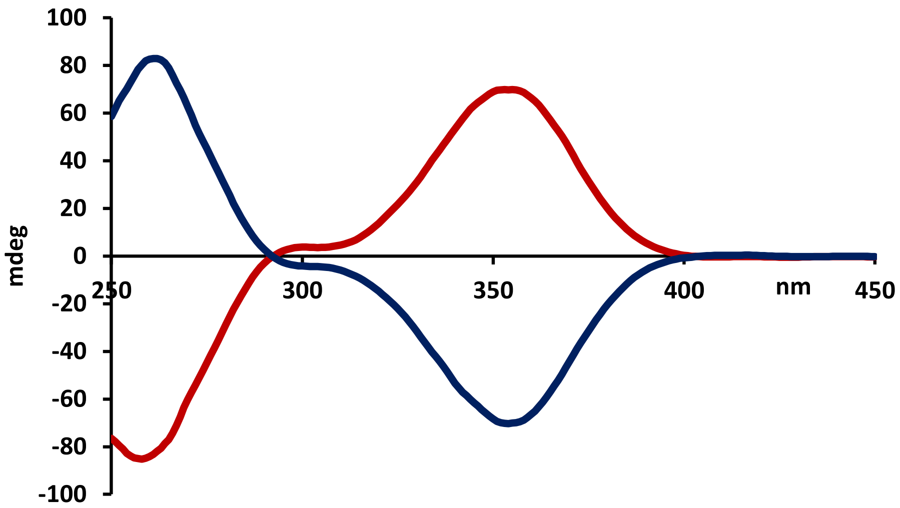

Supplementary Figure 11: CD spectra obtained using 4-bromo-3-nitrocoumarin (**4**) with (*S*)-1-phenylethylamine (**8**) (red) and (*R*)-1-phenylethylamine (**8**) (blue)

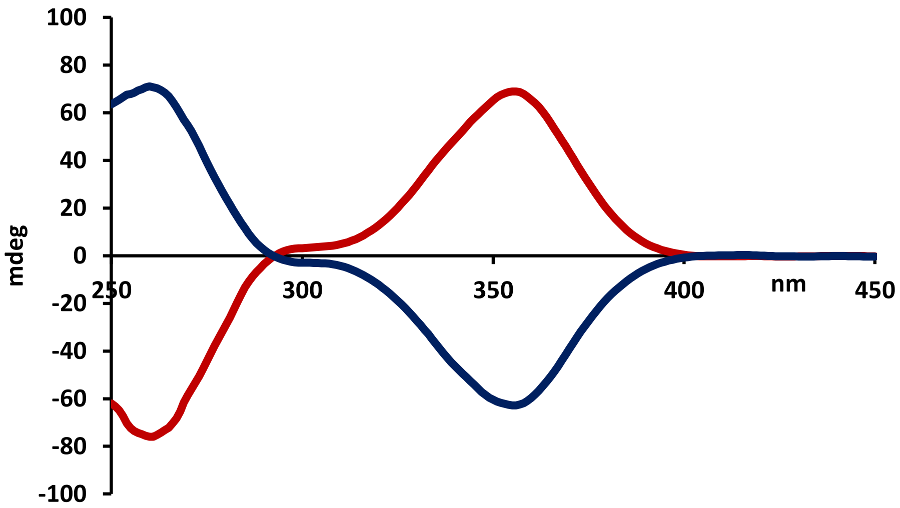

Supplementary Figure 12: CD spectra obtained using 4-iodo-3-nitrocoumarin (**5**) with (*S*)-1-phenylethylamine (**8**) (red) and (*R*)-1-phenylethylamine (**8**) (blue)

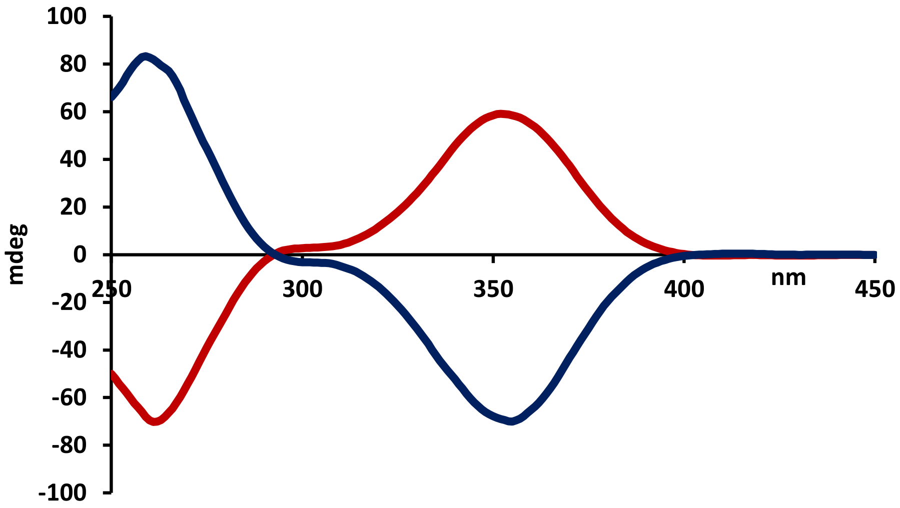

Supplementary Figure 13: Comparison of the CD spectra obtained with (*S*)-1-phenylethylamine (**8**) and probe **3** (red), **4** (blue) and **5** (yellow).

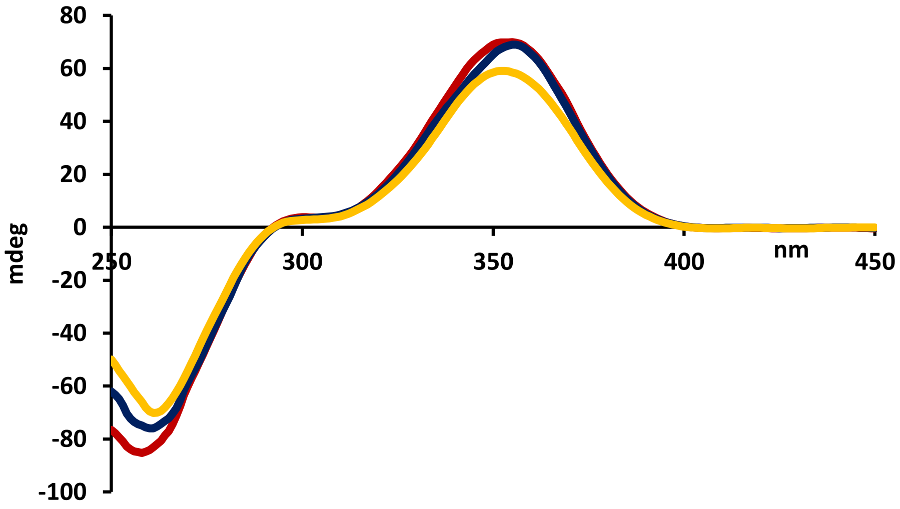

Supplementary Figure 14: CD comparison of the sensing of (*S*)-phenylethylamine (**8**) with probe **3** in different solvents with Et_3N

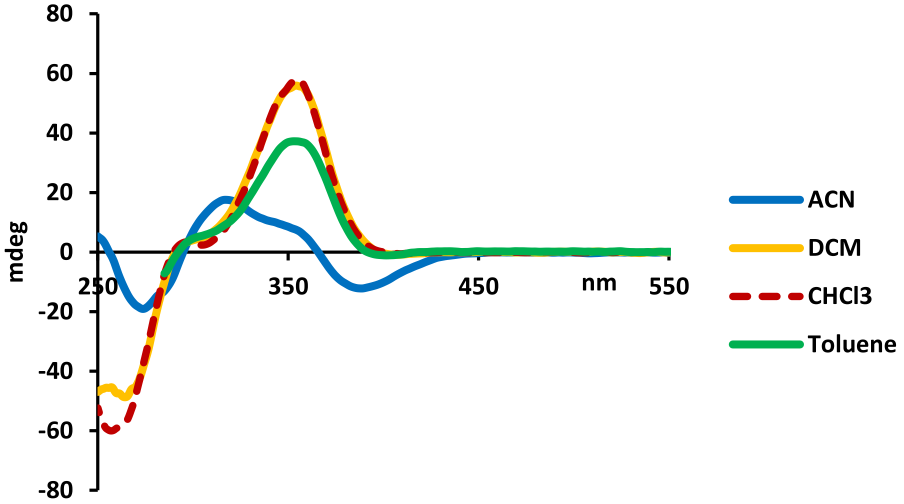

Supplementary Figure 15: CD comparison of the sensing of (*S*)-phenylethylamine (**8**) with probe **3** in different solvents with TBAOH

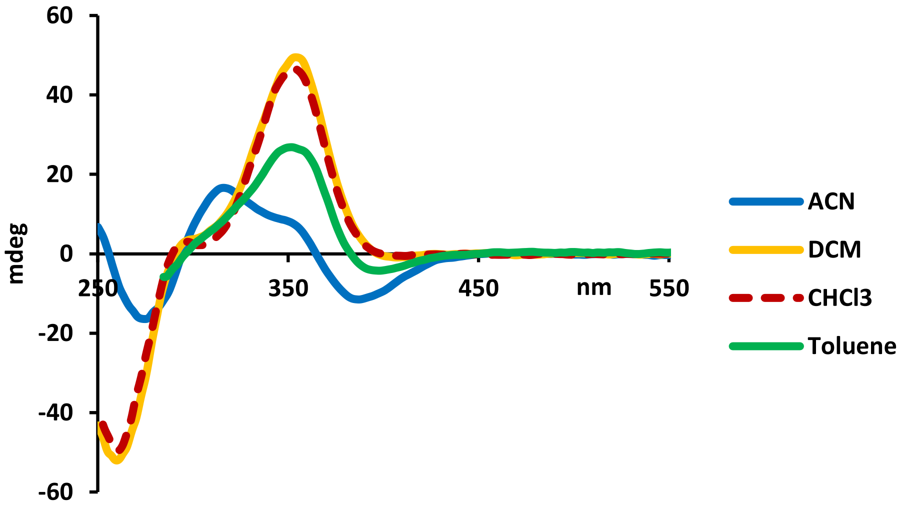

Supplementary Figure 16: CD comparison of the sensing of (*S*)-phenylethylamine (**8**) with probe **3** in different solvents in the absence of base

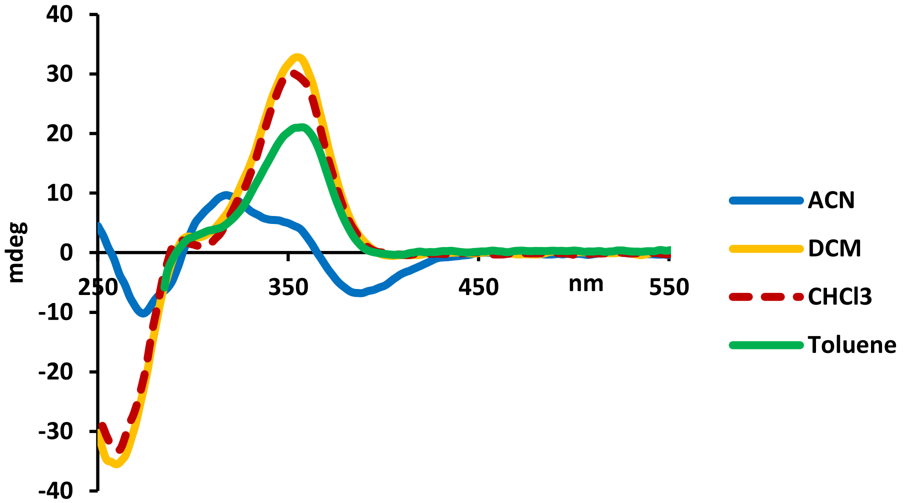

Supplementary Figure 17: Comparison of the CD spectra of the isolated product (**7**) (red) with the reaction mixture (blue)

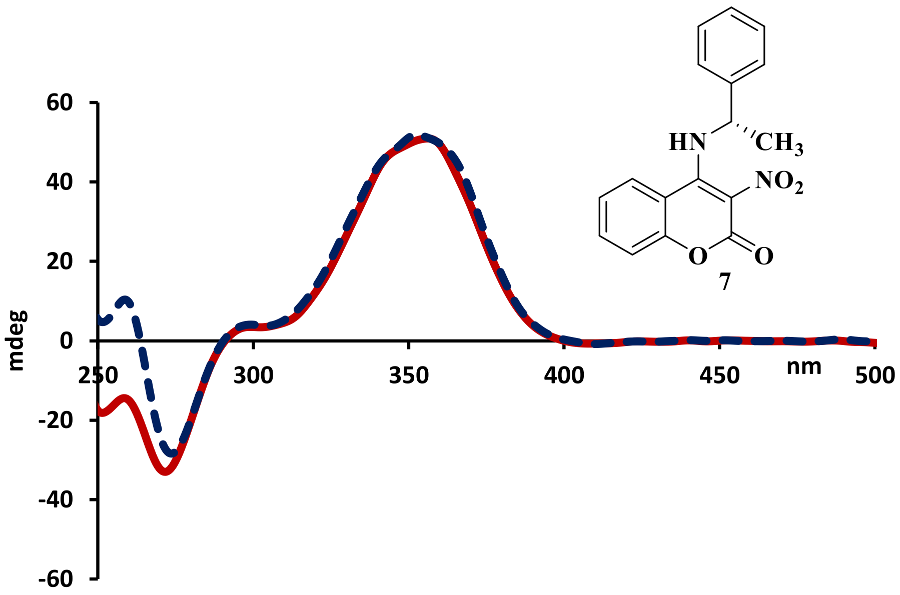

Supplementary Figure 18: Comparison of the isolated product (red) with the reaction mixture (blue)

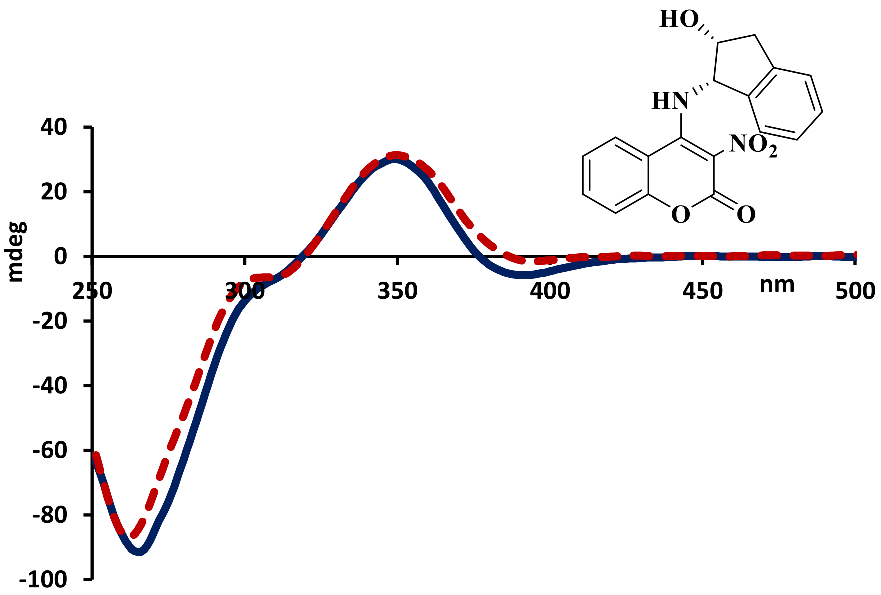

Supplementary Figure 19: Comparison of the isolated product (red) with the reaction mixture (blue)

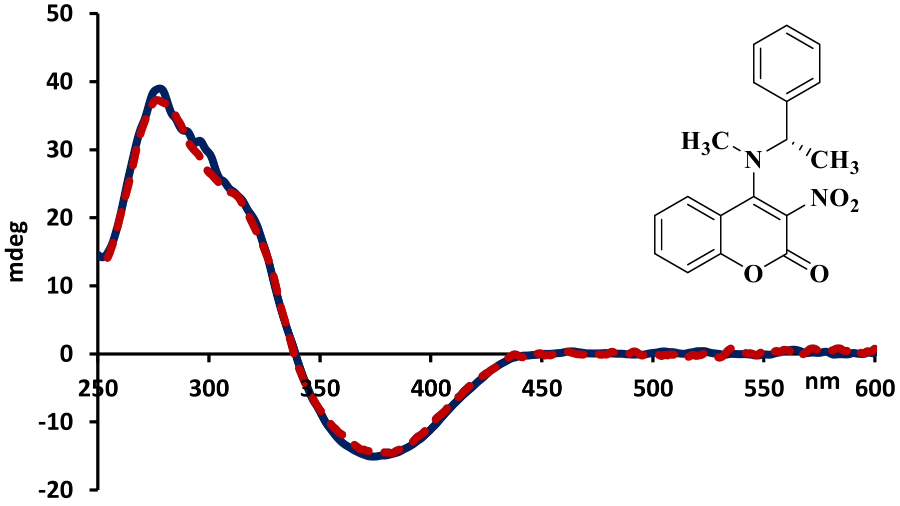

Supplementary Figure 20: ^{1}H NMR spectra of the reaction between probe **3** and (*S*)-1-phenylethylamine (**8**)

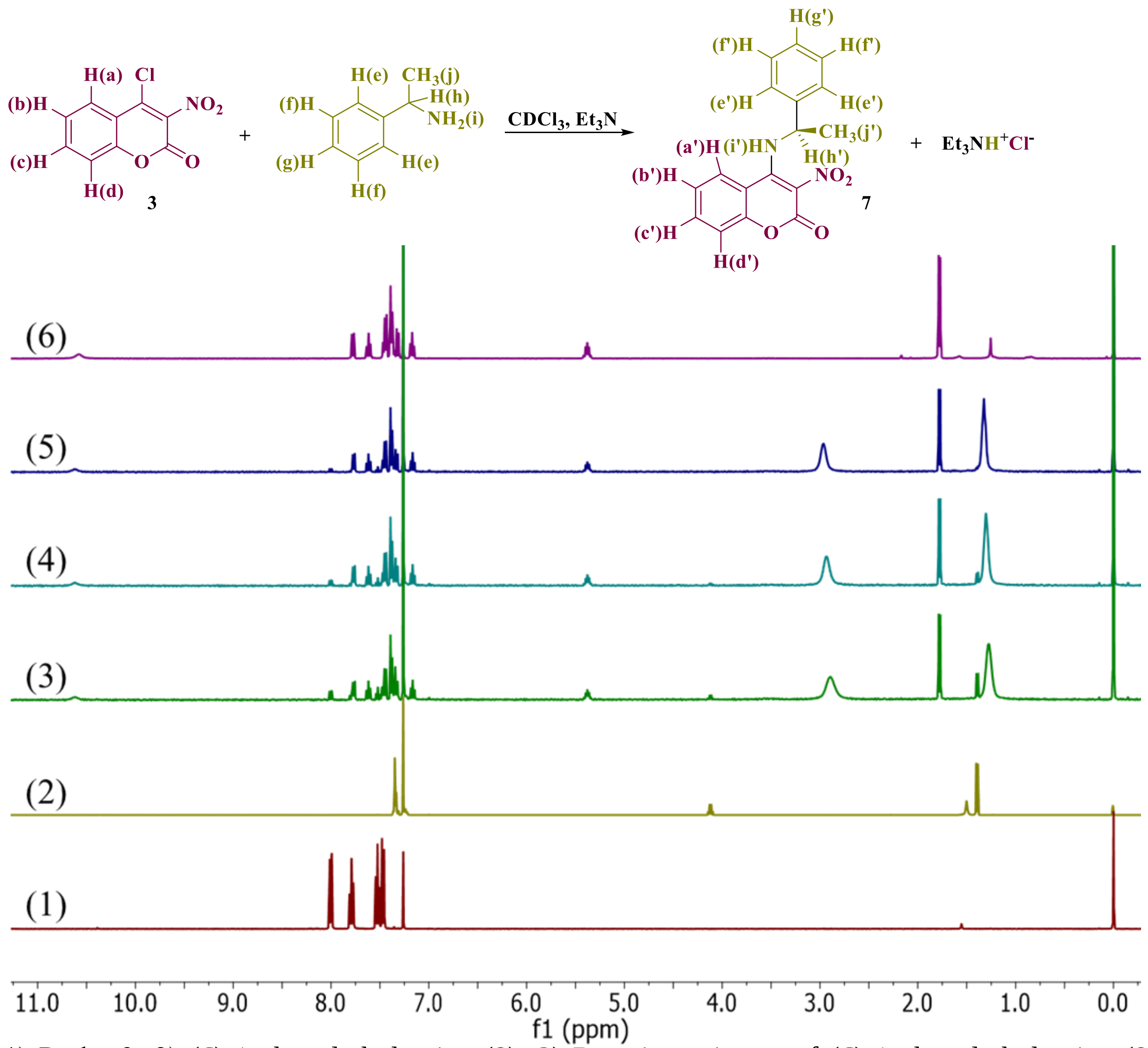

1) Probe **3**; 2) (*S*)-1-phenylethylamine (**8**); 3) Reaction mixture of (*S*)-1-phenylethylamine (**8**), Et_3N and probe **3** after 5 minutes; 4) Reaction mixture of (*S*)-1-phenylethylamine (**8**), Et_3N and probe **3** after 10 minutes; 5) Reaction mixture of (*S*)-1-phenylethylamine, Et_3N and probe **3** after 15 minutes; 6) 3-nitro-4-((1-phenylethyl)amino)coumarin (**7**); isolated product of the reaction between (*S*)-1-phenylethylamine, Et_3N and probe **3**.

Supplementary Figure 21: ^{1}H NMR excerpts of the spectra shown above

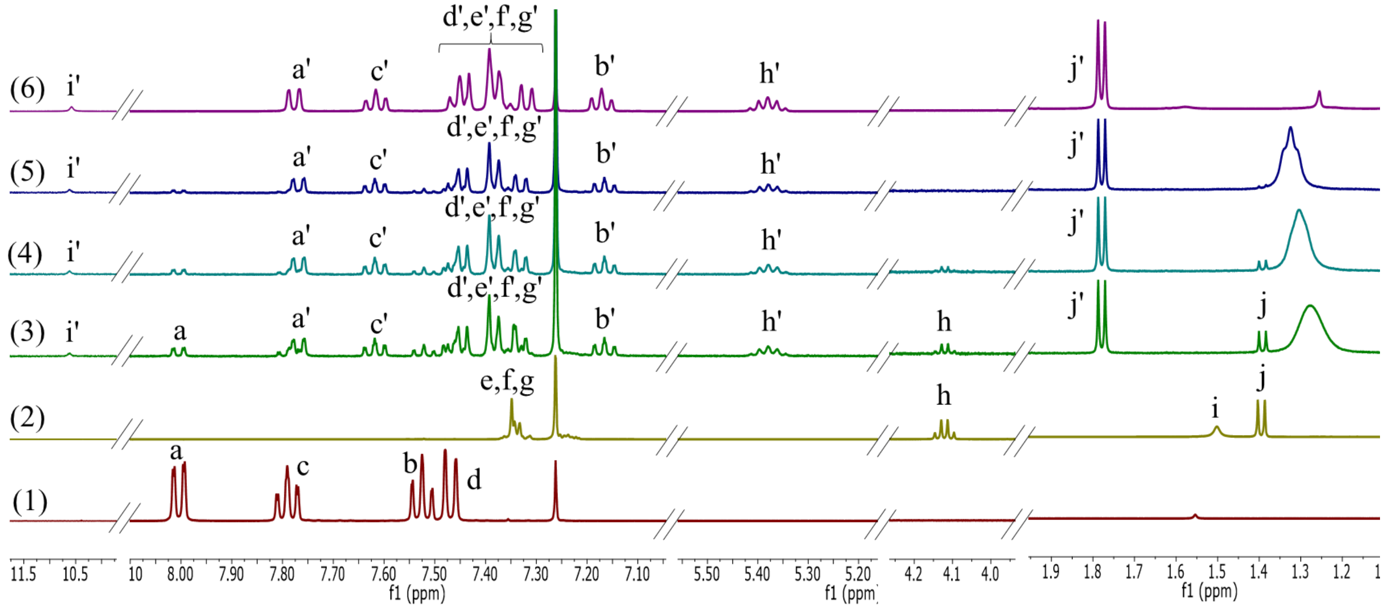

The signal at 1.39 ppm (H_j) of (*S*)-1-phenylethylamine (**8**) (spectrum 2) decreases in intensity with time and shows a downfield shift ($H_{j'}$, 1.78 ppm) in the reaction mixture (see spectra 3, 4, and 5).

The signal at 4.12 ppm (H_h) of (*S*)-1-phenylethylamine (**8**) (spectrum 2) decreases in intensity with time and shows a downfield shift ($H_{h'}$, 5.38 ppm) in the reaction mixture (see spectra 3, 4, and 5).

The signal at 8.00 ppm (H_a) of probe **3** (spectrum 1) decreases in intensity with time and shows an upfield shift ($H_{a'}$, 7.78 ppm) in the reaction mixture (see spectra 3, 4, and 5).

The assignment of $H_{i'}$ was based on the fact that it disappeared upon addition of CD_3OD.

Supplementary Figure 22: UV analysis of the reaction between (*S*)-1-phenylethylamine (**8**) and probe **3**

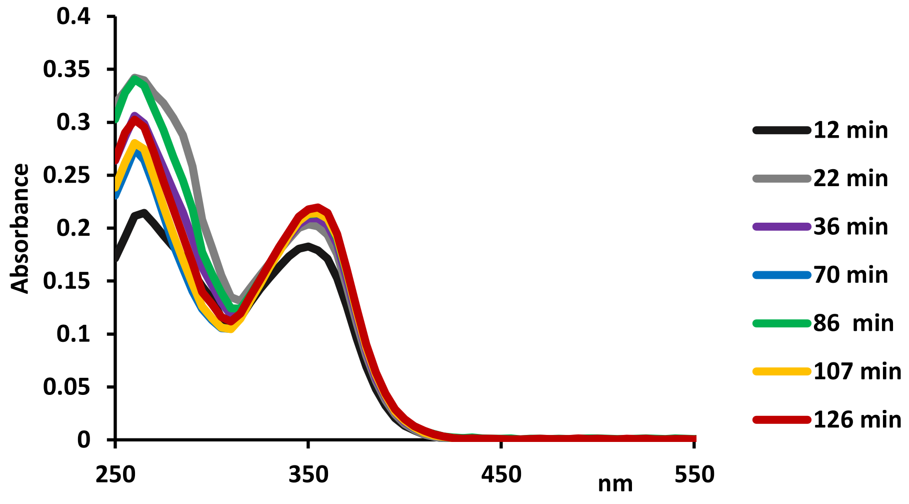

Supplementary Figure 23: Absorbance (355 nm) vs. time plot

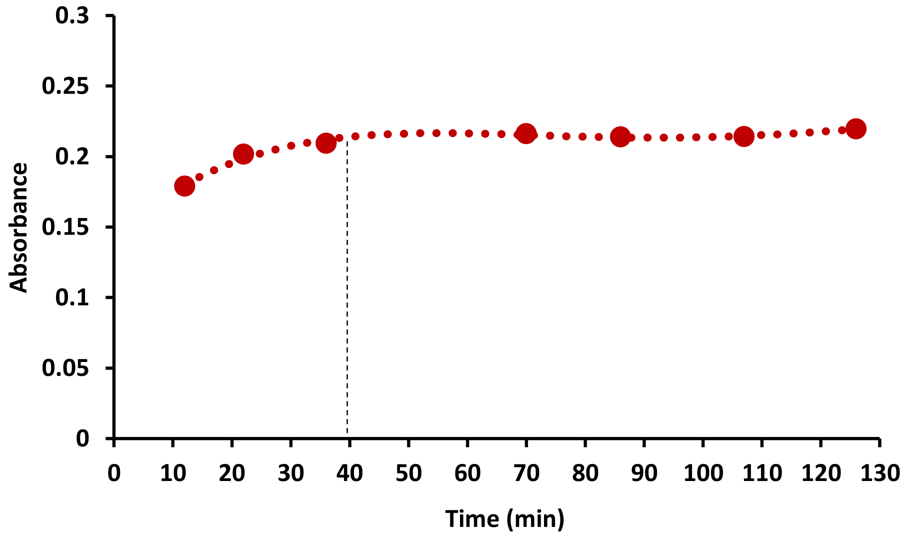

Supplementary Figure 24: UV analysis of the reaction between (*S*)-1-phenylethylamine (**8**) and probe **4**

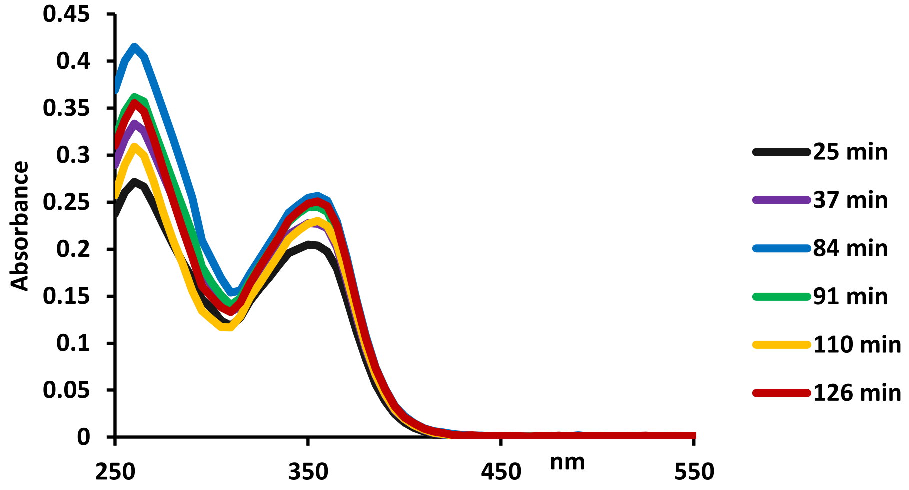

Supplementary Figure 25: Absorbance (355 nm) vs. time plot

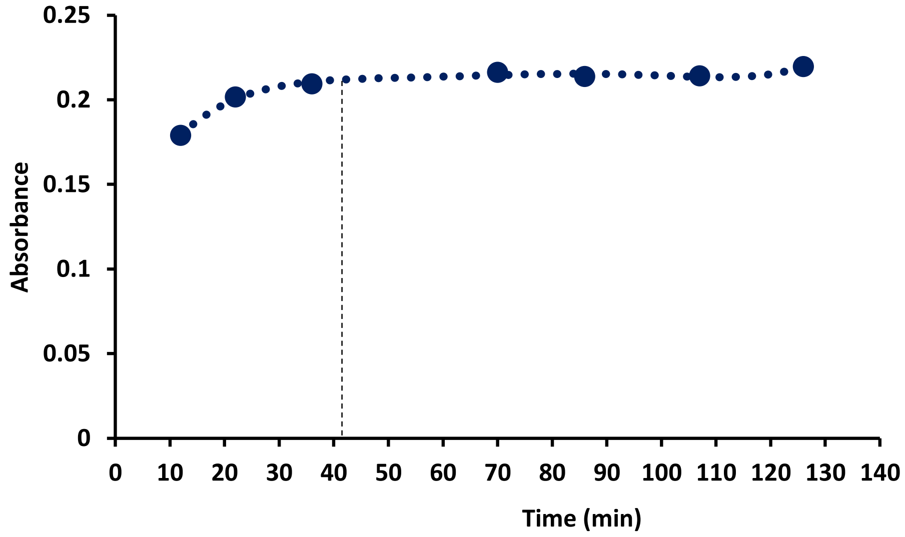

Supplementary Figure 26: UV analysis of the reaction between (*S*)-1-phenylethylamine (**8**) and probe **5**

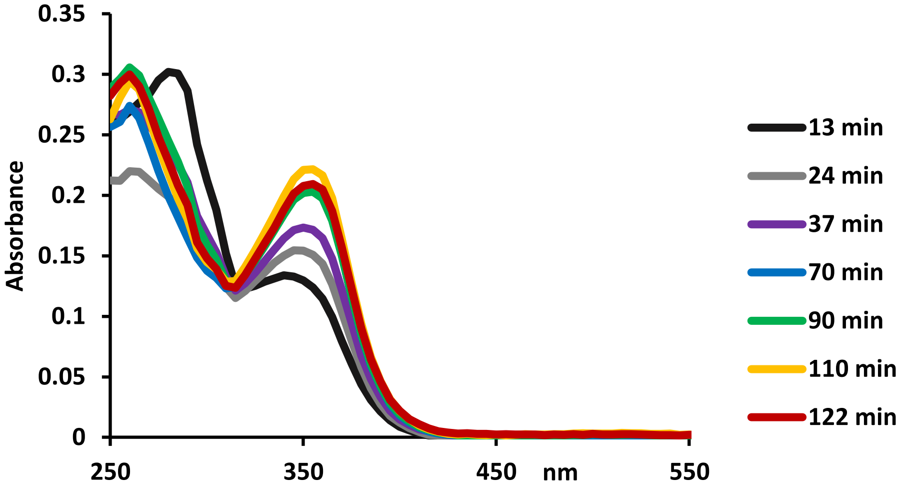

Supplementary Figure 27: Absorbance (355 nm) vs. time plot

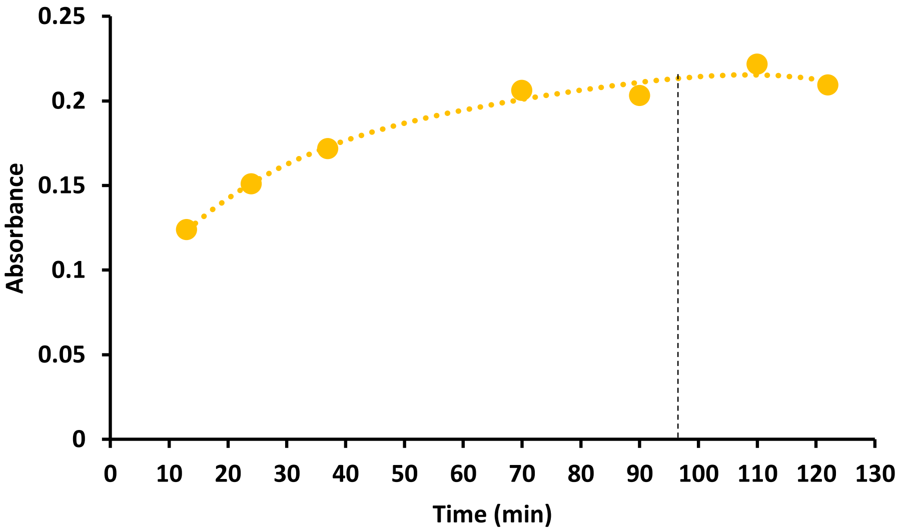

Supplementary Figure 28: CD sensing in protic solvents

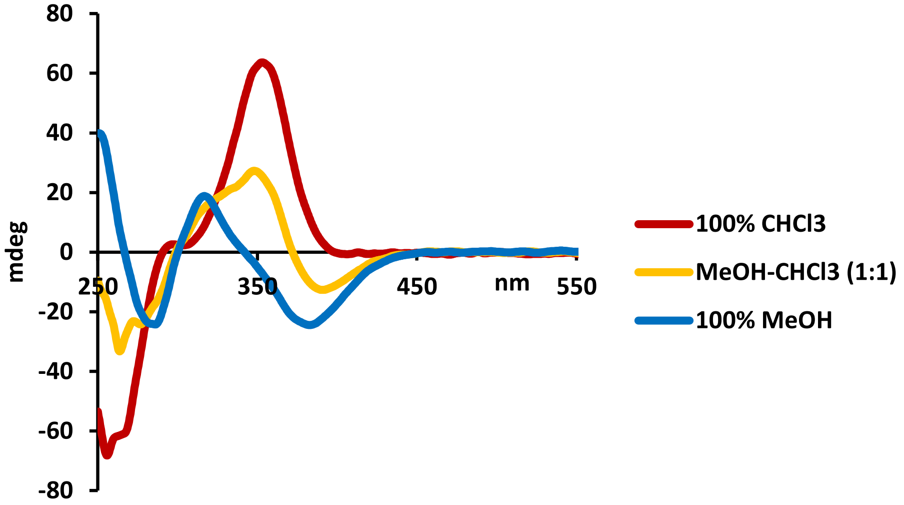

Supplementary Figure 29: CD spectra obtained from probe **3** with (*S*)-**8** (red) and (*R*)-**8** (blue)

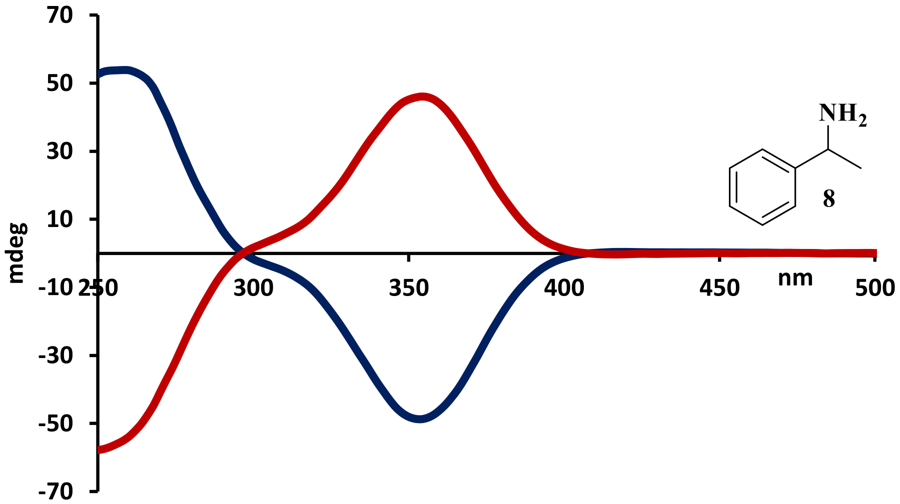

CD measurements were taken at 0.24 mM in chloroform.

Supplementary Figure 30: CD spectra obtained from probe **3** with (*S*)-**9** (red) and (*R*)-**9** (blue)

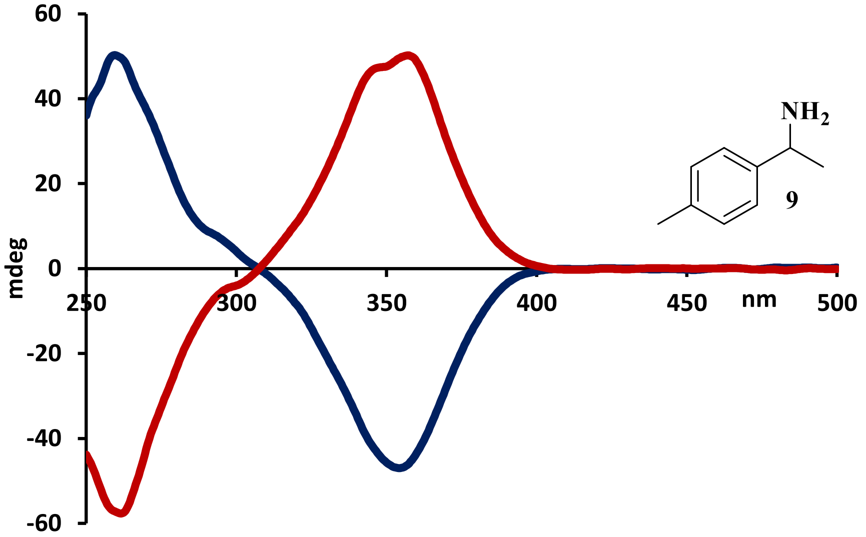

CD measurements were taken at 0.24 mM in chloroform.

Supplementary Figure 31: CD spectra obtained from probe **3** with (*S*)-**10** (red) and (*R*)-**10** (blue)

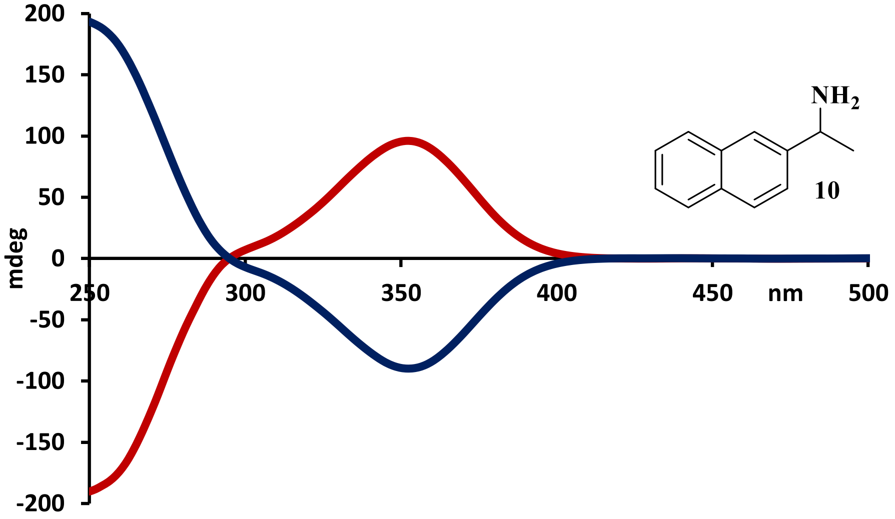

CD measurements were taken at 0.24 mM in chloroform.

Supplementary Figure 32: CD spectra obtained from probe **3** with (*S*)-**11** (red) and (*R*)-**11** (blue)

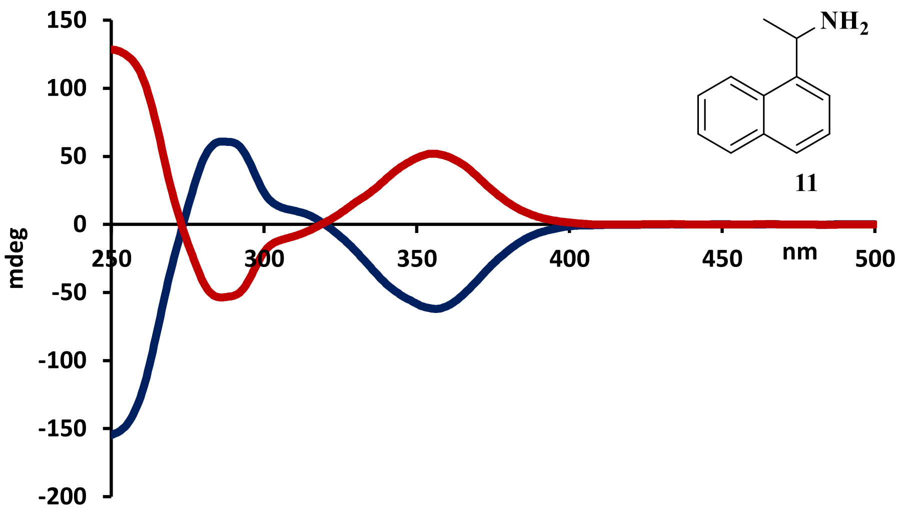

CD measurements were taken at 0.19 mM in chloroform.

Supplementary Figure 33: CD spectra obtained from probe **3** with (*S*)-**12** (red) and (*R*)-**12** (blue)

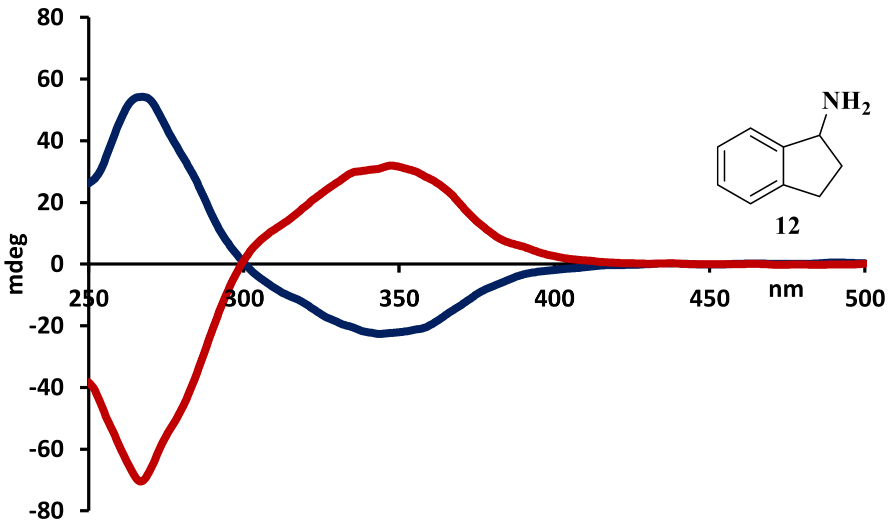

CD measurements were taken at 0.35 mM in chloroform.

Supplementary Figure 34: CD spectra obtained from probe **3** with (*S*)-**13** (red) and (*R*)-**13** (blue)

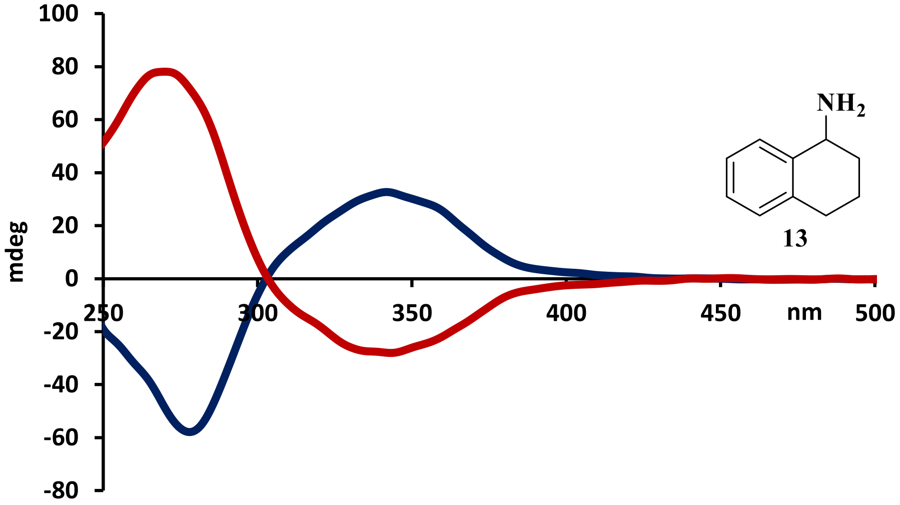

CD measurements were taken at 0.16 mM in chloroform.

Supplementary Figure 35: CD spectra obtained from probe **3** with (*S*)-**14** (red) and (*R*)-**14** (blue)

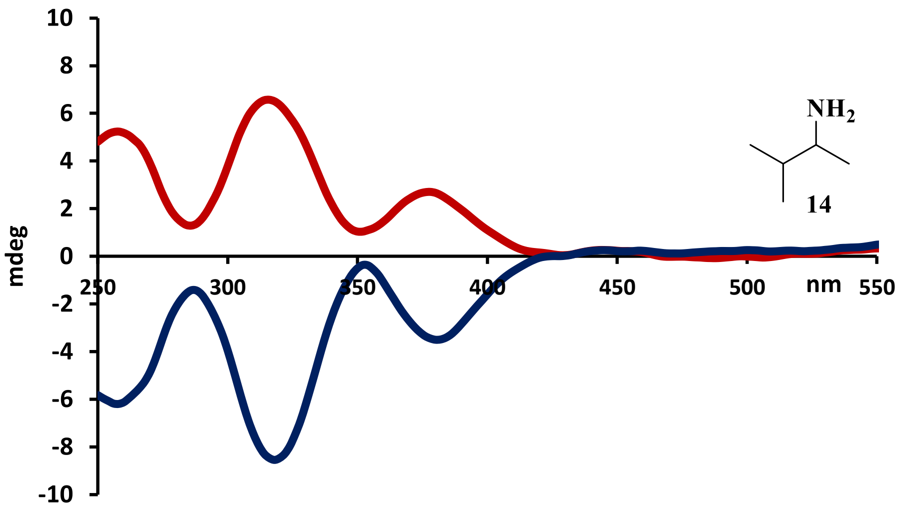

CD measurements were taken at 0.24 mM in chloroform.

Supplementary Figure 36: CD spectra obtained from probe **3** with (*S*)-**15** (red) and (*R*)-**15** (blue)

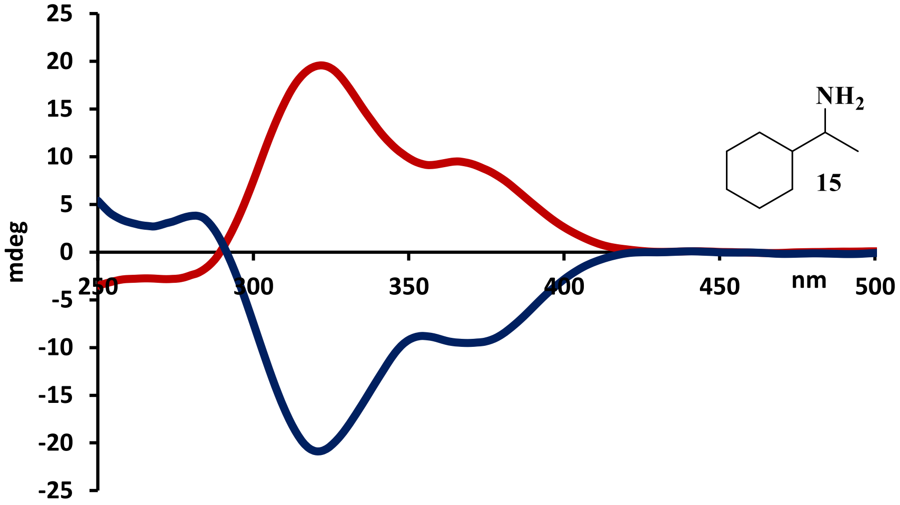

CD measurements were taken at 0.24 mM in chloroform.

Supplementary Figure 37: CD spectra obtained from probe **3** with (*S*)-**16** (red) and (*R*)-**16** (blue)

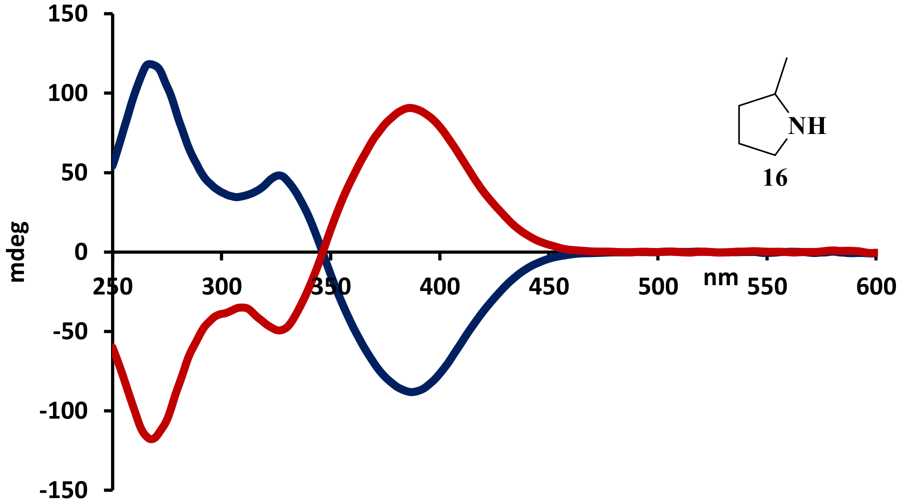

CD measurements were taken at 0.24 mM in chloroform.

Supplementary Figure 38: CD spectra obtained from probe **3** with (*S*)-**17** (red) and (*R*)-**17** (blue)

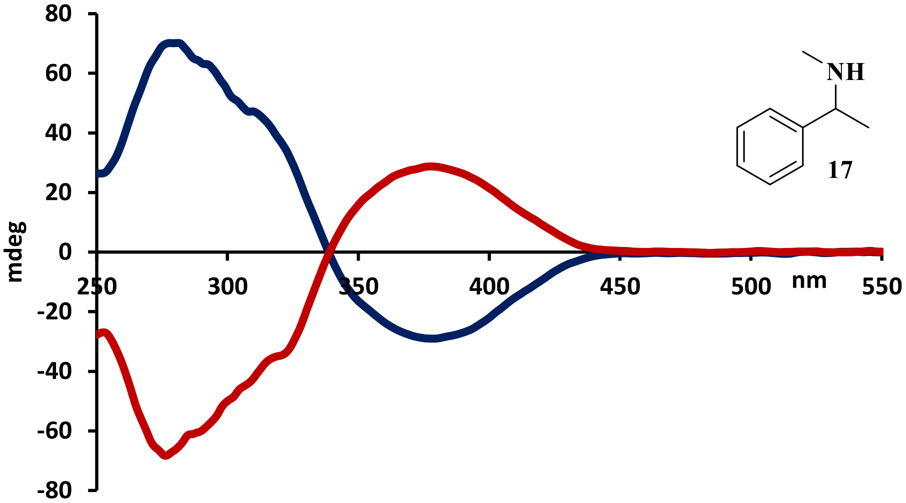

CD measurements were taken at 0.24 mM in chloroform.

Supplementary Figure 39: CD spectra obtained from *1 equivalent* of probe **3** with (*S*)-*trans*-**18** (red) and (*R*)-*trans*-**18** (blue)

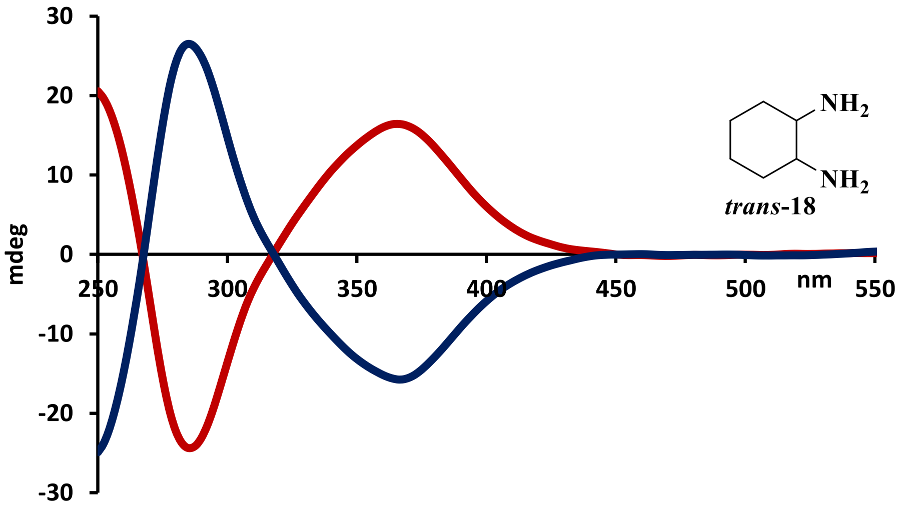

CD measurements were taken at 0.24 mM in chloroform.

Supplementary Figure 40: CD spectra obtained from with *2 equivalents* of probe **3** with (*S*)-*trans*-**18** (red) and (*R*)-*trans*-**18** (blue)

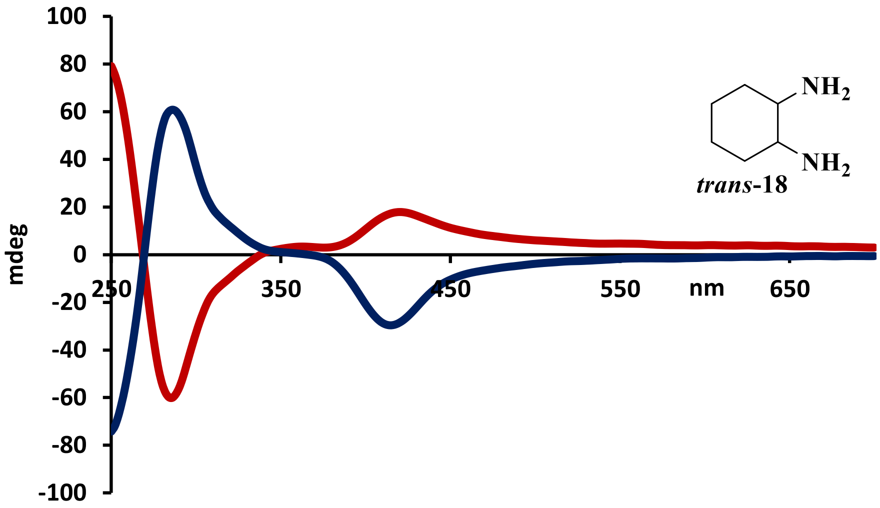

CD measurements were taken at 0.24 mM in chloroform.

Supplementary Figure 41: CD spectra obtained from probe **3** with (*S,S*)-*syn*-**19** (red) and (*R,R*)-*syn*-**19** (blue)

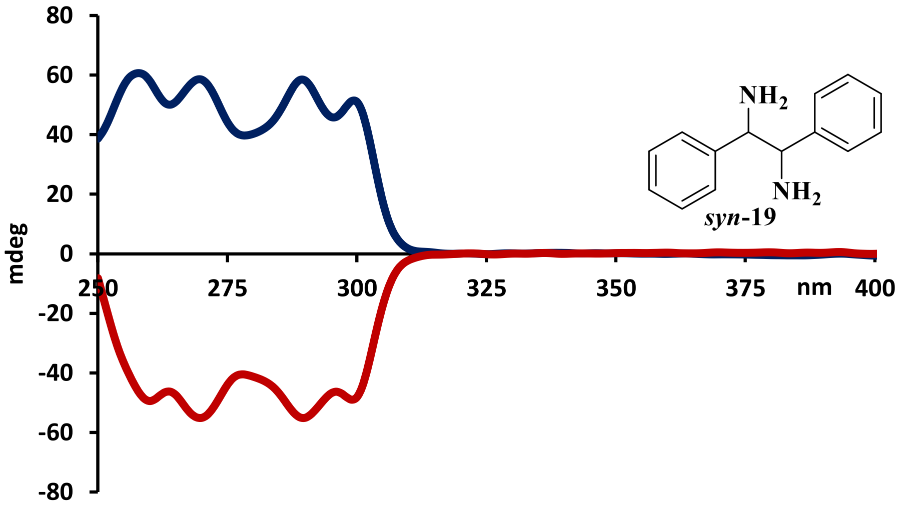

CD measurements were taken at 0.23 mM in chloroform.

Supplementary Figure 42: CD spectra obtained from probe **3** with (1*S*,2*S*)-*anti*-**20** (red) and (1*R*,2*R*)-*anti*-**20** (blue)

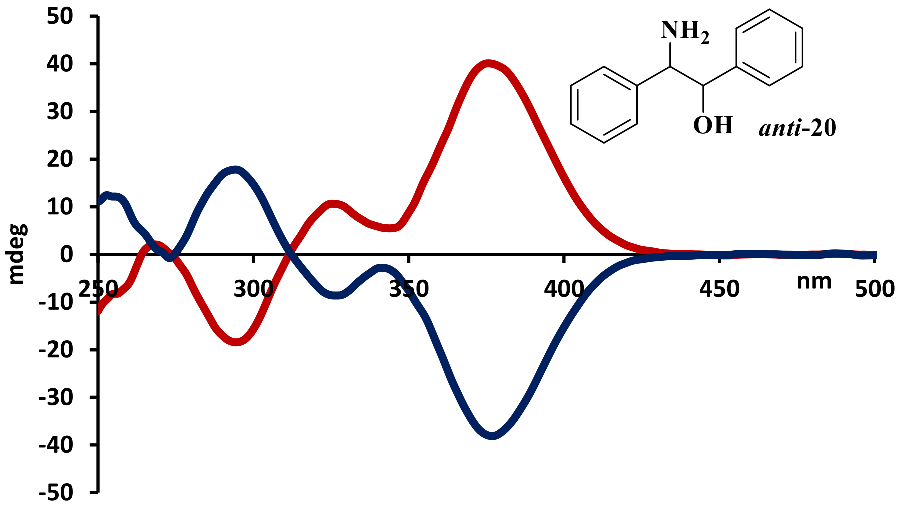

CD measurements were taken at 0.19 mM in chloroform.

Supplementary Figure 43: CD spectra obtained from probe **3** with (1*S*,2*R*)-*syn*-**21** (red) and (1*R*,2*S*)-*syn*-**21** (blue)

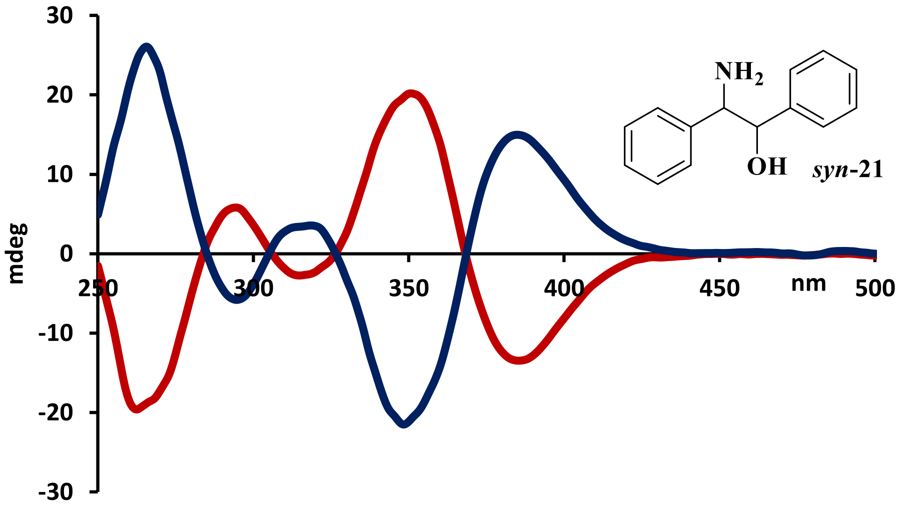

CD measurements were taken at 0.17 mM in chloroform.

Supplementary Figure 44: CD spectra obtained from probe **3** with (1*S*,2*R*)-*cis*-**22** (red) and (1*R*,2*S*)-*cis*-**22** (blue)

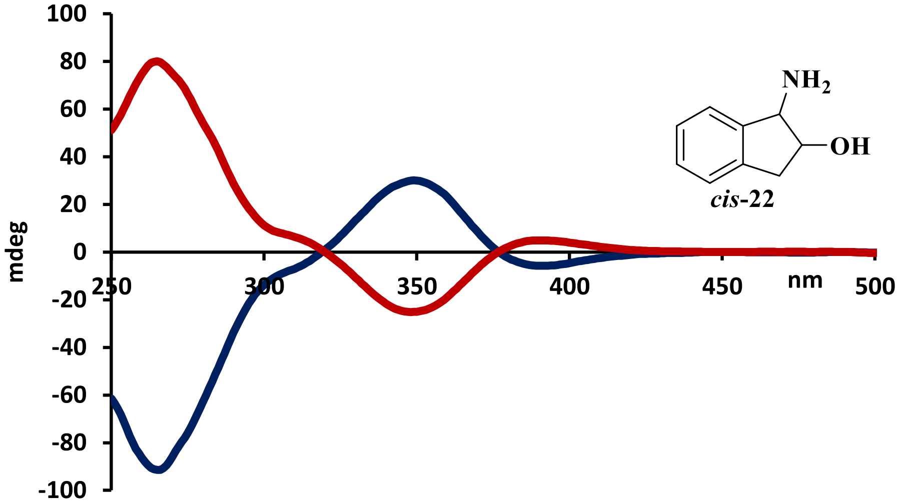

CD measurements were taken at 0.24 mM in chloroform.

Supplementary Figure 45: CD spectra obtained from probe **3** with (*S*)-**23** (red) and (*R*)-**23** **(**blue)

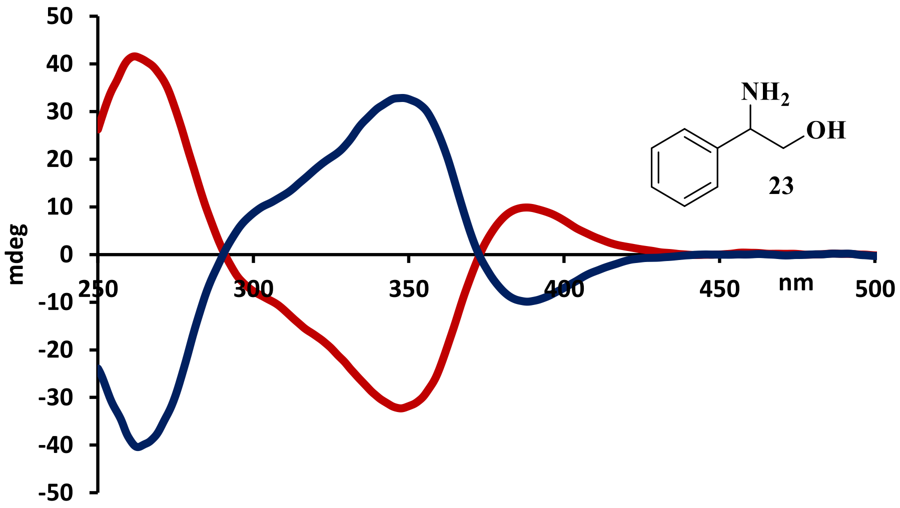

CD measurements were taken at 0.17 mM in chloroform

Supplementary Figure 46: CD spectra obtained from probe **3** with (*S*)-**24** (red) and (*R*)-**24** (blue)

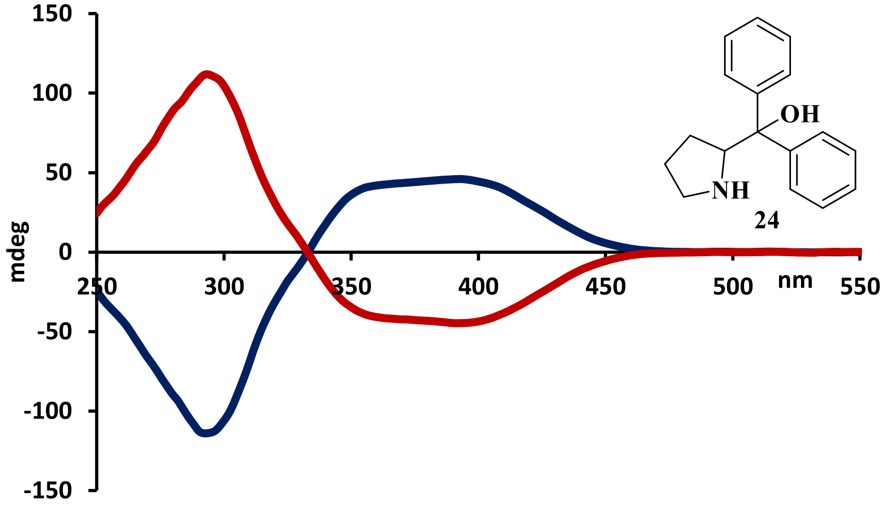

CD measurements were taken at 0.26 mM in chloroform.

Supplementary Figure 47: CD spectra obtained from probe **3** with (1*S*,2*R*)-*anti*-**25** (red) and (1*R*,2*S*)-*anti*-**25** **(**blue)

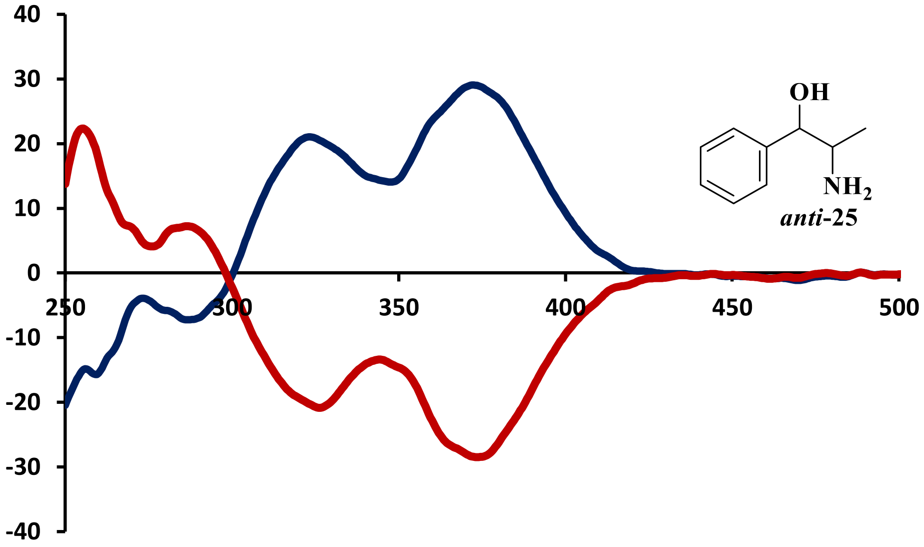

CD measurements were taken at 0.24 mM in chloroform.

Supplementary Figure 48: CD spectra obtained from probe **3** with (*S*)-**26** (red) and (*R*)-**26 (**blue)

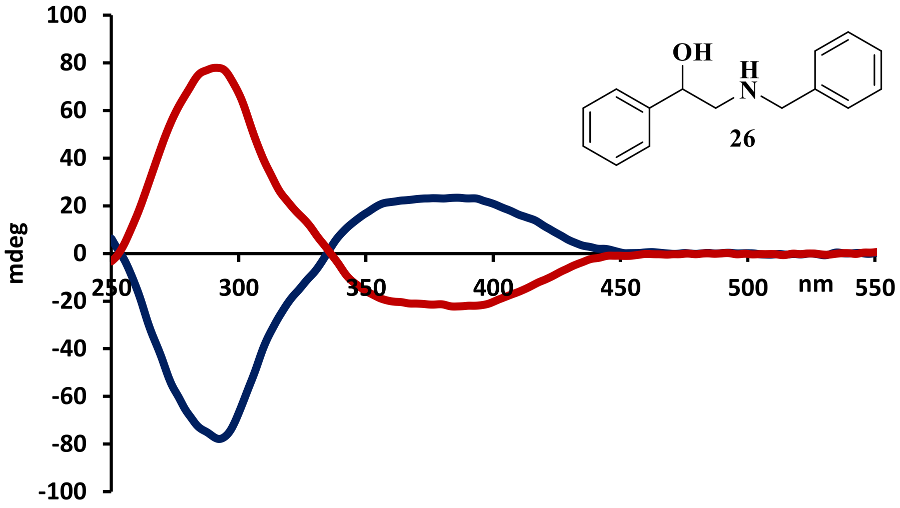

CD measurements were taken at 0.17 mM in chloroform.

Supplementary Figure 49: CD spectra obtained from probe **3** with (*S*)-**27** (red) and (*R*)-**27** (blue)

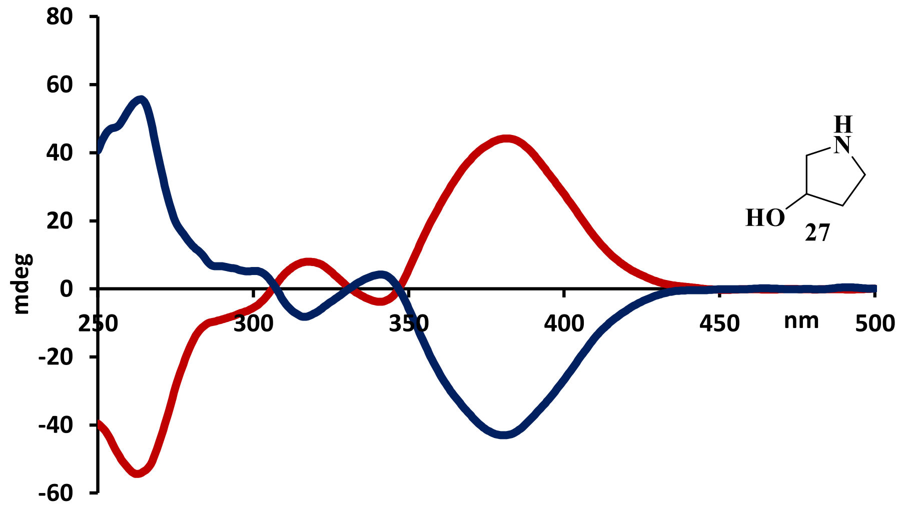

CD measurements were taken at 0.24 mM in chloroform.

Supplementary Figure 50: CD spectra obtained from probe **3** with (*S*)-**28** (red) and (*R*)-**28** (blue)

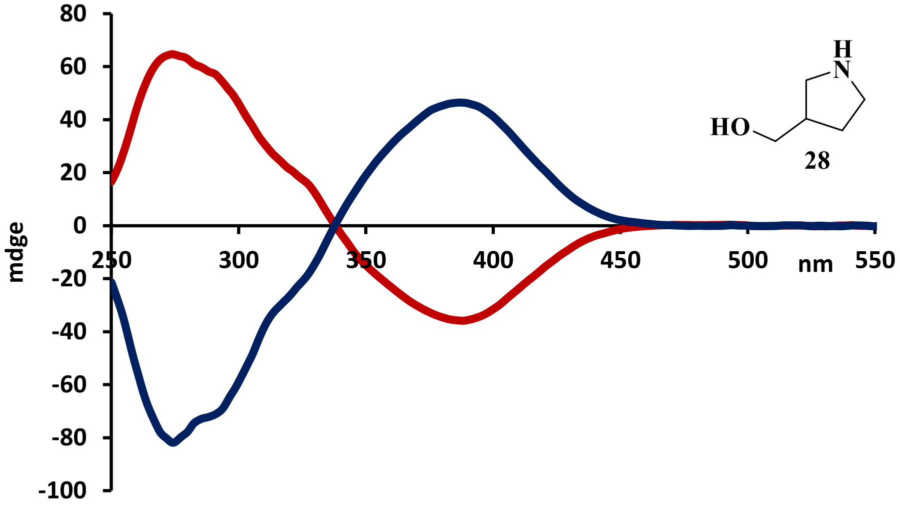

CD measurements were taken at 0.24 mM in chloroform

Supplementary Figure 51: CD spectra obtained from probe **3** with (1*S*,2*R*)-**29** (red) and (1*R*,2*S*)-**29 (**blue)

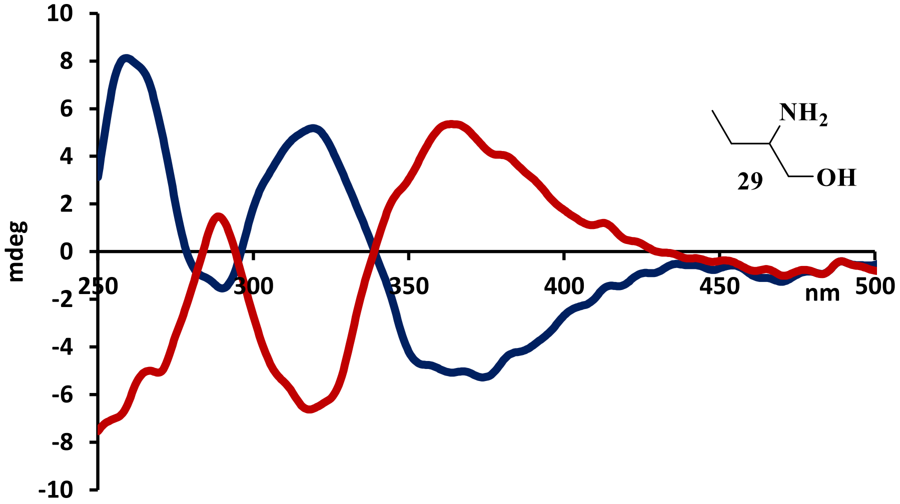

CD measurements were taken at 0.24 mM in chloroform.

Supplementary Figure 52: CD spectra obtained from probe **3** with (*S*)-**30** (red) and (*R*)-**30** **(**blue)

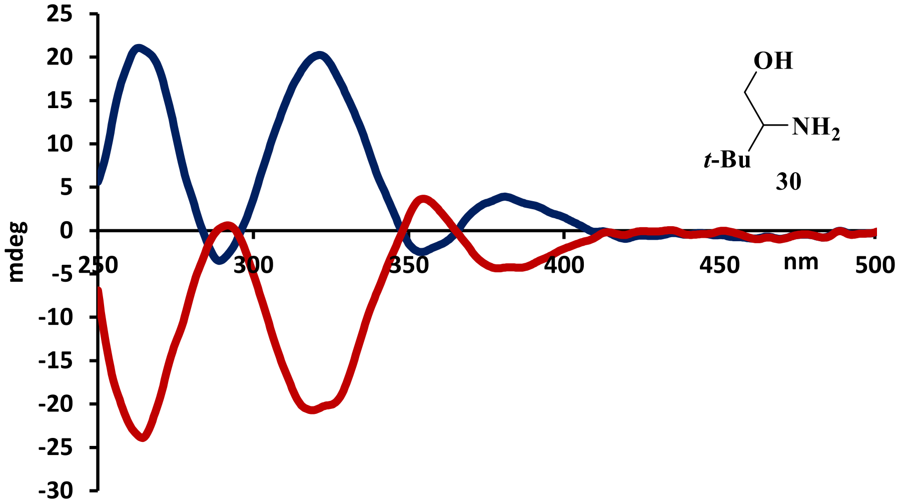

CD measurements were taken at 0.24 mM in chloroform.

Supplementary Figure 53: CD spectra obtained from probe **3** with (*S*)-**31** (red) and (*R*)-**31** **(**blue)

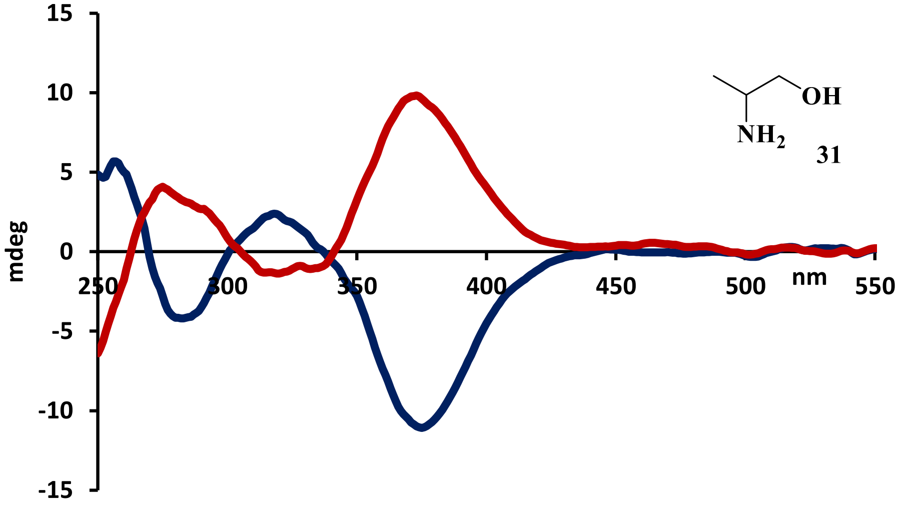

CD measurements were taken at 0.24 mM in chloroform.

Supplementary Figure 54: CD spectra obtained from probe **3** with (*S*)-**32** (red) and (*R*)-**32** **(**blue)

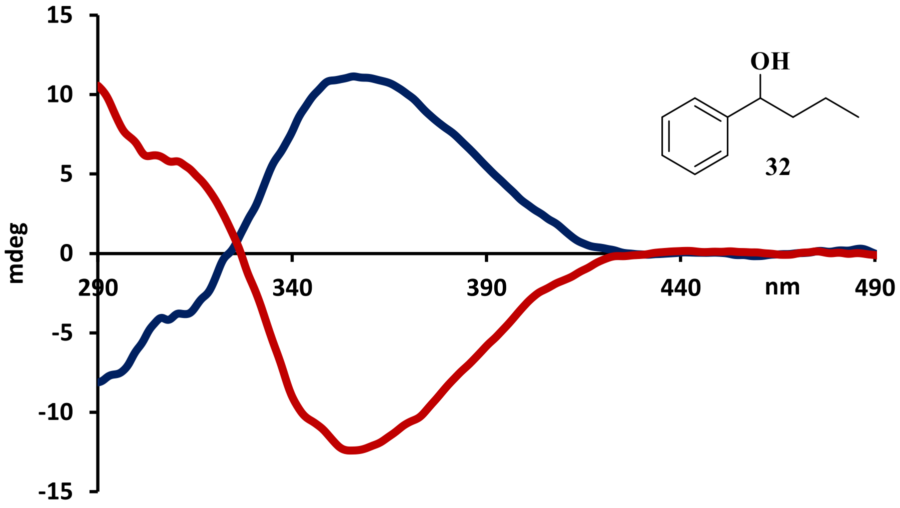

CD measurements were taken at 0.34 mM in THF.

Supplementary Figure 55: CD spectra obtained from probe **3** with (*S*)-**33** (red) and (*R*)-**33** **(**blue)

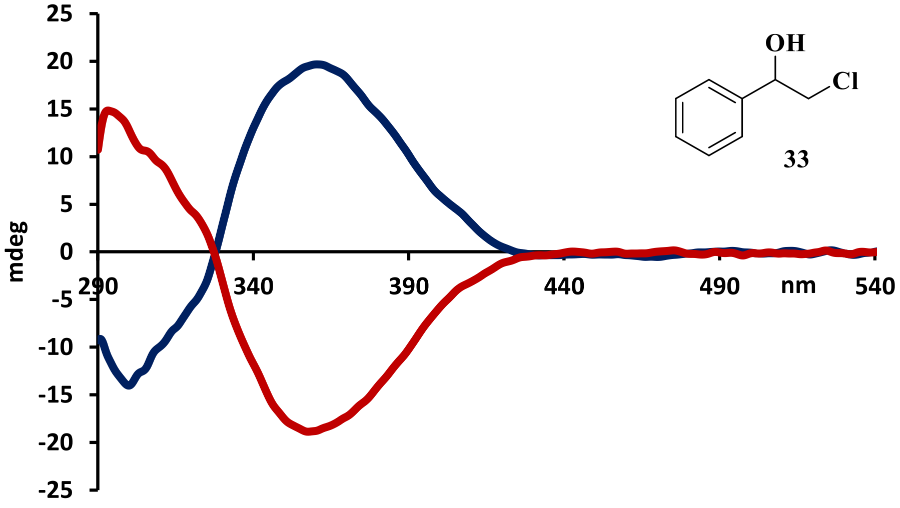

CD measurements were taken at 0.24 mM in THF.

Supplementary Figure 56: CD spectra obtained from probe **3** with (*S*)-**34** (red) and (*R*)-**34** **(**blue)

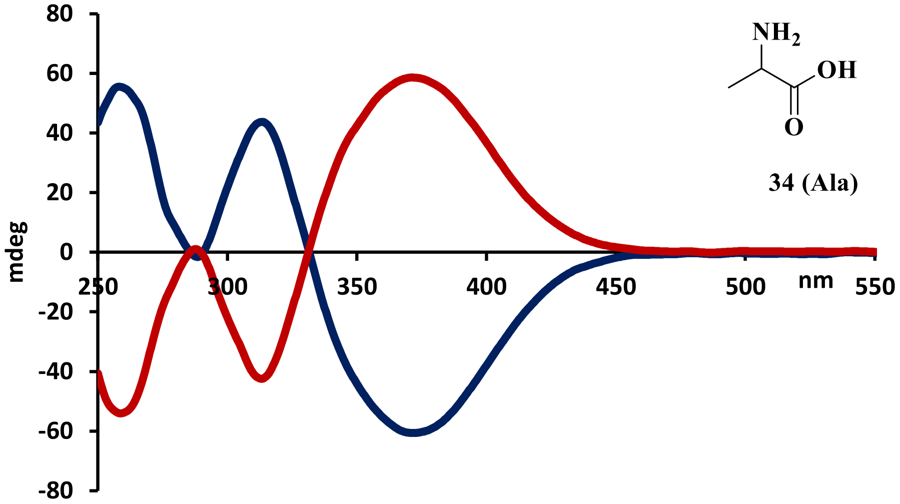

CD measurements were taken at 0.28 mM in acetonitrile.

Supplementary Figure 57: CD spectra obtained from probe **3** with (*S*)-**35** (red) and (*R*)-**35** **(**blue)

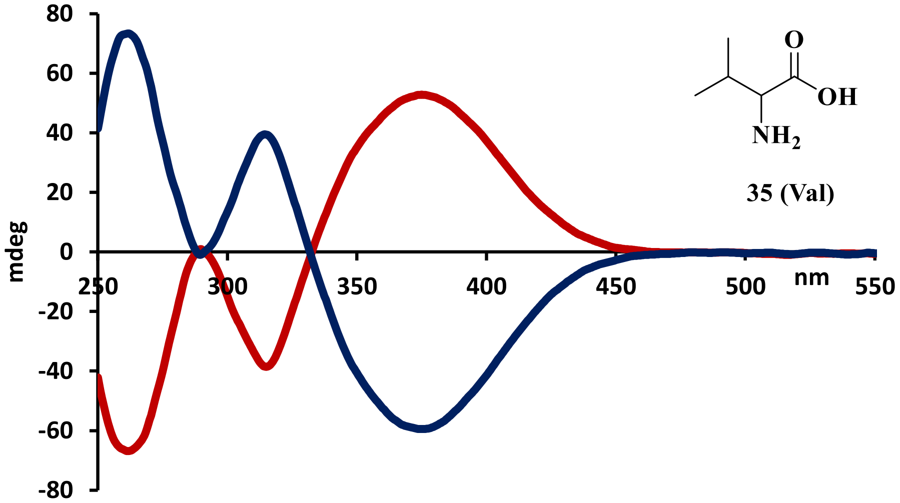

CD measurements were taken at 0.24 mM in acetonitrile.

Supplementary Figure 58: CD spectra obtained from probe **3** with (*S*)-**36** (red) and (*R*)-**36** **(**blue)

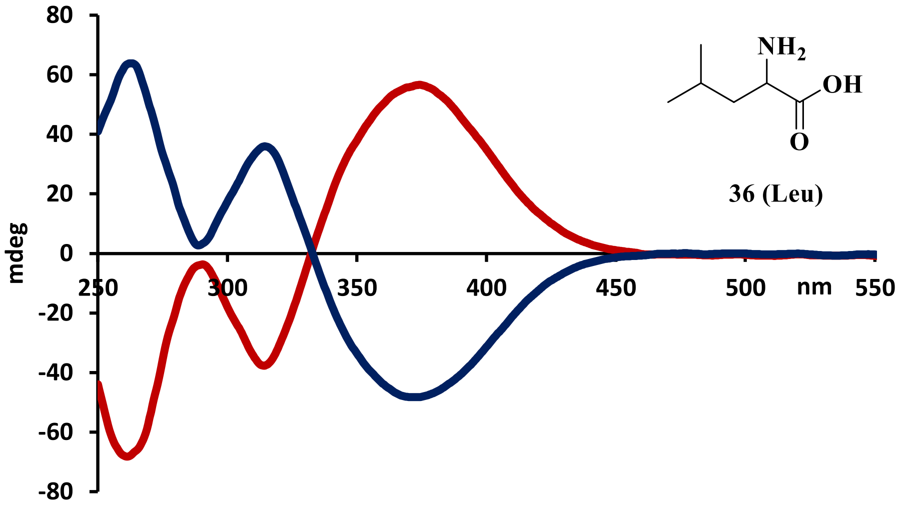

CD measurements were taken at 0.24 mM in acetonitrile.

Supplementary Figure 59: CD spectra obtained from probe **3** with (*S*)-**37** (red) and (*R*)-**37** **(**blue)

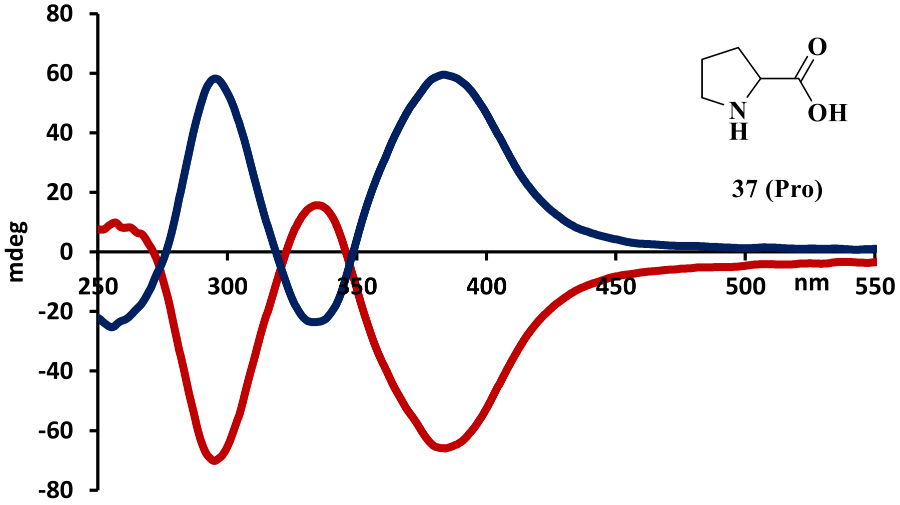

CD measurements were taken at 0.35 mM in acetonitrile.

Supplementary Figure 60: CD spectra obtained from probe **3** with (*S*)-**38** (red) and (*R*)-**38** **(**blue)

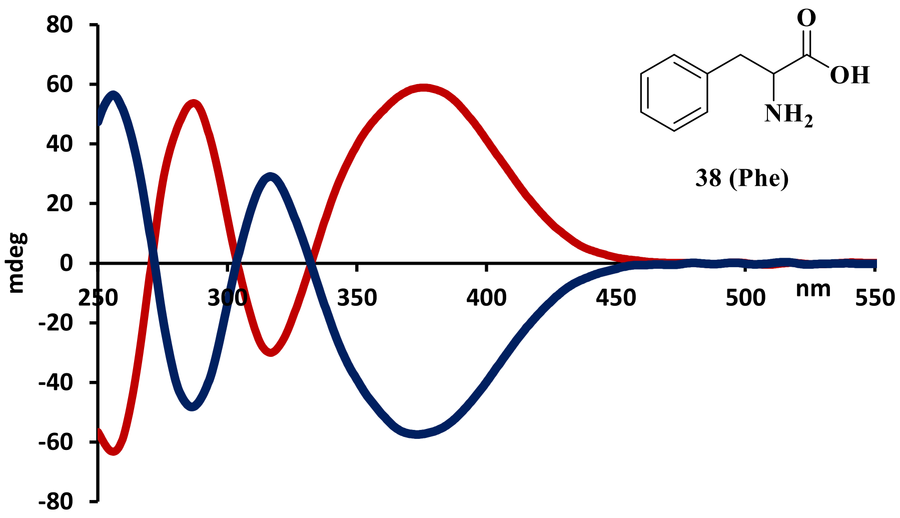

CD measurements were taken at 0.28 mM in acetonitrile.

Supplementary Figure 61: CD spectra obtained from probe **3** with (*S*)-**39** (red) and (*R*)-**39** **(**blue)

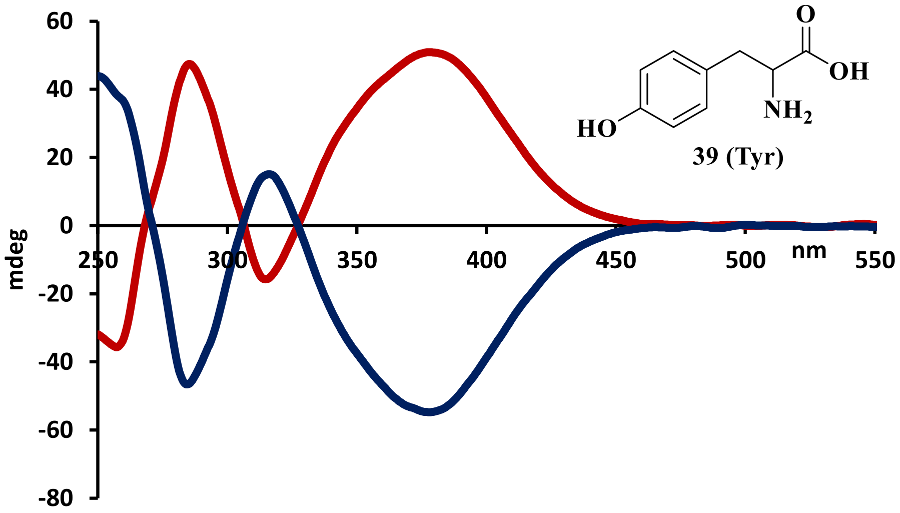

CD measurements were taken at 0.28 mM in acetonitrile.

Supplementary Figure 62: CD spectra obtained from probe **3** with (*S*)-**40** (red) and (*R*)-**40** **(**blue)

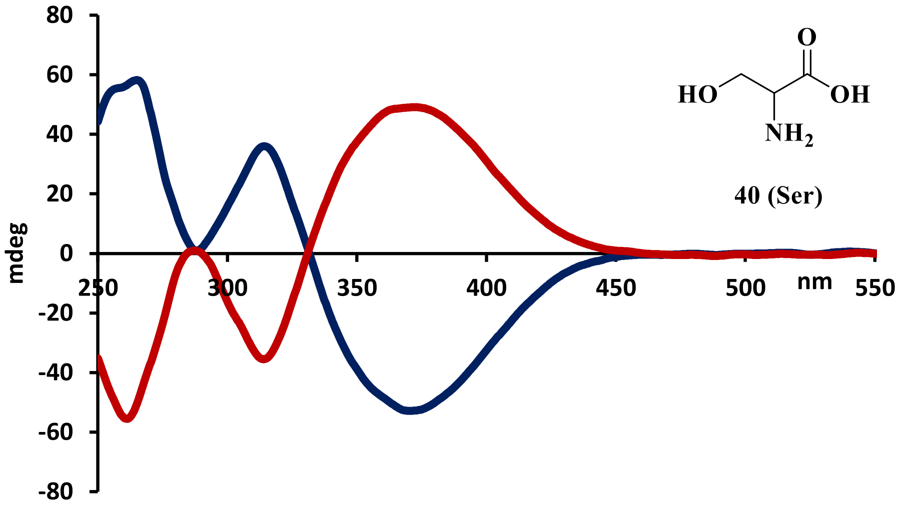

CD measurements were taken at 0.28 mM in acetonitrile.

Supplementary Figure 63: CD spectra obtained from probe **3** with (*S*)-**41** (red) and (*R*)-**41** **(**blue)

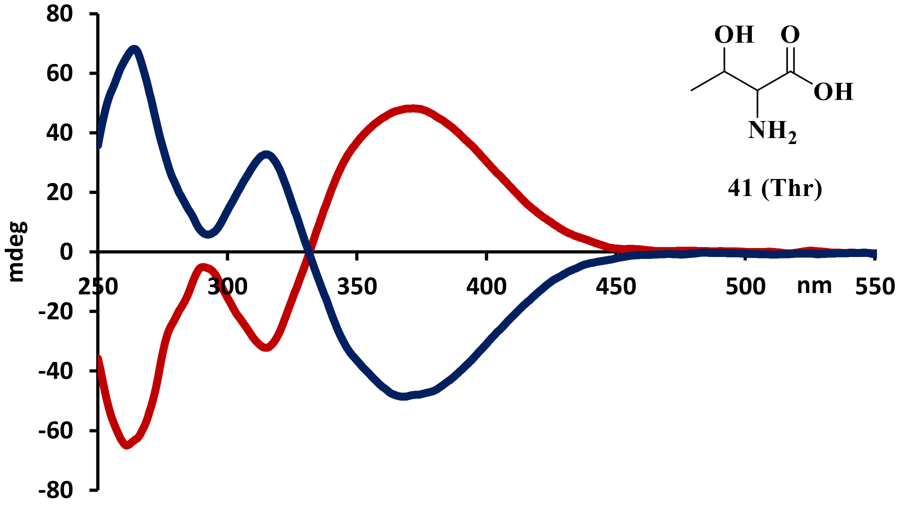

CD measurements were taken at 0.24 mM in acetonitrile.

Supplementary Figure 64: CD spectra obtained from *1 equivalent* of probe **3** (with (*S*)-**42** (red) and (*R*)-**42 (**blue)

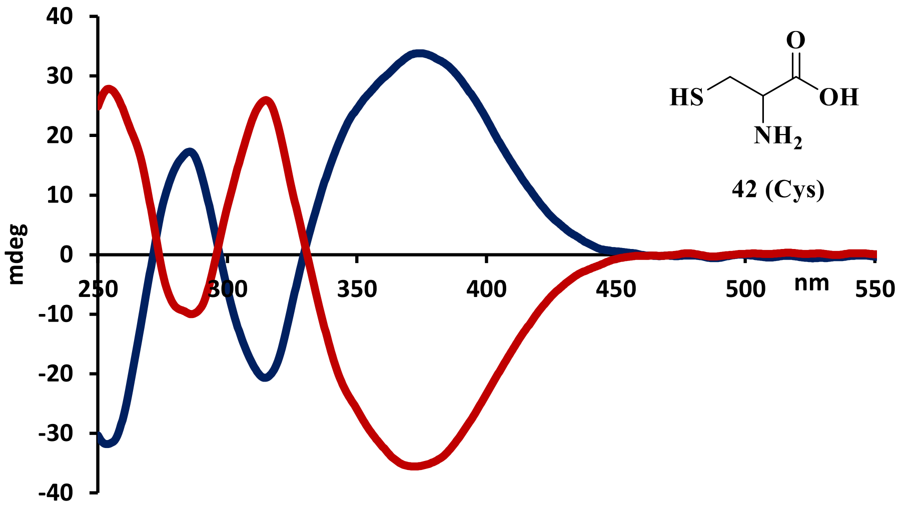

CD measurements were taken at 0.28 mM in acetonitrile.

Supplementary Figure 65: CD spectra obtained from *2 equivalents* of probe **3** (with (*S*)-**42** (red) and (*R*)-**42 (**blue

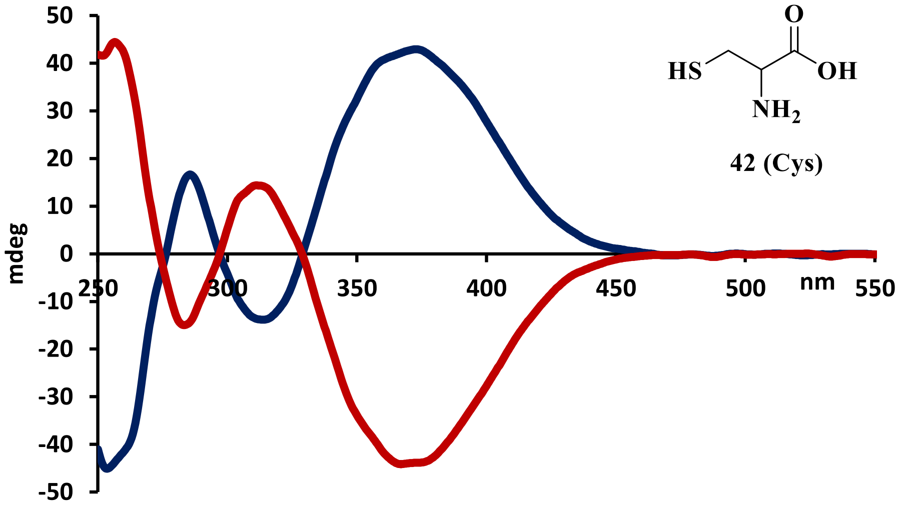

CD measurements were taken at 0.19 mM in acetonitrile.

Supplementary Figure 66: CD spectra obtained from probe **3** with (*S*)-**43** (red) and (*R*)-**43 (**blue)

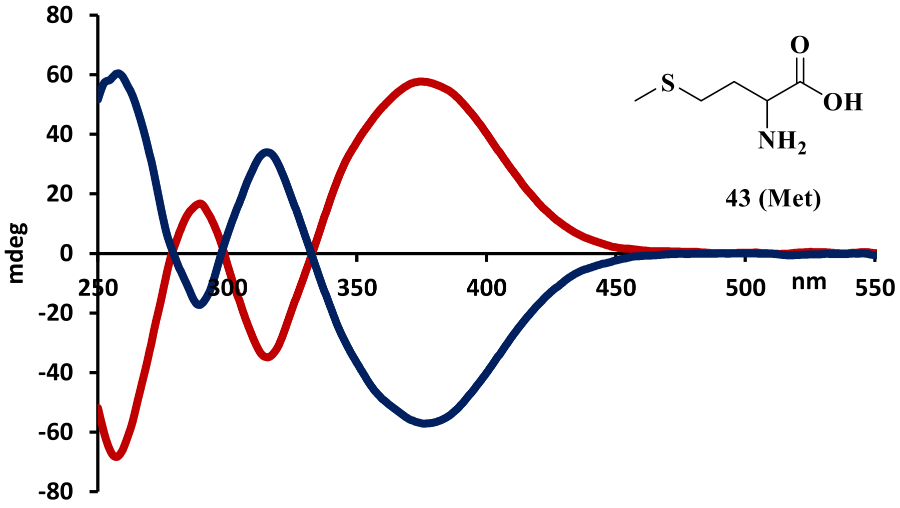

CD measurements were taken at 0.24 mM in acetonitrile.

Supplementary Figure 67: CD spectra obtained from probe **3** with (*S*)-**44** (red) and (*R*)-**44 (**blue)

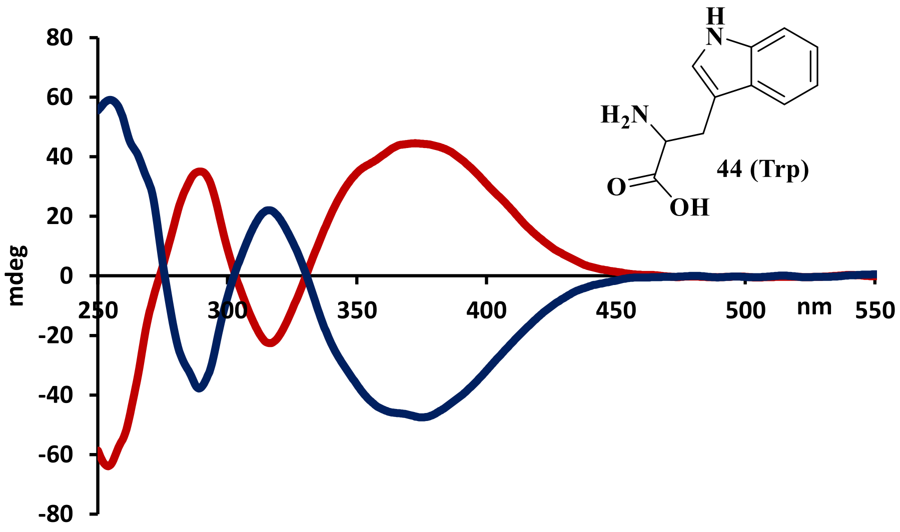

CD measurements were taken at 0.28 mM in acetonitrile.

Supplementary Figure 68: CD spectra obtained from probe **3** with (*S*)-**45** (red) and (*R*)-**45** (blue)

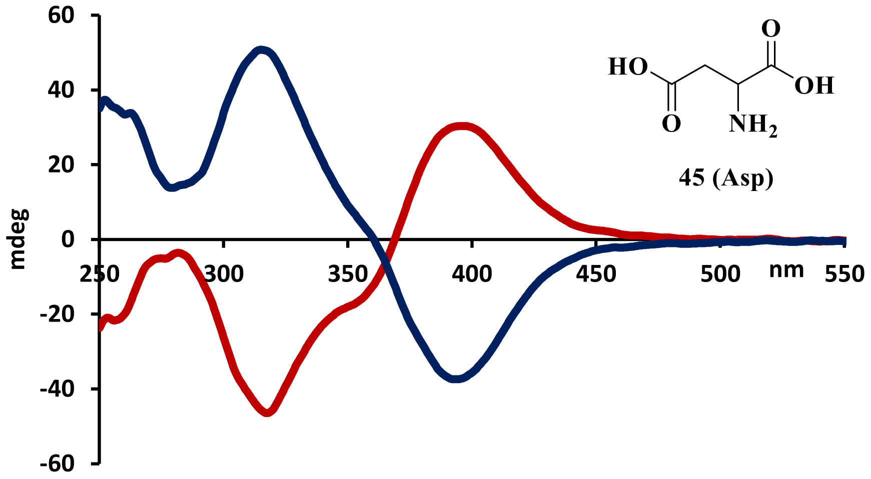

CD measurements were taken at 0.39 mM in acetonitrile.

Supplementary Figure 69: CD spectra obtained from probe **3** with (*S*)-**46** (red) and (*R*)-**46** (blue)

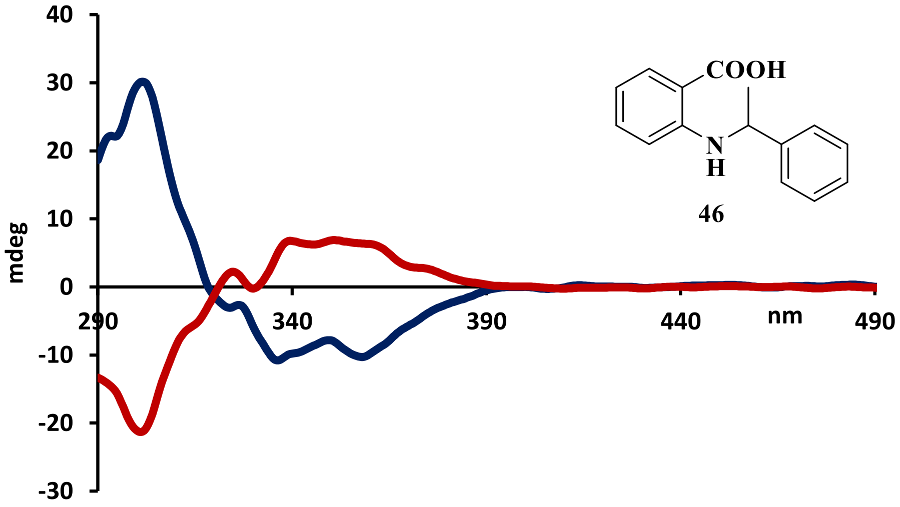

CD measurements were taken at 0.35 mM in chloroform.

Supplementary Figure 70: UV spectra obtained from the reaction between probe **3** and varying amounts of (*S*)-1-phenylethylamine (**10**)

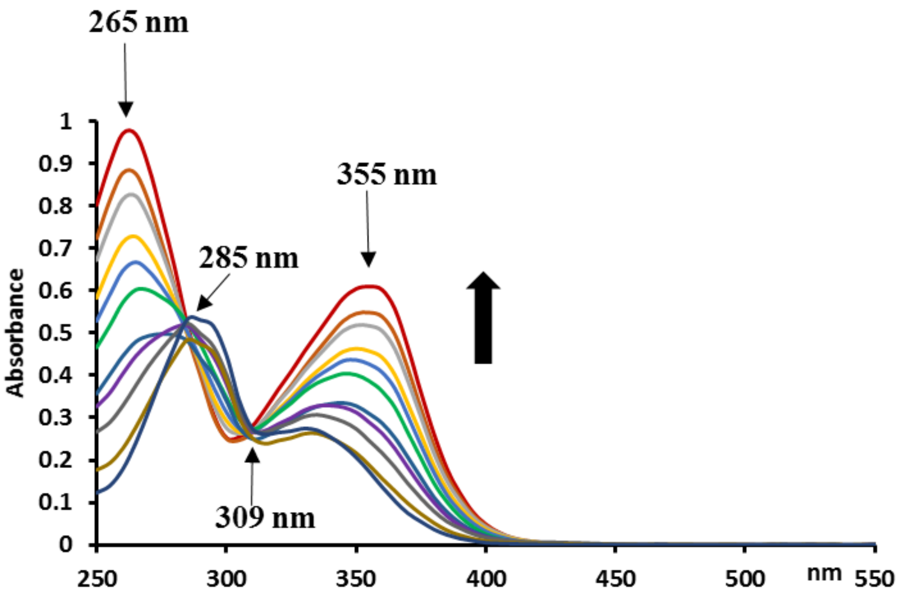

Supplementary Figure 71: $\frac{[A_{265} - A_{309}]}{A_{309}}$ ratio of the reaction mixture plotted against the concentration of (*S*)-1-(2-naphthyl)ethylamine (**10**)

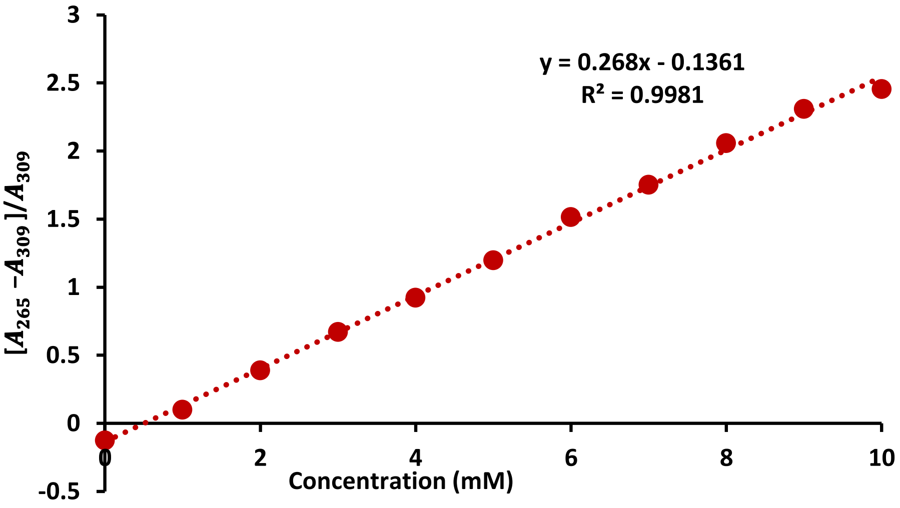

Supplementary Figure 72: Chiroptical response of probe **3** to scalemic samples of 1-(2-naphthyl)ethylamine (**10**)

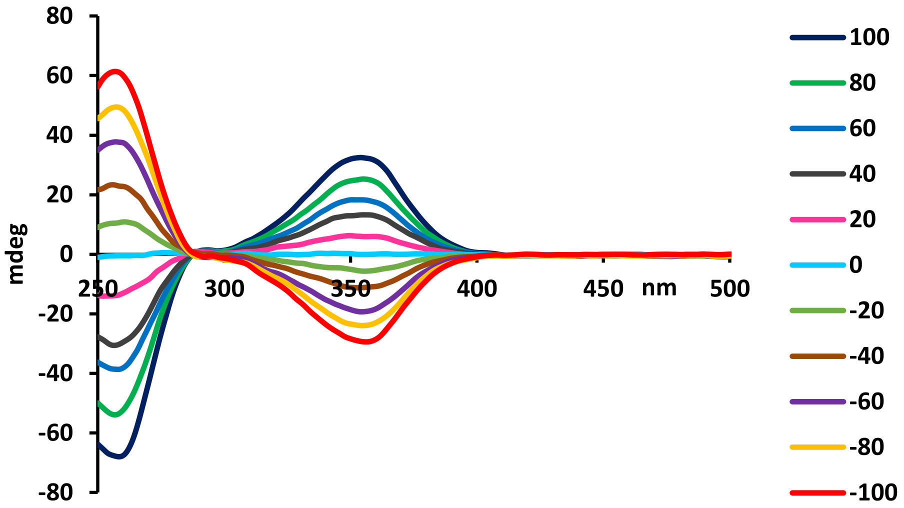

Supplementary Figure 73: Plot of the CD amplitudes at 257 nm (red) and 355 nm (blue) versus sample ee

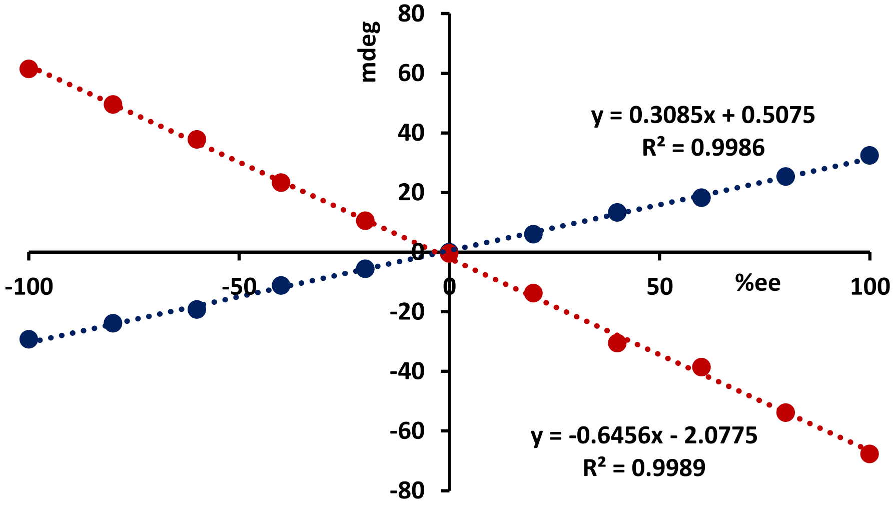

Supplementary Figure 74: Plot of the calculated vs actual values of concentration

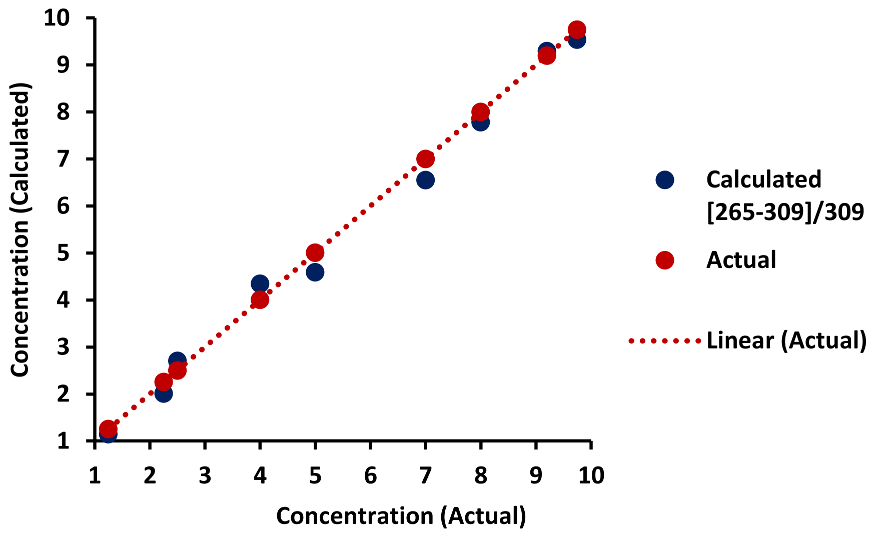

Supplementary Figure 75: UV spectra obtained from the reaction between probe **3** and varying amounts of (*S*)-*N*-methyl-1-phenylethylamine (**17**)

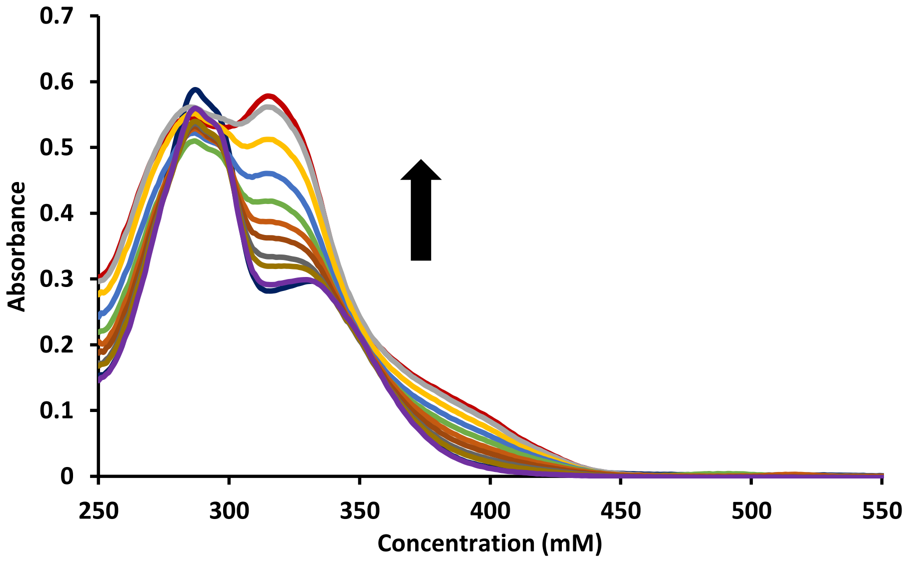

Supplementary Figure 76: Absorbance at 392 nm of the reaction mixture plotted against the concentration of (*S*)-*N*-methyl-1-phenylethylamine (**17**)

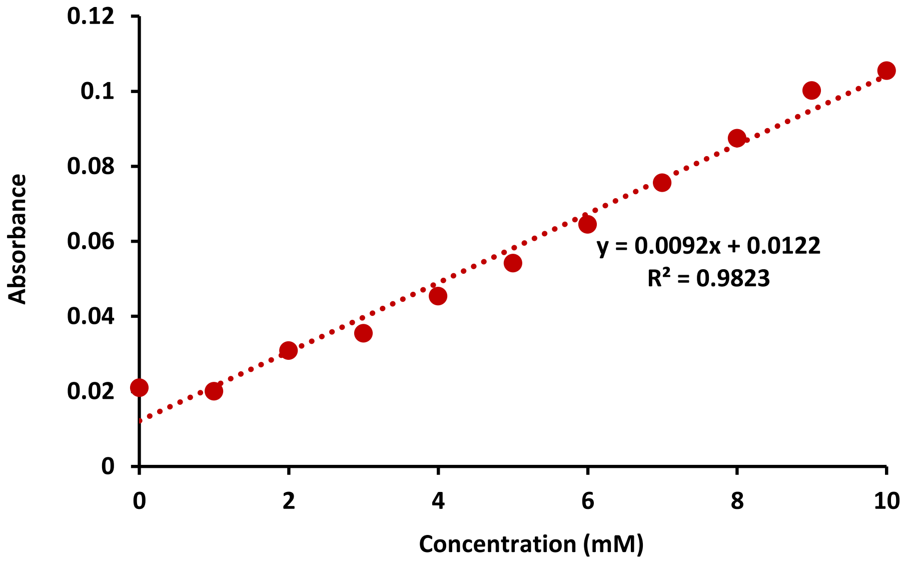

Supplementary Figure 77: Chiroptical response of probe **3** to scalemic samples of the *N*-methyl-1-phenylethylamine (**17**)

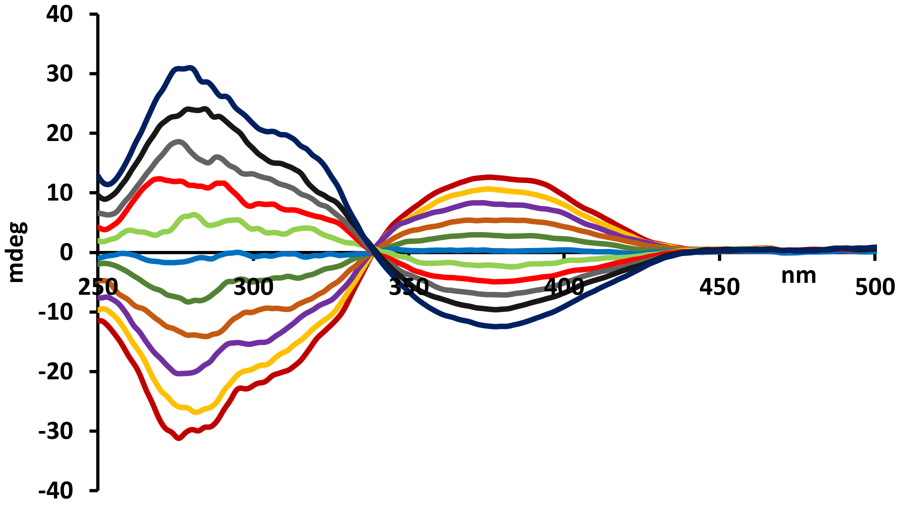

Supplementary Figure 78: Plot of the CD amplitudes at 376 nm versus sample enantiomeric excess

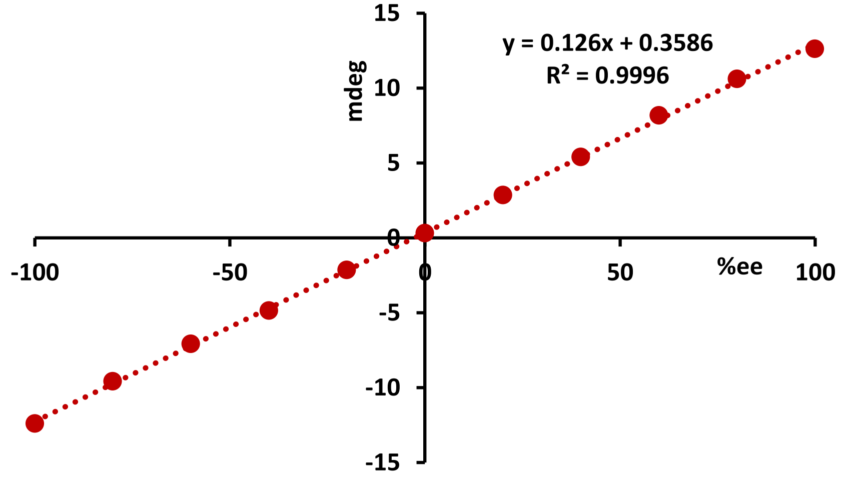

Supplementary Figure 79: Enantioseparation of (±) *N*-Boc-*N*-methyl-1-phenylethylamine

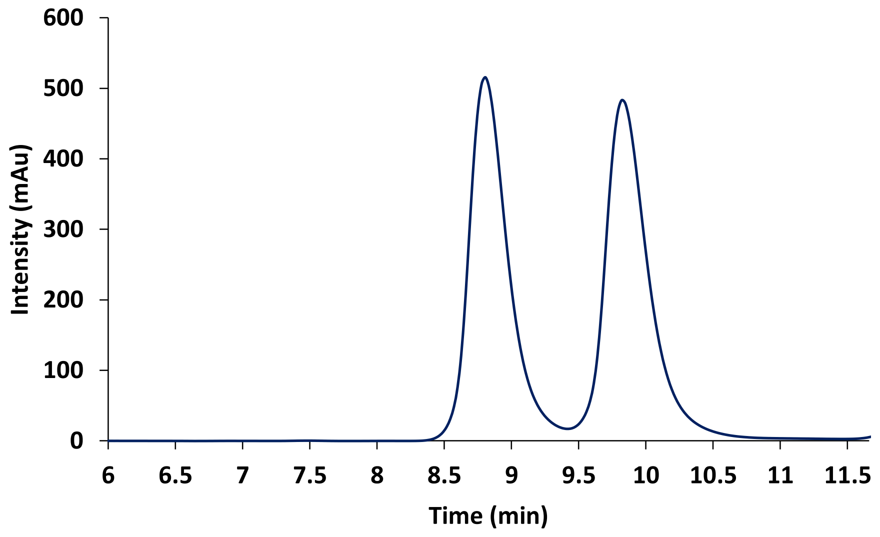

Supplementary Figure 80: HPLC of enantioenriched (*S*)-*N*-Boc-*N*-methyl-1-phenylethylamine

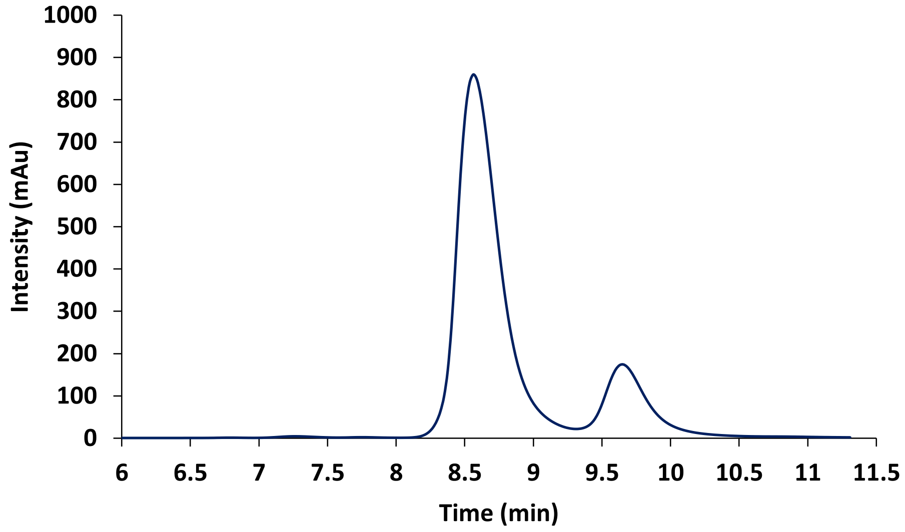

S,*S*-WhelkO using hexanes:IPA (99:1) as the mobile phase at 1.0 mL/min, t_R (major) = 8.6 min, t_R (minor) = 9.6 min

Supplementary Figure 81: Chiroptical response of probe **3** to scalemic samples of 1-phenylethylamine (**8**)

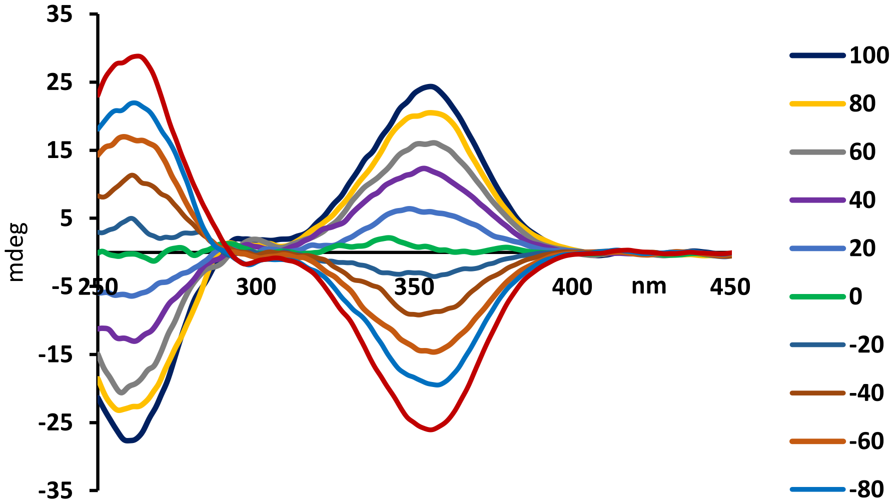

Supplementary Figure 82: Plot of the CD amplitudes at 340 nm versus sample ee

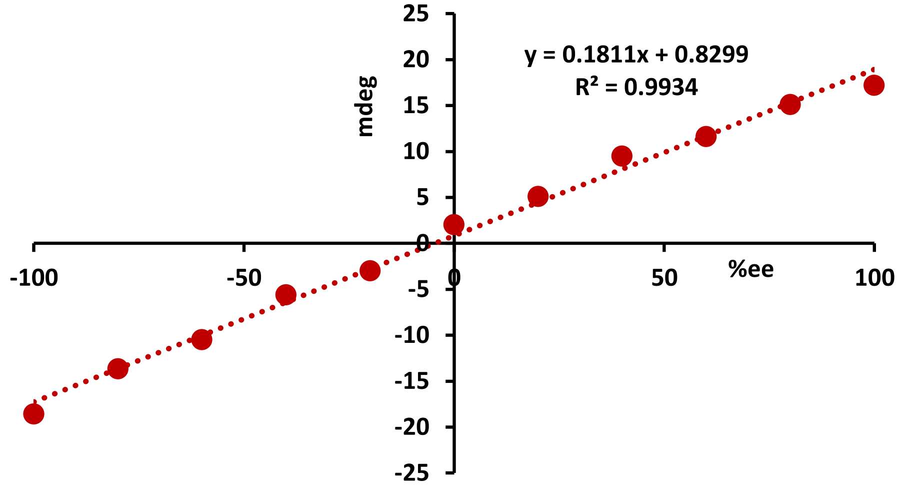

Supplementary Figure 83: Chiroptical response of probe **3** to scalemic samples of *N*-methyl-1-phenylethylamine (**17**)

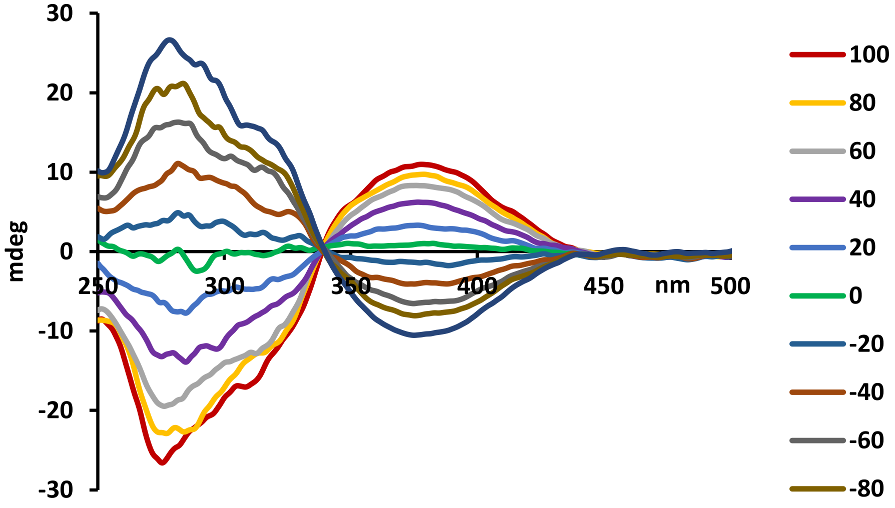

Supplementary Figure 84: Plot of the CD amplitudes at 410 nm versus sample ee

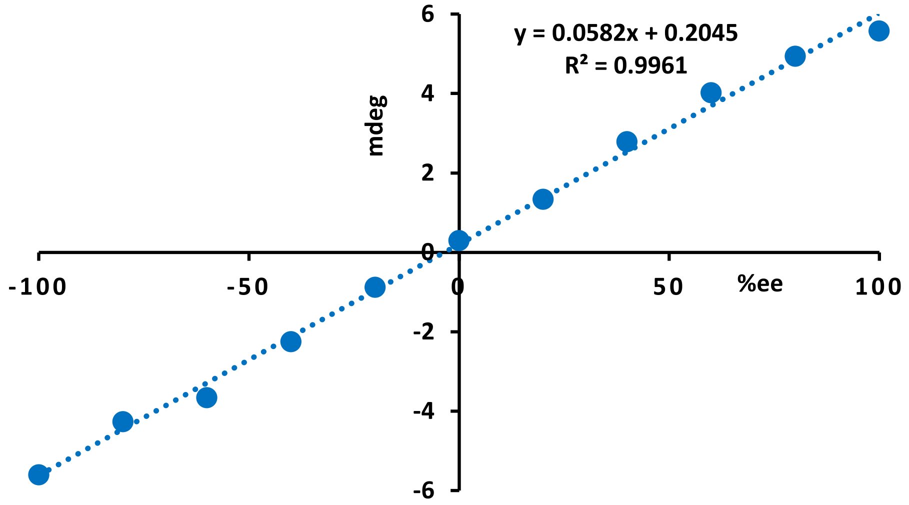

Supplementary Figure 85: X-ray structure of 4-chlorocoumarin **(1)**

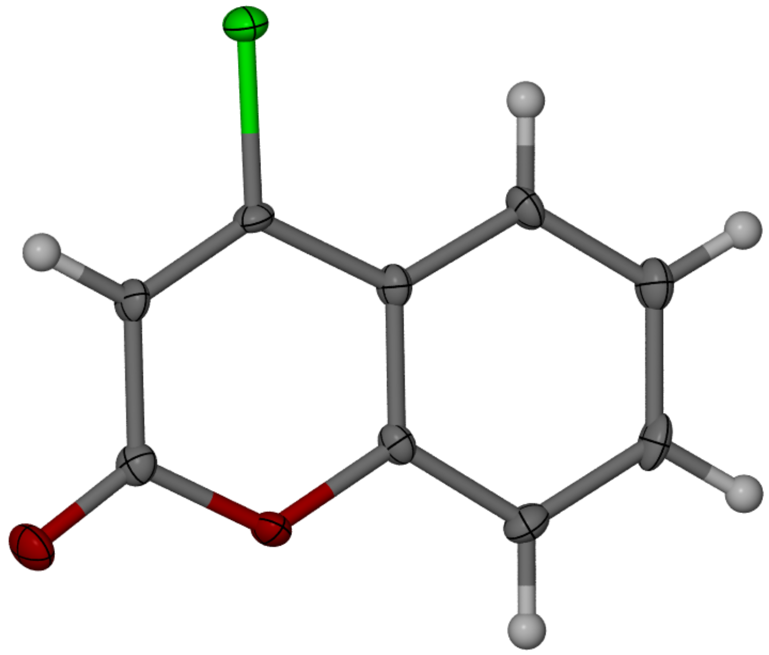

Supplementary Figure 86: X-ray structure of 4-bromocoumarin **(2)**

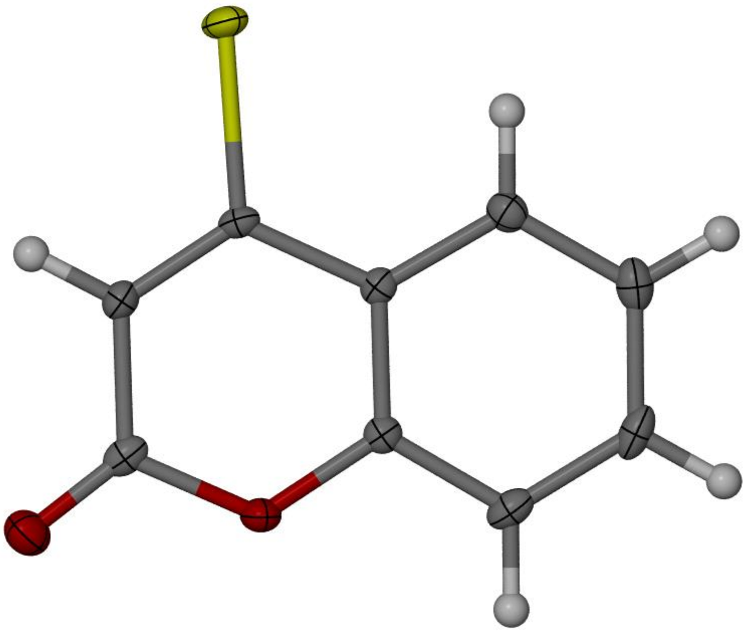

Supplementary Figure 87: X-ray structure of 4-iodo-3-nitrocoumarin **(5)**

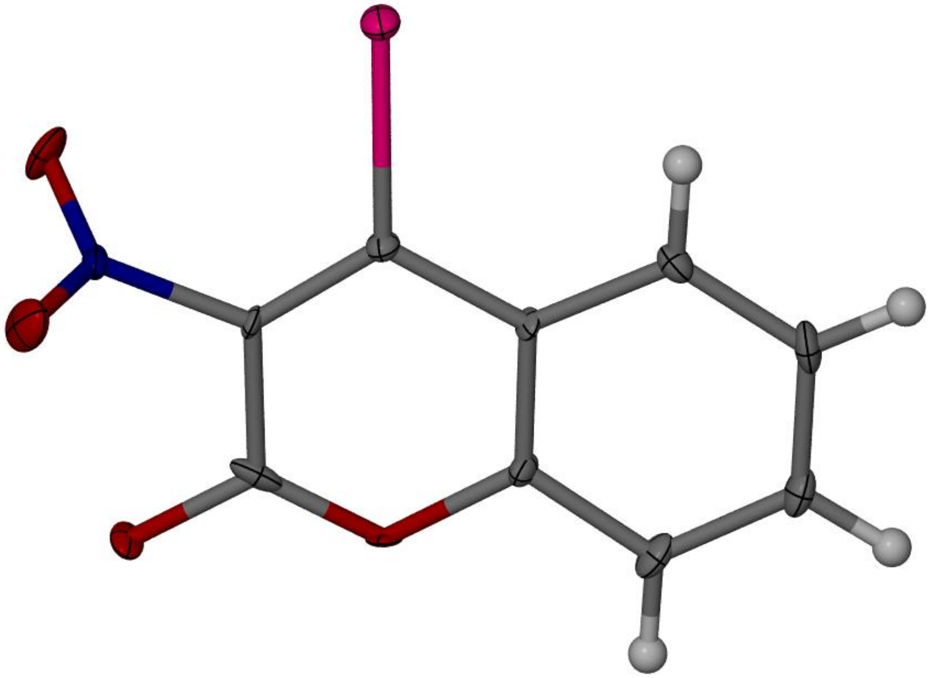

Supplementary Figure 88: X-ray structure of (*S*)-3-nitro-4-((1-phenylethyl)amino)coumarin **(7)**

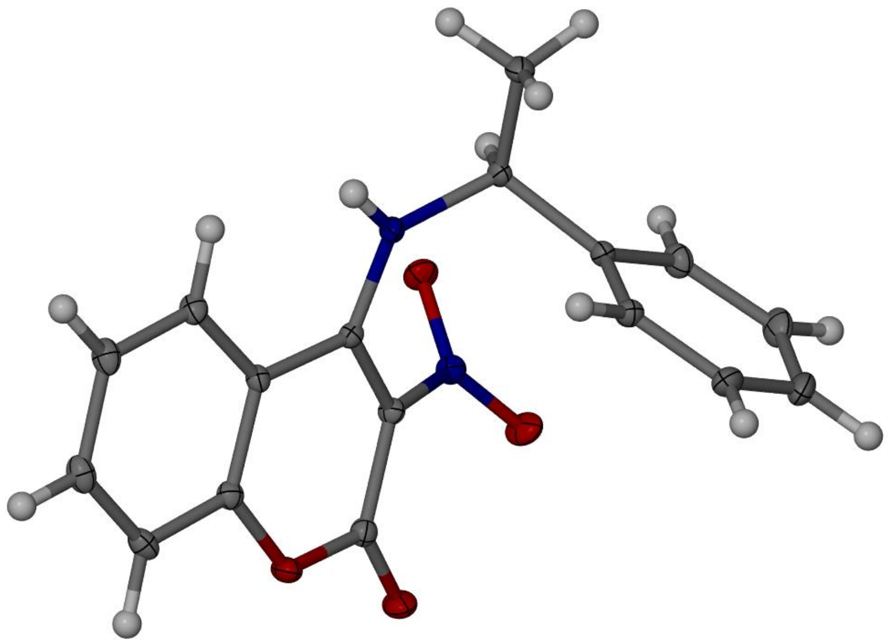

Supplementary Figure 89: X-ray structure of (*R*)-3-nitro-4-(*N*,α-dimethylbenzyl)amino)coumarin

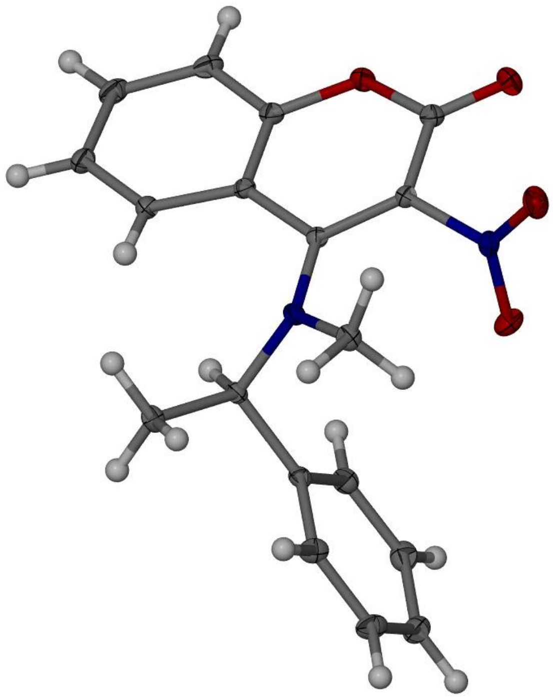

Supplementary Tables

Supplementary Table 1: Concentration, ee and absolute configuration of samples of 1-(2-naphthyl)ethylamine (**10**) determined by the combined UV and CD responses of probe **3**

Sample composition			Sensing results				
Abs. Config.	Concentration (mM)	%ee	Abs. Config.	Concentration by $\frac{[A_{265} - A_{309}]}{A_{309}}$ (mM)	%ee at 355 nm	%ee at 257 nm	Average %ee
R	4.00	25.0	*R*	4.34	24.7	23.4	24.0
R	2.25	55.5	*R*	2.01	56.9	56.0	56.5
S	5.00	50.0	*S*	4.59	54.1	54.7	54.4
R	9.20	8.0	*R*	9.29	11.1	11.9	11.5
S	2.50	33.3	*S*	2.70	35.5	36.6	36.0
R	7.00	42.8	*R*	6.55	46.6	47.1	46.8
S	8.00	37.5	*S*	7.78	41.7	43.5	42.6
S	9.75	79.0	*S*	9.54	84.7	81.3	83.0
S	1.25	60.0	*S*	1.14	52.5	56.5	54.5

Supplementary Table 2: Enantiomeric excess and conversion of the asymmetric hydrogenation of *N*-methyl-1-phenylethan-1-imine (**48**)

			Traditional analysis			Chiroptical sensing		
Ligand	Catalyst Loading (mol%)	Time (h)	Abs. Config.	%ee by HPLC	Conv. (%) (by ^{1}H NMR)	Abs. Config.	%ee by CD at 376 nm	Conv. (%) UV at 392 nm
**49**	5.00	18	*S*	55.8[a]	99.9	*S*	59.8	96.0
**50**	5.00	18	*R*	16.3[a]	99.9	*R*	14.3	99.9
**51**	5.00	18	*R*	31.0[a]	99.9	*R*	32.2	99.1
**52**	5.00	18	*R*	31.4[a]	99.9	*R*	25.8	98.0
**53**	5.00	18	*S*	16.3[a]	92.0	*S*	14.2	96.3
**49**	2.50	1	*S*	46.2[b]	51.1	*S*	47.8	53.9
**49**	3.25	1	*S*	57.3[b]	63.3	*S*	54.5	68.4

Conv. = conversion

[a] *S,S*-Whelk-O: Hexane:IPA = 99:1, flow rate = 1.0 mL/min, UV= 214 nm, t_R = 8.6 min (major) and t_R = 9.6 min (minor). [b] *R,R*-Whelk-O, Phenomenex® Lux 5 µm Amylose-2 (connected in series): Hexane:IPA = 99:1, flow rate = 0.8 mL/min, UV= 214 nm, t_R = 17.9 min (minor) and t_R = 19.9 min (major).

Supplementary Table 3: Enantiomeric excess and absolute configuration of samples of 1-phenylethylamine (**8**) and *N*-methyl-1-phenylethylamine (**17**) determined by the CD responses of probe **3**

Actual sample composition				Sensing results			
Amine **8**		Amine **17**		Amine **8**		Amine **17**	
%ee	Abs. Config.	%ee	Abs. Config.	%ee by CD at 340 nm	Abs. Config.	%ee by CD at 410 nm	Abs. Config.
-90.0	*R*	+10	*S*	-93.0	*R*	+05.0	*S*
-70.0	*R*	+30	*S*	-76.2	*R*	+25.3	*S*
-50.0	*R*	+50	*S*	-48.3	*R*	+48.9	*S*
-30.0	*R*	+70	*S*	-26.5	*R*	+66.6	*S*
-10.0	*R*	+90	*S*	-04.0	*R*	+82.4	*S*
+10.0	*S*	-90	*R*	+06.5	*S*	-88.1	*R*
+30.0	*S*	-70	*R*	+30.6	*S*	-72.4	*R*
+50.0	*S*	-50	*R*	+55.2	*S*	-56.6	*R*
+70.0	*S*	-30	*R*	+74.9	*S*	-34.3	*R*
+90.0	*S*	-10	*R*	+93.1	*S*	-12.5	*R*

Supplementary Equations

Using the ratio of, $y = \frac{[A_{265} - A_{309}]}{A_{309}}$;

$x = \frac{(y+0.1361)}{0.268}$ (Supplementary Equation 1); x in mM)

At 355 nm;

$ee = \frac{(\frac{(mdeg \times 5)}{x} - 0.5075)}{0.3085}$ (Supplementary Equation 2)

At 422 nm;

$ee = \frac{(\frac{(mdeg \times 5)}{x} + 2.0775)}{(-0.6456)}$ (Supplementary Equation 3)

Using the absorbance measured at 392 nm = y

$x = \frac{(y+0.0122)}{0.0092}$ (Supplementary Equation 4; x in mM)

At 392 nm;

$ee = \frac{(\frac{(mdeg \times 5)}{x} - 0.3586)}{0.126}$ (Supplementary Equation 5)

Enantiomeric excess of 1-phenylethylamine (**8**);
Using the CD intensity measured at 340 nm = y
$x = \frac{(y-0.8299)}{0.1811}$ (Supplementary Equation 6; x = %ee of 1-phenylethylamine (**8**))

Enantiomeric excess of *N*-methyl-1-phenylethylamine (**17**);
Using the CD intensity measured at 410 nm = y
$x = \frac{(y-0.2045)}{0.0582}$ (Supplementary Equation 7; x = %ee of *N*-methyl-1-phenylethylamine (**17**))

Supplementary References

1. Wakchaure, V. N., Kaib, P. S. J., Leutzsch, M. List, B. Disulfonimide-Catalyzed Asymmetric Reduction of *N*-Alkyl Imines. *Angew. Chem. Int. Ed.*, *54*, 11852-11856 (2015).
